# Synthesis of Methoxylated Benzoxanthones as Drug Metabolites of Antischistosomal Schistodiones—A Limited Environmental Risk

**DOI:** 10.3390/molecules31111839

**Published:** 2026-05-27

**Authors:** Elena Cesar-Rodo, Jeremy Boilevin, Jimmy Richard, Peter D. Ziniel, Didier Belorgey, Louis Maes, Francesco Angelucci, David Lee Williams, Elisabeth Davioud-Charvet, Don Antoine Lanfranchi

**Affiliations:** 1 Laboratoire d’Innovation Moléculaire et Applications (LIMA), UMR7042 CNRS-Université de Strasbourg-Université Haute-Alsace, Team Bio(IN)organic & Medicinal Chemistry, European School of Chemistry, Polymers and Materials (ECPM), 25, Rue Becquerel, F-67087 Strasbourg, France; elena.cesar.rodo@etu.unistra.fr (E.C.-R.); jeremy.boilevin@etu.unistra.fr (J.B.); jrichards@unistra.fr (J.R.); dbelorgey@unistra.fr (D.B.); 2 Department of Microbial Pathogens and Immunity, Rush University Medical Center, 1735 West Harrison Street, Chicago, IL 60612, USA; petezii2001@yahoo.com (P.D.Z.); david_williams@rush.edu (D.L.W.); 3 Laboratory of Microbiology, Parasitology and Hygiene (LMPH), Faculty of Pharmaceutical, Biomedical and Veterinary Sciences, University of Antwerp, Universiteitsplein 1, B-2610 Antwerp, Belgium; louis.maes@uantwerpen.be; 4 Department of Life, Health and Environmental Sciences, University of L’Aquila, 67100 L’Aquila, Italy; francesco.angelucci@univaq.it

**Keywords:** antiplasmodial, antischistosomal, benzoxanthone, 3-benzylmenadione, oxidative phenolic coupling, prodrug, nucleophilic aromatic substitution S_N_A_r_

## Abstract

In the search for new antischistosomal 3-benzylmenadiones (benzylMDs), the screening of a library developed in our laboratory led to the identification of two regioisomeric analogues, the 2′,5′- and 3′,5′-dimethoxy-benzylMD—designated schistodiones A_2′,5′_ and A_3′,5′_—which were investigated for their activity against the platyhelminth Schistosoma mansoni and various protozoan parasites, bacteria, and fungi. Reported work has shown that benzylMDs act as prodrugs: their bioactivation undergoes a cascade of redox reactions within the parasite, generating multiple drug metabolites, e.g., the main benzoylmenadione (benzoylMD) intermediates, and reactive oxygen species that interfere with key metabolic pathways. Among the secondary metabolites, benzoxanthones have been identified as potential products generated along this oxidative pathway. The aim of the study was to synthetize methoxylated benzoxanthones, as putative metabolites generated from these antischistosomal benzylMDs. During the synthetic work, several difficulties arose, including the absence of starting reagents, the incompatibility of certain reactions with methoxy groups, the possible formation of several isomers, and the easy re-oxidation of sensitive intermediates. To overcome these obstacles, we developed a new retrosynthetic strategy using modified precursors: replacing methoxy groups with O-methylenemethoxy (OMOM) groups that are more stable in basic media, using aldehydes or aromatic esters as precursors, and replacing certain substituents with groups that are easier and less costly to introduce (chlorine or nitro). Selected metabolites (benzoylMDs, benzoxanthones) were then tested in parasite and cellular assays. Furthermore, benzoylMDs were tested as subversive substrates of *S. mansoni* thioredoxin-glutathione reductase (*Sm*TGR) and selected drug metabolites were investigated in SmTGR modeling experiments. From a One Health perspective, these benzylMD derivatives pose limited environmental risk because their metabolites lack toxicity when encountered externally, as toxicity requires intracellular metabolic activation and localized formation of reactive intermediates in close proximity to their cellular targets inside parasites.

## 1. Introduction

The One Health concept is a collaborative approach that integrates human, animal, and environmental health to address complex health issues. It emphasizes the fact that these domains are interconnected and there is a need for interdisciplinary efforts to achieve optimal health outcomes. This concept is particularly relevant to drug metabolites, as it highlights the potential for metabolites to affect not only human health but also animal health and environmental health. The One Health approach is essential for understanding the broader implications of drug metabolites, including their impact on toxicity, efficacy, resistance mechanisms, and long-term health risks across different species and ecosystems. It recognizes that health challenges in one domain can have cascading effects on others, making it crucial to consider the holistic impact of drug metabolites. The concept also underscores the importance of collaborative efforts to mitigate these impacts, which is particularly pertinent to the study of drug metabolites generated from chemotherapy in use. To gather more detailed information and broader implications of drug metabolites, including their effects on toxicity, efficacy, resistance mechanisms, and long-term health risks across various species and ecosystems, it is first necessary to anticipate their structures and to synthetize them. However, the synthesis of drug metabolites can be more complex than expected.

In the search for new antischistosomal drugs, we screened a library of antiparasitic 3-benzylmenadiones (benzylMDs) designed in our laboratory. Two benzylMD regioisomers were investigated for their action on the platyhelminth, *Schistosoma mansoni*, i.e., the 2,5- and 3,5-dimethoxy-benzylMD analogues, called schistodiones A_2′,5′_ and A_3′,5′_ [1,2].

**Scheme 1:**
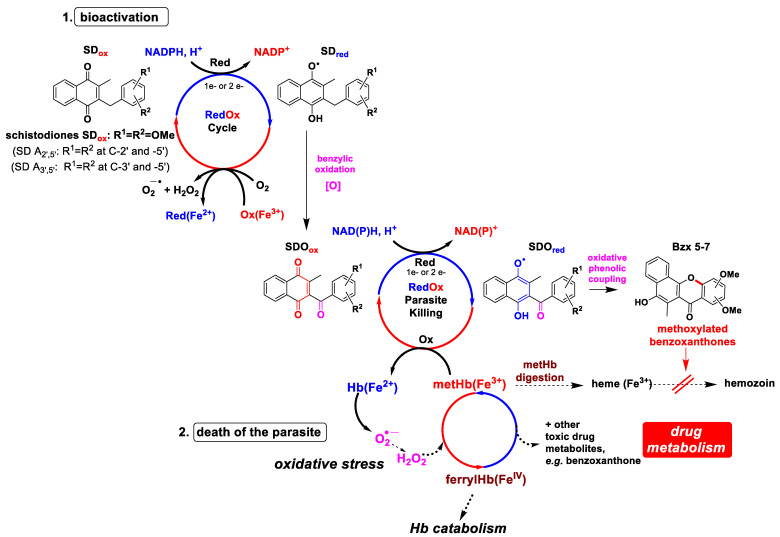
Drug metabolism pathways via a cascade of redox reactions generating benzylMD-derived metabolites, including the methoxylated benzoxanthones.

3-BenzylMDs act as prodrugs: they enter a cascade of redox reactions generating various metabolites acting at different choke points in the parasitic metabolic pathways. Aside from the key 3-benzoylmenadione (benzoylMD) metabolites that enter into the redox-cycling, other potential benzoylMD-derived metabolites were demonstrated to be formed along the flux of reactive oxygen species (ROSs), such as the benzoxanthones [3,4], or the recently described anthraquinones [5]. In adult worms, the druggable Thioredoxin-Glutathione Reductase (*Sm*TGR), along with other flavoenzymes may contribute to the general mechanism of action of benzylMD (through benzoylMD metabolites) depicted in Scheme 1. As this enzyme is major in schistosomes and the human orthologue is expressed at very low levels in humans [6], this feature is particularly relevant for the specific bioactivation of antischistosomal benzylMDs in *S. mansoni*, making these chemical series particularly safe for humans and the environment.

## 2. Previous Works

### 2.1. Synthesis of the Benzoxanthone Derived from the Antiplasmodial Plasmodione

**Scheme 2:**
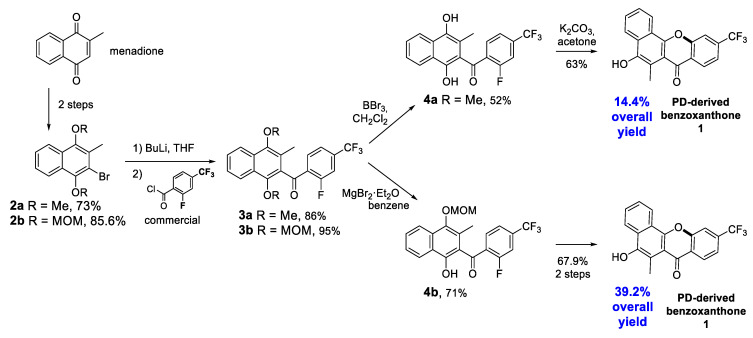
Reported synthetic routes for the preparation of benzoxanthone **1** derived from the antiplasmodial plasmodione.

Plasmodione (PD)-derived benzoxanthone **1** was first synthesized in the laboratory from menadione (Scheme 2) [3]. In this reported route, 2-bromo-3-methyldimethoxynaphthalene **2a** is synthesized in two steps according to the procedure described by Bauer et al. [7]. It is then coupled with commercial 2-fluoro-(4-trifluoromethyl)-benzoyl chloride and the acylated product **3a** deprotected with BBr_3_ to obtain the dihydroquinone **4a**. Treatment of this intermediate with K_2_CO_3_ in acetone leads to the formation of PD-derived benzoxanthone **1** via an intramolecular nucleophilic aromatic substitution (S_N_Ar). This synthesis involves five steps with an overall yield of 14.4% [3]. Then, we set up an improved route to access the PD-derived benzoxanthone **1** by changing the protection of the hydroxyl groups of the reduced naphthoquinone core by O-dimethylenedimethoxy (OMOM) groups (replacing OMe groups) and by selective mono-deprotection of the hydroxyl group at C-4 to favor an efficient nucleophilic aromatic substitution from **4** [4]. It is noteworthy that only the OMOM group in β-position to the C=O of the benzoyl chain can be deprotected by MgBr_2_·Et_2_O in benzene because of the chelating effect [8]. The overall yield of the second synthetic route was 39.2% (from menadione, six steps, Scheme 2).

### 2.2. This Work: Synthesis of the Methoxylated Benzoxanthones ***5***–***7*** Derived from the Antischistosomal Schistodiones A_2′,5′_ and A_3′,5′_

The objective of this work has been the synthesis of methoxylated benzoxanthones **5**–**7** from antischistosomal lead benzylMDs, henceforth called schistodiones A_2′,5′_ and A_3′,5′_ [1]. Because the methoxy group also behaves as leaving group in the oxidative phenolic coupling, we envisioned that two benzoxanthones could be generated from the schistodione A_2′,5′_ in the parasite.

### 2.3. Synthesis Strategy

When performing the retrosynthesis of these methoxy-benzoxanthones based on the synthesis of PD-derived benzoxanthone **1**, we very quickly encountered the following issues: (1) methoxylated and fluorinated acid chlorides were not known in the literature and were not commercially available when we started this work; (2) the use of demethylation agents such as BBr_3_ is totally incompatible with the methoxy groups on the benzene ring of the benzylic chain; (3) the oxidative phenolic coupling of the schistodione oxide SDO_red_ A_2′,5′_, under its reduced form, can generate two benzoxanthone regioisomers; (4) several previous experiments have shown that the reduced benzoylMD (benzoylMD_red_) intermediates, prepared to be cyclised to benzoxanthones, re-oxidise too easily in air by regenerating the quinone species (benzoylMD_ox_).

**Scheme 3:**
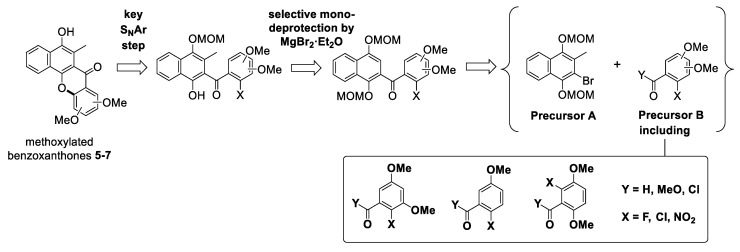
Retrosynthetic route to prepare methoxylated benzoxanthones **5**–**7** derived from schistodiones via the key aromatic nucleophilic substitution (S_N_Ar) and selective mono-OMOM deprotection by using MgBr_2_·Et_2_O in benzene.

In order to overcome these synthetic difficulties, a new retrosynthesis scheme has been developed using slightly modified precursors. The methoxy groups of bromodimethoxynaphthalene were replaced by OMOM groups, which are highly resistant in basic media but fairly easily cleavable in acidic conditions. In addition, the benzenic precursors B could be benzaldehydes (Y = H) or esters (Y = OMe) and are not limited to acid chlorides. The leaving group X can be a chlorine atom or a nitro group, which is easier to introduce into an aromatic series than a fluorine atom and much less expensive (Scheme 3).

### 2.4. Synthesis of New Precursors

The preparation of precursor A has already been reported [4] but the process has been improved in this work. The preparation of new precursors with general structure B is detailed below.

#### 2.4.1. Synthesis of 2-Bromo-menadione **8**

**Scheme 4:**
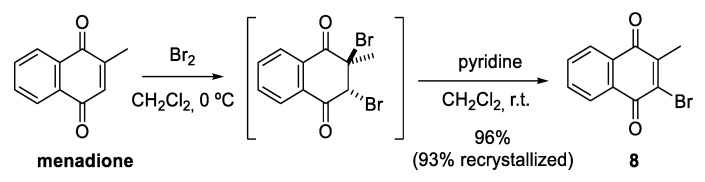
Multigram synthesis of 2-Bromo-menadione **8**.

2-Bromo-menadione **8** is synthesized by bromination of menadione according to a not previously described protocol. The only synthesis method previously described was in the early 1940s [9] by Br_2_, AcONa in acetic acid for 3 days with a yield of 71%. It is noteworthy to mention that this protocol has been repeated, over decades, unchanged, by numerous authors. However, when bromination is carried out by Br_2_ in CH_2_Cl_2_ to form the dibrominated intermediate, followed by addition of pyridine, the reaction yield reaches 96%. In addition, it is possible to obtain 20 to 30 g of product **8** in a few hours (Scheme 4).

#### 2.4.2. Synthesis of the diOMOM-Protected Naphthalene Precursor

**Scheme 5:**
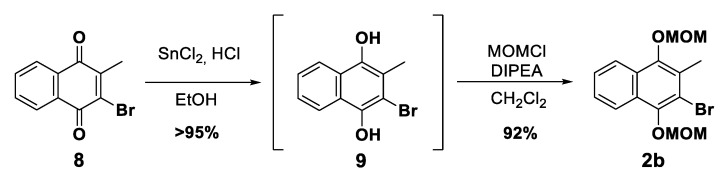
Synthesis of the 2-bromo-3-methyl-dimethylenemethoxy-naphthalene **2b**.

2-Bromo-menadione **8** is reduced to dihydroquinone **9** by SnCl_2_/HCl in ethanol. The product is precipitated in ice water to give a white solid, which quickly turns purple on contact with air. This coloration is certainly due to the formation of trace amounts of the semiquinone radical. This intermediate **9** is highly sensitive to oxidation. It is dried by azeotropic evaporation with toluene previously saturated with argon (3 × 40 mL/g of product). This tedious drying process limits the possibility of producing batches of precursor **2b** greater than 5–6 g. Once the dihydroquinone **9** is dry, it is deprotonated with DIPEA in CH_2_Cl_2_ (previously saturated with argon) and protected in the form of diOMOM-protected naphthalene **2b** using MOM chloride (Scheme 5). This method enabled the synthesis of the diOMOM-protected precursor **2b** with a yield of 85.6% in two steps.

**Scheme 6:**
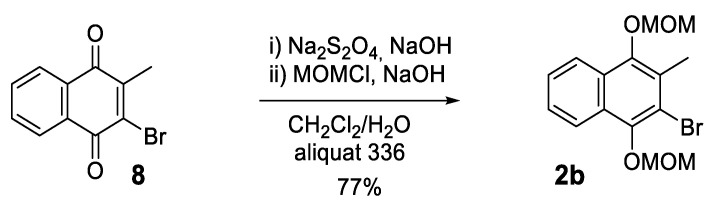
One-pot Synthesis of 2-bromo-3-methyl-dimethylenemethoxy-naphthalene **2b**.

An alternative synthesis route for **2b** was explored to reduce environmental impact, in order to avoid the laborious azeotropic drying of **9** using toluene, and in the hope of being able to produce larger quantities [10]. This involves a one-pot reduction/protection by phase transfer catalysis. 2-Bromo-menadione **8** is first reduced in a basic medium by dithionite in a two-phase mixture of CH_2_Cl_2_/water (previously saturated with argon) and 0.1 equiv. of aliquat 336 (Scheme 6). Two hours later, MOM chloride and a 3 M sodium hydroxide solution previously saturated with argon are added to give **2b** with 77% yield.

#### 2.4.3. Improved Synthesis of PD-Derived Benzoxanthone **1** from Precursor **2b**

**Scheme 7:**
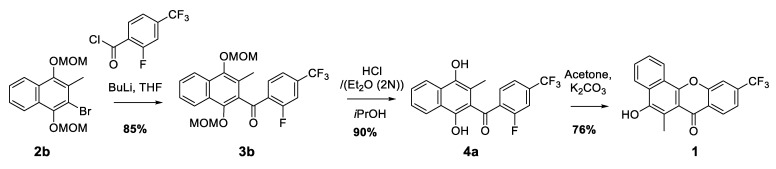
Improved synthesis of PD-derived benzoxanthone **1** from precursor **2b**.

From precursor **2b**, PD-derived benzoxanthone **1** was synthesized with a significant improvement in overall yield (Scheme 7) compared to the route using the dimethoxynaphthalene precursor **2a** (50.8% vs. 14.4% [3] from menadione; five steps as drawn in Scheme 2). This synthesis of benzoxanthone **1** via di-OMOM-deprotection starting from the newly produced 2-bromo-3-methyl-diOMOM-naphthalene **2b** is also improved—with an overall yield of 49.8% in six steps from menadione—when comparing the preliminary work via mono-OMOM deprotection, which had led to 39.2% [4].

#### 2.4.4. Advantage of Using the OMOM Protective Group: Selective Mono-Deprotection

As we have seen above, the OMOM protective group has another advantage in the synthesis of (di)methoxylated benzoxanthones, as we can consider mono-deprotection of the OMOM group in β-position to the carbonyl group using MgBr_2_·Et_2_O in benzene, and then performing cyclization (S_N_Ar) to form the desired benzoxanthone (Scheme 2). In this work, this method was applied to the synthesis of both SD-derived benzoxanthones (Scheme 3).

#### 2.4.5. Strategy Used for the Key S_N_Ar Reaction

Since the addition step is often the determining factor in reaction speed, the reactivity of S_N_Ar will depend on the size and electronegativity of the leaving group (-X) in the following order: F > MeO > Cl > Br > I. Even though -F is a better leaving group than -Cl, the latter has two major advantages: it is much easier to introduce and much less expensive. For these reasons, we finally decided to synthesize aldehydes, acid chlorides, and methyl esters bearing Cl as the leaving group.

Even though chlorine is less reactive than methoxy, it is possible to reverse it depending on the solvent used. This principle has been applied in a selective synthesis of the highly functionalized xanthone core of the antibiotic FD-594 [11]. Protic solvents solvate and stabilize phenate anions by slowing down the addition rate, which becomes the rate-determining step of the reaction. Therefore, the –MeO group becomes a better leaving group than –Cl in MeOH, H_2_O, formamide, or THF. On the contrary, the use of aprotic polar solvents enhances the nucleophilicity of the phenate anion and, as a result, the addition rate increases and the elimination step becomes the rate-determining step. Thus, –Cl, a better nucleophile than –MeO, becomes a better leaving group in DMF or DMPU.

### 2.5. Synthesis of the Schistodione A_3′,5′_-Derived Methoxylated Benzoxanthone ***5***

**Figure 1:**
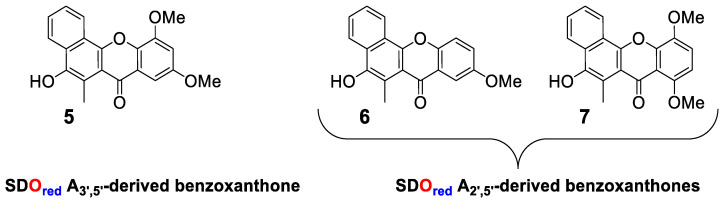
Potential methoxylated benzoxanthones **5**–**7** derived from schistodione oxides, under their reduced forms.

The methoxylated benzoxanthone **5** is the postulated metabolite derived from benzylMD, called schistodione A_3′,5′_ (Figure 1), generated via its metabolite schistodione oxide under its reduced form SDO_red_ A_3′,5′_.

The retrosynthetic route for the preparation of the dimethoxylated benzoxanthone **5** was designed in five steps (Scheme 3) from benzaldehyde **10** and the 2-bromo-3-methyl-diOMOM-naphthalene precursor **2b** described in Scheme 5 and Scheme 6.

**Scheme 8:**
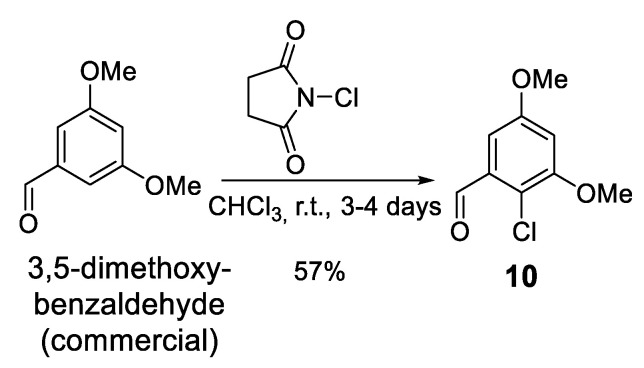
Synthesis of 2-chloro-3,5-dimethoxybenzaldehyde **10**.

The 2-chloro-3,5-dimethoxybenzaldehyde **10** was obtained in one step with a yield of 57% by electrophilic aromatic chlorination of commercial 3,5-dimethoxybenzaldehyde with NCS in chloroform (Scheme 8).

**Scheme 9:**
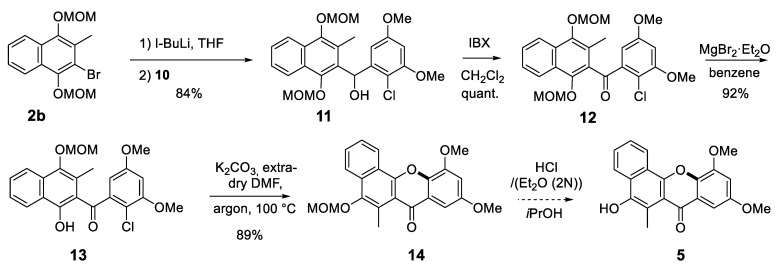
Synthesis of the dimethoxylated benzoxanthone **5** precursors.

Benzhydrol **11** is then synthesized by benzylic coupling between the lithium anion of **2b** and aldehyde **10** (Scheme 9). The halogen/metal exchange is carried out at −78 °C for 1 h in THF using 1.05 equiv. of *n*-BuLi. Next, a solution of chlorobenzaldehyde **10** is added dropwise at the same temperature. After 30 min, the reaction is processed to obtain **11** with 84% yield. Benzyl alcohol **11** is oxidized to **12** with IBX in CH_2_Cl_2_ with quantitative yield. Mono-deprotection of the OMOM group is carried out with MgBr_2_·Et_2_O in benzene to give the mono-deprotected product **13** with 92% isolated yield.

**Scheme 10:**
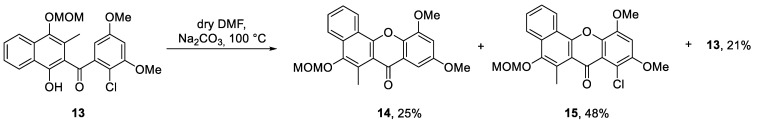
Formation of an oxidative coupling product during the S_N_Ar reaction.

In a preliminary attempt to perform the S_N_Ar from **13**, we had observed the formation of two S_N_Ar products, which were identified by NMR analysis, as the expected methoxylated benzoxanthone **14** and the chloro-benzoxanthone **15** (Scheme 10), isolated in a 1/1.92 ratio after purification.

Consequently, the S_N_Ar reaction to produce the protected benzoxanthone **14** was carried out with Na_2_CO_3_ in extra-dry DMF flushed with argon, at 100 °C. The deprotection of the OMOM protecting groups was attempted using HCl/Et_2_O (2 N) in iPrOH, following standard acidic conditions commonly employed for MOM-type ether cleavage. However, the target compound **5** exhibited significant instability and underwent rapid degradation. As a result, the deprotected product **5** could not be successfully isolated.

### 2.6. Synthesis of the Schistodione A_2′,5′_-Derived Monomethoxylated Benzoxanthone ***6***

As explained earlier, benzoxanthone **6** is the anticipated and postulated metabolite of schistodione A_2′,5′_ (Figure 1). The retrosynthetic scheme is described above (Scheme 3).

**Scheme 11:**
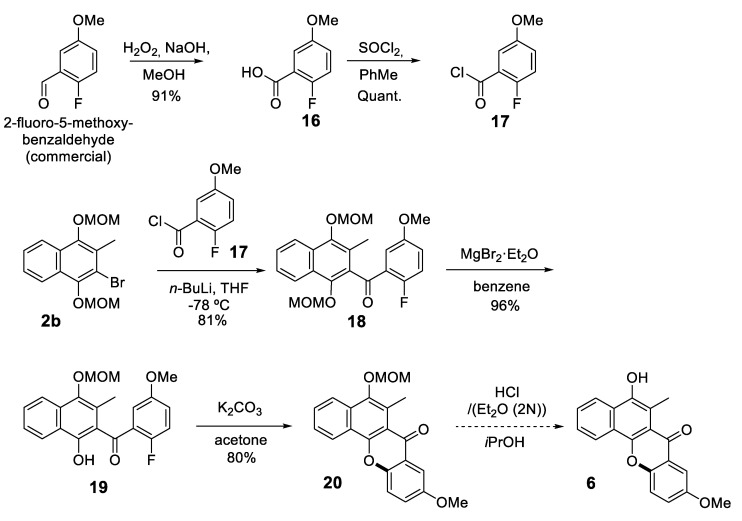
Synthesis of the monomethoxylated benzoxanthone **6** precursors.

First, commercial 2-fluoro-5-methoxybenzaldehyde is oxidized by hydrogen peroxide in a basic medium to give acid **16**, which is then converted to acid chloride by SOCl_2_ in toluene (Scheme 11). This 2-fluoro-5-methoxybenzoyl chloride **17** is used directly in the next step to prevent hydrolysis. This synthesis of acid chloride **17** is carried out in two steps with an overall yield of 91%.

Precursor **2b** is treated with 1.05 equiv. of *n*-BuLi in THF at −78 °C. One hour later, acid chloride **17** is added dropwise. After treatment and purification by silica column chromatography, benzophenone **18** is isolated with a yield of 81%. The monodeprotection of the OMOM group of **18** is carried out with MgBr_2_·Et_2_O in benzene at room temperature to give **19** with 96% yield (Scheme 11). This product undergoes intramolecular S_N_Ar by treatment with K_2_CO_3_ in acetone at 40 °C to give **20** with 80% yield. The OMOM protecting group was successfully cleaved using HCl/Et_2_O (2 N) in iPrOH. However, similarly to compound **5**, the resulting deprotected product **6** proved to be unstable and could not be isolated due to its rapid degradation.

### 2.7. Synthesis of the Schistodione A_2′,5′_-Derived Dimethoxylated Benzoxanthone ***7***

**Scheme 12:**
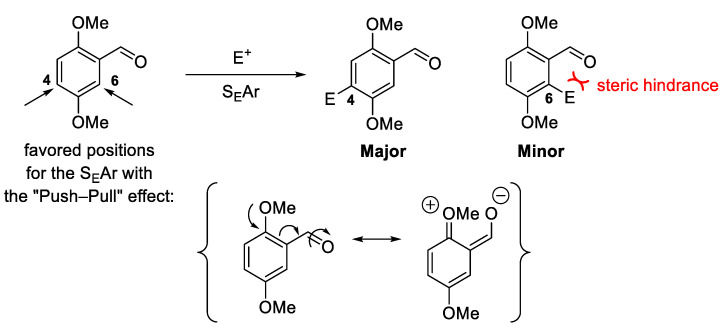
Reactivity scheme of the S_E_Ar leading to the favored four-substituted dimethoxy-benzaldehydes.

As shown in Scheme 12, during S_E_Ar (e.g., Cl_2_, Br_2_, etc.) on 2,5-dimethoxybenzaldehyde, position four is favored [12,13].

**Scheme 13:**
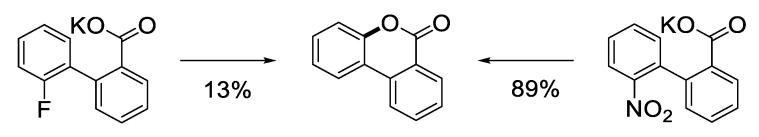
Compared reported reactivity of the S_N_Ar starting from 2′-nitro- versus 2′-fluoro-[1,1′-biphenyl]-2-carboxylates.

If one delves deeper into the literature on aromatic nucleophilic substitutions, we found that the NO_2_ group is also used as a nucleophile in S_N_Ar reactions and behaves in a manner described as “halogen-like” [14]. Its reactivity [15,16] varies according to F, NO_2_ >> Cl > Br > I or F > NO_2_ > Cl > Br > I depending on the different substrates, but it is generally described as a better leaving group than Cl, especially in intramolecular S_N_Ar reactions (Scheme 13). In this specific case, NO_2_ may even prove to be a better leaving group than fluorine [17]. Thus, given its halogen-like behavior, the reactivity of a nitro group as a leaving group increases with aprotic polar solvents, as we explained earlier in the case of chlorine.

In accordance with the synthesis strategy discussed above, the retrosynthetic scheme of the schistodione A_2′,5′_-derived dimethoxylated benzoxanthone **7** might start from the precursor 2-nitro-3,5-dimethoxybenzaldehyde **21**.

#### 2.7.1. Synthesis of 2-Nitro-3,5-dimethoxybenzaldehyde **21**

**Scheme 14:**
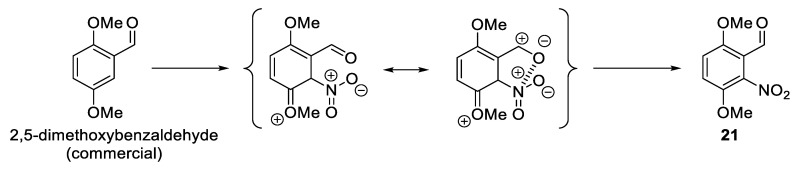
Nitration of 2,5-dimethoxybenzaldehyde.

Unlike electrophilic chlorination or bromination, the introduction of the nitro group at position six of 2,5-dimethoxybenzaldehyde is possible thanks to its electronic stabilization [18,19] at the *ortho* aldehyde position (Scheme 14).

**Scheme 15:**
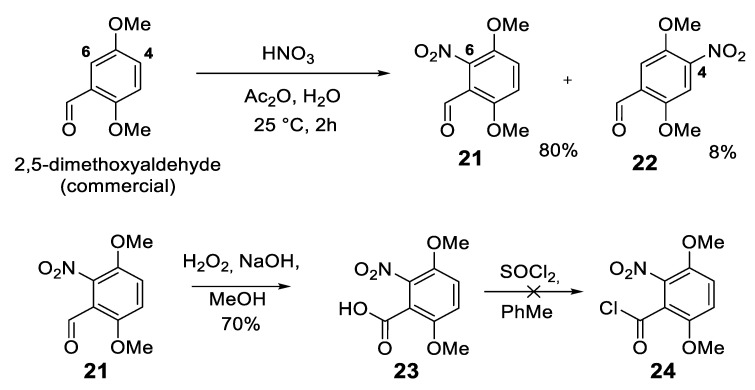
Synthesis of 2-nitro-3,5-dimethoxybenzaldehyde **21** and benzoic acid **23**.

2-Nitro-3,5-dimethoxybenzaldehyde **21** was synthesized by aromatic electrophilic nitration from commercial 2,5-dimethoxyaldehyde according to the described procedure [20] using nitric acid in acetic anhydride to give a mixture of *o*-nitration and *p*-nitration regioisomers **21** and **22**, respectively. We observed that when the reaction is carried out at 0 °C, the *p*-nitro/*o*-nitro ratio is 1:2. This ratio increases to 1:10 when the reaction is carried out at room temperature. Both regioisomers are easily separable by recrystallization in ethanol to obtain the desired aldehyde **21** with a yield of 72% (Scheme 15).

After separation, 2-nitro-3,5-dimethoxybenzaldehyde **21** was then oxidized to benzoic acid **23** by hydrogen peroxide in MeOH with 70% yield. When this acid was converted to acid chloride **24** by thionyl chloride, we only obtained degradation products (Scheme 15).

#### 2.7.2. Benzylic Coupling

**Scheme 16:**
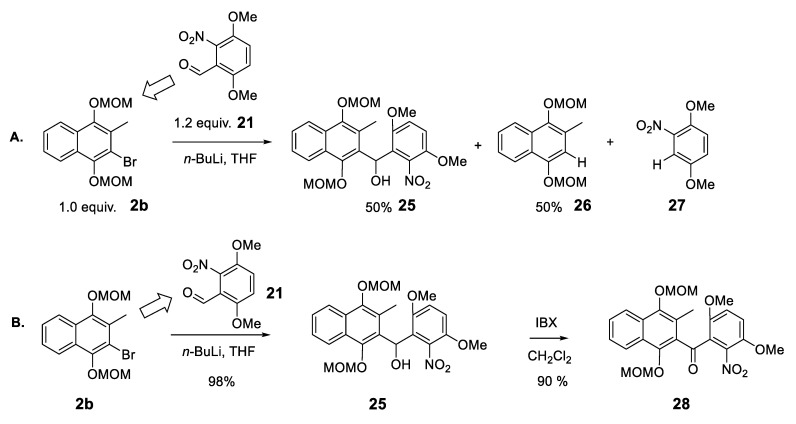
Optimized synthesis of the benzhydrol **25** from conditions (**A**) to conditions (**B**), e.g., by reverse addition of a preformed lithiated solution of **2b** to the nitrobenzaldehyde **21**, followed by its oxidation.

We used 2-nitro-3,5-dimethoxybenzaldehyde **21** to perform the benzylic coupling. Unlike in the previous case, the addition of nitrobenzaldehyde to a previously lithiated solution of **2b** yields only 50% of the coupling product **25**, accompanied by 50% of **26**, a product resulting from the debromination of **2b** (Scheme 16A). During column chromatography purification, another product was isolated and characterized: nitroarene **27**, corresponding to the deformylation product from starting material **21**. In order to increase the rate of the coupling reaction, we performed this reaction by reverse addition (Scheme 16B). This time, we obtained the coupling product **25** with an excellent yield (98% vs. 50%). The benzyl alcohol formed is then oxidized by IBX to give **28** with a yield of 90% (Scheme 16B).

The mono-OMOM-deprotection of **28** with MgBr_2_·Et_2_O in benzene gave naphthol **29**. Several cyclization conditions were tested (Table 1). When the product underwent S_N_Ar at room temperature for 24 h (entry 1), we obtained a 2:1 mixture of benzoxanthones **30** and **31**. When the reaction was carried out at T = 100 °C, we obtained a 4:1 mixture after 1 h of reaction (entry 3).

**Scheme 17:**
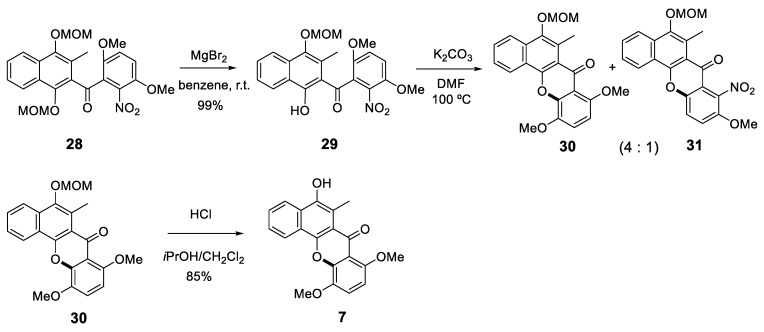
Synthesis of schistodione A_2′,5′_-derived dimethoxylated benzoxanthone **7**.

The mixture of benzoxanthones **30**:**31** was separated by silica column chromatography, and the OMOM group was deprotected with HCl in a mixture of iPrOH and CH_2_Cl_2_ to give the desired schistodione A_2′,5′_-derived dimethoxylated benzoxanthone **7** in 85% yield (Scheme 17). This synthesis was carried out in nine steps in 39% overall yield.

### 2.8. Synthesis of the Schistodione A_2′,5′_-Derived 2-((3,6-Dioxocyclohexa-1,4-dien-1-yl)methyl)-menadione

**Scheme 18:**
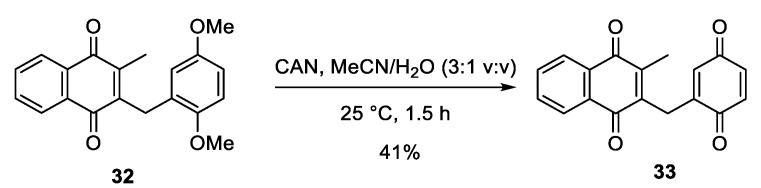
Oxidation of schistodione A_2′,5′_ **32** in the side chain to afford the potential metabolite **33**.

In addition, another unreported potential metabolite from schistodione A_2′,5′_ **32** was also considered, synthesized (Scheme 18), and used for investigations with *Sm*TGR as a potential target. To study the influence of a second quinone moiety in the antischistosomal lead compound **32** that could be generated via a possible bioactivation by oxidation of the side-chain in the parasite, the 2-[1′,4′-dimethoxy-]benzylMD **32** was oxidized by using three equiv. CAN as oxidant in a 1:1 mixture acetonitrile/water for 1.5 h at room temperature [2]. The desired product **33** could be obtained with 41% yield (Scheme 18).

### 2.9. Antiplasmodial and Antischistosomal Activities of Known Xanthones and Newly Synthesized Benzoxanthones

In a previous work, we also produced model xanthones for a physicochemical study [21] to evaluate their interactions with β-hematin and also in the redox-cycling assay based on methemoglobin and the human glutathione reductase to bioactivate the 3-benzoylMD metabolites. Various xanthones (Table 2) include the expected benzoxanthone **1** as the metabolite generated from plasmodione; the simple methoxylated xanthone **34**; the synthetic methoxylated benzoxanthone **35** substituted by a *p-*CF_3_ group as in plasmodione; the synthetic methoxylated benzoxanthone **36**, unsubstituted by a *p-*CF_3_ group. None of the model xanthones exerted efficiency to kill malarial parasites (IC_50_ > 3 µM), except the real plasmodione metabolite, the benzoxanthone **1**, substituted by a *p-*CF_3_ group. These assays against the chloroquine-sensitive *P. falciparum* strain 3D7 were performed in parallel with the parent 3-benzylMDs plasmodione and chloroquine, as controls.

The results indicate a preferential antiparasitic profile for the real plasmodione metabolite, the benzoxanthone **1**, rather than a nonspecific activity due to the xanthone core. Furthermore, to be active, the benzoxanthone **1** needs to be produced by drug bioactivation inside the plasmodial parasites.

Next, the same model xanthones (**1**, **34**, **35**, **36**) in Table 2 were also tested at 50 µM for their antischistosomal activity in vitro against adult *S. mansoni* worms for 48 h. Regardless of the xanthone tested, the treated worms (compared to the controls) were alive at each time point: 1 h, 2.5 h, 4 h, 5.5 h, 20 h, 24 h, and 48 h, confirming the absence of antischistosomal activity of (benzo)xanthones, when given extraneously.

With the benzoxanthone **1** produced in bulk, some of the 3-benzoylMDs (**4b**, **29**, **37** numbered as Cpnd 3c-MOM in ref. [3]) and benzoxanthones (**1**, **30**, **31, 38**) described in this work or previously, drug screening assays in vitro (Table 3) were carried out in parallel against a broad panel of various parasites in culture (*T. cruzi* amastigotes, *L. infantum* amastigotes, *T. brucei*, *P. falciparum* asexual stages), bacteria (*S. aureus*, *E. coli*) or fungi (*C. albicans*, *T. rubrum*, *A. fumigatus*). It is noteworthy to mention that the final benzoxanthones were very instable and insoluble in aqueous media as previously observed [3] at two pH values, supporting the fact that we only tested a few stable OMOM-protected benzoxanthones in drug screening assays. The cytotoxicity of the same compounds was evaluated against human fibroblasts (hMRC-5) and mouse macrophages (PMM). All assays were carried out with parent benzylMD (PD, SD-A_2′,5′_, SD-A_3′,5′_) and control drug references.

### 2.10. Structure–Activity Relationship (SAR) Analysis

The newly synthesized compounds based on the 3-benzoylMD scaffold (**4b**, **29**, **37**) exhibited higher antiparasitic activity than the corresponding benzoxanthone derivatives (**1**, **30**, **31**, **38**), which were largely inactive across all tested pathogens. The most apparently active compounds—the OMOM-partially deprotected 3-benzoylMD derivatives (e.g., **4b**, **37**)—displayed non-selective antikinetoplastidal activity against *T. cruzi* and *L. infantum* (IC_50_ values < 10 µM), at similar concentrations at which toxicity against hMRC-5 or PMM was expressed. Indeed, most compounds exhibited low or no activity (IC_50_ > 64 µM) against bacterial strains (*S. aureus*, *E. coli*) compared with reference drugs (erythromycin, chloramphenicol) and the fungus *A. fumigatus* (IC_50_ > 64 µM).

As plasmodione-based metabolites with the *p-*CF_3_ group were produced in bulk, we were able to test the whole series of synthetic representatives, i.e., from the benzoylMDs to the benzoxanthones. The presence of an aromatic CF_3_ substituent was associated with enhanced activity and the introduction of a fluorine atom further improved potency, as illustrated by a marked decrease in IC_50_ values against *T. cruzi* (**4b** vs. **37**: IC_50_ of 2.20 µM vs. 7.46 µM) or against the fungus *T. rubrum* (**4b** vs. **37**: IC_50_ of 1.40 µM vs. 2.35 µM). This is probably due to an increased lipophilicity and enhanced parasite cell penetration. Nitro-substituted and highly substituted derivatives (**29**, **31**) show poor or no activity, likely due to steric hindrance and/or reduced solubility. Compounds that are partially or fully OMOM-deprotected (e.g., the benzoxanthone **1**) showed markedly improved activity against *L. infantum* (IC_50_ = 2.03 µM). Overall, these results identify the benzoylMD core bearing CF_3_ or non-bulky electron-withdrawing substituents as the most promising framework for further antiparasitic optimization.

Partial OMOM deprotection resulted in improved activity against *T. cruzi*, although this effect was not systematic across all strains. Additionally, compounds with moderate to good solubility (h, m) generally displayed higher biological activity, whereas poorly soluble derivatives (p) were inactive, highlighting solubility as a key determinant of efficacy. Overall, an optimal balance between lipophilicity and polarity is critical for activity.

### 2.11. Study of Antischistosomal benzylMDs Mode of Action

**Figure 2:**
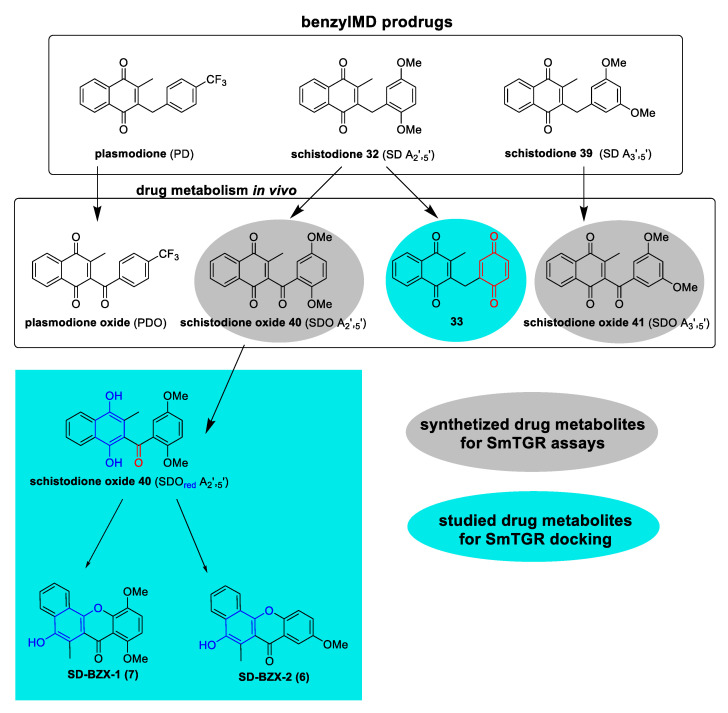
Structures of the synthesized metabolites **33**, **40,** and **41** potentially generated from antischistosomal benzylMD **32** and **39** investigated in mode of action studies, in recombinant *Sm*TGR enzymic assays (using **40_ox_**, **41_ox_** as substrates), or in docking studies with *Sm*TGR “doorstop pocket” structure (using **40_ox_**, **40_red_**, schistodione-based benzoxanthones SDO-BZX A_3′,5′_ **5**, SDO-BZX A_2′,5′_-**6** and **7**, and the bis(quinone) **33**, as ligands).

Based on our previous work [4] with plasmodione and its metabolites (e.g., benzyl oxidation to generate a highly active metabolite called plasmodione oxide (PDO) with a 3-benzoylMD skeleton), we propose that the biological activity of antischistosomal 3-benzylMD is mediated via the active benzoylMD metabolites following benzylic oxidation in vivo. The potential benzoylMD metabolites **40_ox_** and **41_ox_** (Figure 2), respectively, generated from benzylMD **32** and **39**, were produced, according to reported procedures [23], as pure product references for investigations on the recombinant drug target *Sm*TGR.

### 2.12. A Possible Role for S. mansoni Thioredoxin-Glutathione Reductase

We previously reported that, in plasmodione-treated *P. falciparum*-parasitized red blood cells, both human and *P. falciparum* glutathione reductases (GR) might play a role and contribute to the plasmodione metabolites-mediated redox-cycling process [24]. We showed that purified human GR (hGR) could catalyze redox cycling using benzoylMD as substrate [24]. Moreover, in vitro experiments using purified hGR showed that a PD-derived photoaffinity labeling probe could bind to a cysteine, Cys234, of that enzyme [25]. Cys234 is located in proximity of the FAD domain of GR catalytic center in the so-called “doorstop pocket” [26]. This cavity was first reported in the *S. mansoni* TGR (*Sm*TGR) [27].

In the *Sm*TGR assay using 3 mM DTNB as sulfide substrate, IC_50_ values were measured in 0.1 M phosphate buffer, 10 mM EDTA at 25° C, in the presence of 100 μM NADPH, 10 nM *Sm*TGR, and 50 μM compound. As expected, both lead benzylMD prodrugs displayed moderate inhibition (~36% for **32**, ~34% for **39** confirming that the benzylMD prodrugs *per se* act as poor *Sm*TGR inhibitors.

**Figure 3:**
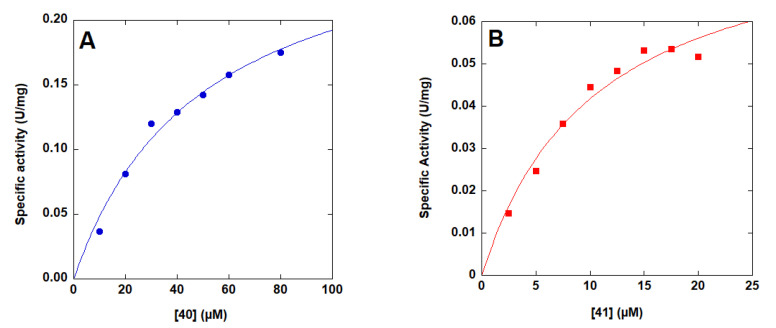
3-BenzoylMD reductase activity of *Sm*TGR. (**A**) The naphthoquinone reductase activity of *Sm*TGR was tested by monitoring the oxidation of NADPH at 340 nm, 25 °C, and pH 7.4 under steady-state conditions. Both 3-BenzoylMDs **40_ox_** ((**A**), blue curve) and **41_ox_** ((**B**), red curve) were dissolved in DMSO, and the NADPH oxidation activity was measured at different concentrations in duplicate (0–80 µM) in the presence of 2% DMSO. For the determination of *K*_m_ and *V*_max_ values, the steady-state rates were fitted by using nonlinear regression analysis software (Kaleidagraph, version 3.6.) to the Michaelis–Menten equation [29], and the turnover number *k*_cat_ and the catalytic efficiency *k*_cat_/*K*_m_ were calculated. The initial rate for aerobic NADPH oxidation activity of *Sm*TGR was not subtracted from the rates measured in the presence of the naphthoquinone because it proved negligible in comparison to that of *Sm*TGR-catalysed benzoylMD reductase activity.

*Sm*TGR, the solely pyridine nucleotide-disulfide oxidoreductase able to reduce both thioredoxin and glutathione disulfides in parasitic platyhelminths [28], could be a good candidate to play a role in benzoylMD reduction and redox cycling. First, we observed that purified recombinant *Sm*TGR could redox cycle with benzoylMD metabolites **40**–**41** of antischistosomal benzylMDs (Figure 2). We found that both benzoylMD, **40** and **41** (Figure 3), were efficient subversive substrates of *Sm*TGR with *K*_m_ = 49.68 µM and *k*_cat_ = 0.33 s^−1^ for **40** (Figure 3A) and *K*_m_ = 10.47 µM and *k*_cat_ = 0.1 s^−1^ for **41** (Figure 3B). Those compounds have a catalytic efficiency *k*_cat_/*K*_m_ of 6.6 mM^−1^ s^−1^ for **40** and 9.6 mM^−1^ s^−1^ for **41** similar to the one obtained for previously tested 3-benzoylMDs, such as PDO in the GR assay [24].

Furthermore, with a second quinone moiety in the benzyl chain, the **32**-generated bis(quinone) metabolite **33** becomes more oxidant and strongly inhibits *Sm*TGR with an apparent IC_50_ value of 158 nM, likely by irreversible inhibition due to possible Michael addition with a protein nucleophile.

**Figure 4:**
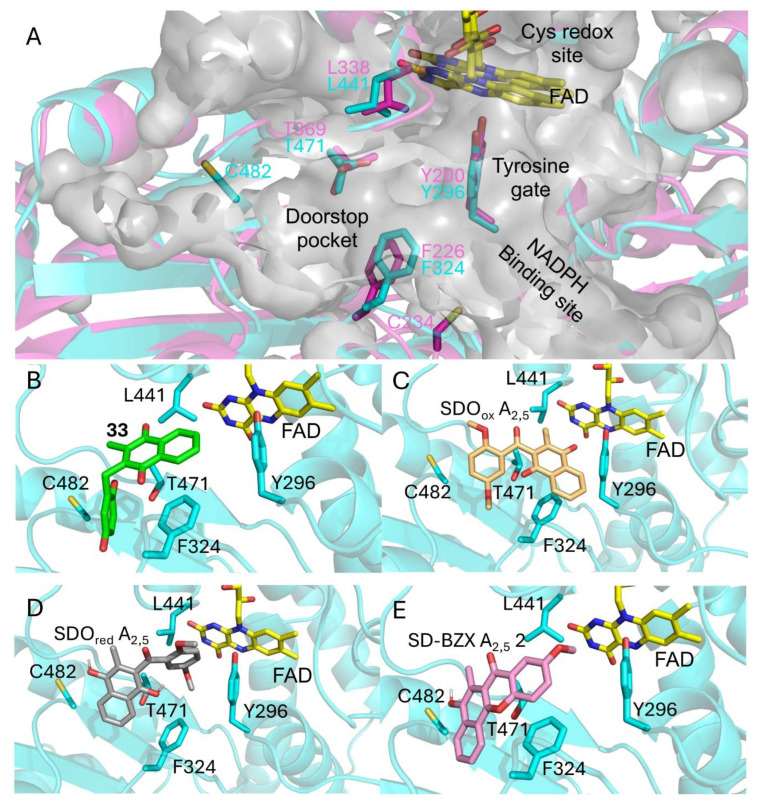
Crystal structure of human glutathione reductase (hGR, PDB ID: 1GRA, magenta ribbons) superimposed on *Schistosoma mansoni* thioredoxin glutathione reductase (*Sm*TGR, PDB ID: 2X8G, cyan ribbons) (**A**). The “doorstop pocket” is highlighted in relation to the conserved tyrosine gate and the NADPH-binding site. The NADPH binding site and the “doorstop pocket” are represented as solvent-exposed surfaces. Key cysteine residues, Cys482 in *Sm*TGR and Cys234 in hGR, the latter identified by PD-derived photoaffinity labeling (see text), are shown as sticks, together with some crucial residues shaping the pocket [27]. (**B**–**E**) show the docking poses, obtained with the HADDOCK (https://rascar.science.uu.nl/haddock2.4/, accessed on 19 August 2025), of compound **33** (in green sticks), oxidized schistodione **40** (SDO_ox_ A_2′,5′_, in orange sticks), reduced schistodione **40** (SDO_red_ A_2′,5′_, in grey sticks), and schistodione benzoxanthone-2 **6** (SDO-BZX A_2′,5′_, in pink sticks), respectively. In all cases, the ligands localize between Cys482 and Y296, the tyrosine gate, with key pocket residues and FAD highlighted.

Second, docking simulations showed that benzylMDs could bind to *Sm*TGR in the doorstop pocket (Figure 4) [27]. As mentioned above, we showed that a PD-derived photoaffinity labeling probe could bind in the same pocket located on the *re-*face of the FAD in hGR and far from the redox active cysteines located at the *si*-face of the cofactor (Figure 4A) [25]. This pocket is conserved across all members of the pyridine nucleotide–disulfide oxidoreductase family, including high-molecular-weight TrxRs and TGRs. Recent studies, particularly those on *Sm*TGR, have identified this site as a druggable target with the identification of non-covalent *Sm*TGR inhibitors with schistosomicidal activity in vivo [27,30]. This pocket has been named the “doorstop pocket”, as compounds binding there can hinder the out conformation of the tyrosine gate, which is essential for NADPH binding and FAD reduction, while simultaneously interfering with NADP^+^ release during turnover (Figure 4A). All previously identified compounds targeting this pocket in TGR/TrxRs are non-covalent [26,31]. Therefore, benzylMDs represent the first proof of principle that this allosteric site can be targeted by selective reactive compounds, expanding the range of strategies available to modulate this fundamental allosteric pocket involved in the function of this protein family.

Cys234, which is selectively alkylated in hGR [25], is buried in the crystal structure of the oxidized enzyme [32] and positioned on the floor of the pocket (Figure 4A). However, it is likely to be transiently exposed during enzyme turnover. Indeed, the plasticity of the doorstop pocket is established, as it becomes more mobile upon enzyme reduction [31]. This structural flexibility suggests that Cys234 can be momentarily exposed, allowing for alkylation. While *Sm*TGR lacks a cysteine at the same position as in hGR, it possesses another cysteine within the doorstop pocket (Cys482, Figure 4A). We therefore propose that the cysteine in the doorstop pocket of *Sm*TGR, as that of hGR, can be targeted by benzylMDs. Supporting this hypothesis, docking simulations indicate that the putative **32**-generated bis(quinone) metabolite **33** binds within the doorstop pocket, positioning its reactive moiety near Cys482 (Figure 4B). This arrangement is particularly intriguing, as it suggests the possibility of a Michael addition to Cys482 in *Sm*TGR. The molecule established multiple hydrophobic contacts with Y296, F324, P439, L441, V469, and A481, along with a hydrogen bond with T471, resulting in a predicted interaction free energy (ΔG) of −7.9 kcal/mol.

Docking calculations were also performed with schistodione oxide **40**, in both its oxidized (SDO_ox_ A_2,5_) and reduced (SDO_red_ A_2,5_) states, as well as with schistodione-generated benzoxanthones-**6** and -**7**—see structures generated from SDO-BZX A_2′,5′_ in Figure 2. All docking poses yielded predicted binding energies around −8 kcal/mol (Figure 4C–E and Figure S1 in the Supplementary Materials). Interestingly, the redox-active moieties of the docked compounds, such as quinone, menadione, or methylnaphthol, are located within 8 Å of the closest atom of FAD, suggesting that electron transfer from the reduced cofactor to the benz(o)ylMD derivatives could indeed take place [28,33]. In the oxidized form of schistodione oxide **40**, the menadione moiety is located near the flavin, poised to accept electrons from the NADPH-reduced flavin. In the reduced form, the menadiol core rotates to the opposite side relative to Cys482, placing it in a position favorable for alkylation by this residue. For schistodione-generated benzoxanthone-**7**, the methyl-naphthol group is oriented close to Cys482, suggesting again potential alkylation of the quinone methide—upon deprotonation of the phenol part—by the thiol group. By contrast, benzoxanthone-**6** is located at a greater distance from Cys482 just upon Y296, the tyrosine gate (Figure S1 in the Supplementary Materials). This, along with evidence that benzoylMD probes bind to Cys234 in hGR [25], suggests that the doorstop pocket may represent the previously unrecognized site within the *N*-terminal domain of pyridine nucleotide-disulfide oxidoreductases, where the reduction of specific quinones, such as juglone, takes place [34].

## 3. Discussion

During the synthesis of precursors of schistodione A_2′,5′_- and A_3′,5′_-derived methoxylated benzoxanthones **5**–**7**, we encountered very unexpected reactions that are worth discussing.

**Scheme 19:**
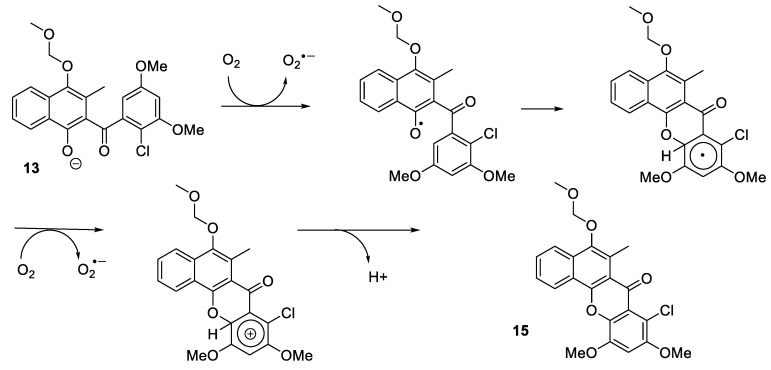
Mechanism of the oxidative phenolic coupling occurring during the S_N_Ar reaction.

First, it was interesting to observe an oxidative phenolic coupling product during the S_N_Ar reaction in the synthesis of the dimethoxylated benzoxanthone **5**. Indeed, during the cyclization process, dry DMF under argon had not been intensively degassed, so it is highly likely that dissolved oxygen was present allowing an oxidative phenolic coupling to occur (Scheme 19). The mechanism is described below.

Secondly, we observed an unexpected deformylation product during the S_N_Ar in the synthesis of the methoxylated benzoxanthone **7**. In order to provide an acceptable explanation for the observed secondary deformylation reaction (Scheme 16A), we conducted extensive research in the literature to fully understand the characteristics and behavior of nitroarenes in the presence of radicals.

### Assumed Deformylation Mechanism Based on the Electrochemical Properties of Nitroarenes

**Scheme 20:**
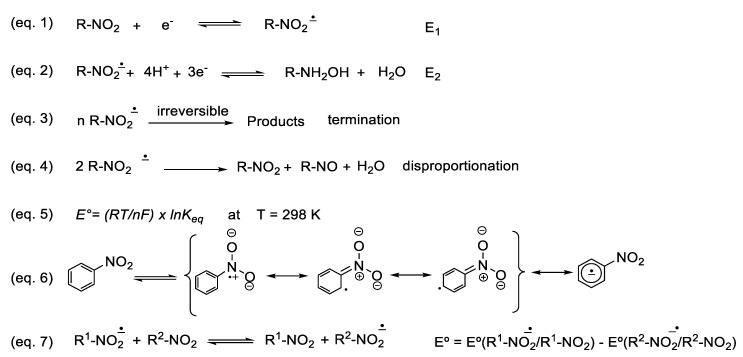
Bioactivation of nitro groups by starting bioreductive radical reactions.

One of the properties of nitroaromatics is that they can fairly easily accept an electron to form an anion radical. It is worth noting that a number of clinical drugs contain nitro groups, which undergo radical metabolism when introduced in vivo (Scheme 20) [35]. The first step E1 (Equation (1), Scheme 20) in this bioreductive process of nitro group is defined by the equation involving the redox pair (“nitro”/carbanion radical), whose E_1/2_ values provide an appropriate method for defining its reactivity. The second step E2 (Equation (2)) is the reduction of the nitroxyl radical to hydroxylamine [36]. Reduction E1 is favored in aprotic or mixed (aprotic/protic) media, at pH > 8 [36] and at low reagent concentrations. The E1/E2 ratio defines the stability of the anion radical, which can undergo isomerization or dismutation reactions (Equations (3) and (4), Scheme 20) [37].

These reactions are in equilibrium, governed by Nernst’s equation (Equation (5), Scheme 20), from which the equilibrium constant (*K*_eq_) can be calculated from the oxidation-reduction potentials.

**Scheme 21:**
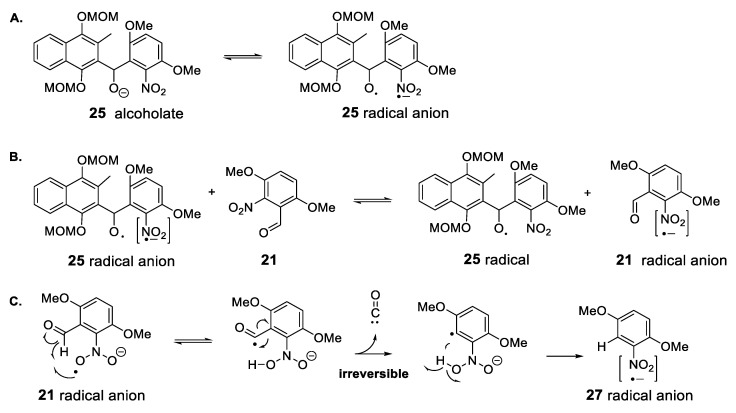
Putative mechanism of deformylation of α-nitrobenzaldehyde **21** radical anion, from nitro group reduction (**step A**), then “alcoholate—nitroarene” electron transfer (**step B**), and α-nitrobenzaldehyde radical anion transposition (**step C**), leading to the generation of the observed deformylated product **27** as radical anion.

Electro-attracting groups in *ortho* and *para* positions to the nitro group stabilize the nitroxyl radical, as shown in Scheme 20 (Equation (6)) [38]. The nitro group is known to undergo redox reactions with alcoholates [39]. In the present case, when the benzyl coupling product **25** is formed, the nitro group could react with the formed alcoholate to give the radical anion **25** (Scheme 21A) [40].

Then, it is assumed that an “alcoholate—nitroarene” electron transfer occurred between the radical anion **25** and the 2-nitro-3,5-dimethoxybenzaldehyde **21** (Scheme 21B). This type of electron transfer between alcoholates and nitroarenes is described in literature by Shifman et al. [41] and mechanistic reaction studies had been investigated [42]. Additionally, nitroxybenzyl radical anions are known to undergo electron transfer between them during redox reactions (Equation (7), Scheme 20) [43]. In this way, the nitrosylbenzhydrol diradical **25** could be oxidized to the alcoholate radical **25** by transferring an electron to the nitro group of the 2-nitro-3,5-dimethoxybenzaldehyde **21**. The latter is thus reduced to the nitro radical anion **21** (Scheme 21B).

It is well documented that several photochemistry and UV-Vis ESR studies describe *o*-nitrobenzaldehydes as substrates of deformylation reactions by photolysis via a formyl radical [44]. We therefore applied these photochemical concepts to our specific case and assumed that the α-nitrobenzaldehyde radical anion **21** undergoes deformylation according to the mechanism shown in Scheme 21C. Thus, the decarbonylation led to the generation of the observed deformylated **27** radical anion. The deformylation reaction, shown in Scheme 16A, would be the only irreversible step in this redox reaction cascade and also the driving force behind the reaction.

Regarding the antiparasitic activities of methoxylated schistodione metabolites, the absence of toxicity following external administration of drug metabolites in vitro in parasite cultures reflects the necessity for site-specific metabolic activation of 3-benzylMDs in the parasites, whereby reactive intermediates exert toxic effects only when generated in close proximity to their cellular targets. These results indicate that, to be active the benzoxanthones need to be produced by drug bioactivation inside the parasites. Furthermore, as expected for plasmodione metabolites, a preferential antiplasmodial profile rather than antibacterial or antifungal activity was observed. For this reason, methoxylated benzoxanthones as drug metabolites of antischistosomal schistodiones represent a very limited environmental risk in accordance with the biological data presented here.

## 4. Materials and Methods

### 4.1. Chemistry: General

All the reagents and solvents were purchased from commercial sources and used as received, unless otherwise stated. The ^1^H, ^19^F {^1^H} and, and ^13^C {^1^H} NMR spectra were obtained in CDCl_3_, Acetone-*d*_6_ as solvents using 300 MHz, 400 MHz or 500 MHz Bruker Avance spectrometers (Bruker Daltonics, Bremen, Germany). Chemical shifts were reported in parts per million (δ). ^1^H NMR data were reported as follows: chemical shift (δ ppm) (multiplicity, coupling constant (Hz), and integration). Multiplicities are reported as follows: s = singlet, d = doublet, t = triplet, q = quartet, m = multiplet, or combinations thereof. High-resolution mass spectroscopy (HRMS) spectra were recorded using the electron spray ionization (ESI) technique using a microTOF LC spectrometer (Bruker Daltonics, Bremen, Germany). Reactants were purchased from commercial sources, such as Fluorochem (Hadfield, Derbyshire, UK), Sigma-Aldrich (Saint Quentin Fallavier, France), BLDpharm (Reinbek, Germany), and Alfa Aesar (Karlsruhe, Germany).

### 4.2. General Procedure of Precursors

*2-Bromo-3-methyl-1,4-dihydronaphthalene-1,4-dione* (**8**). Menadione (1.0 equiv., 25.0 g, 145 mmol) was dissolved in dichloromethane (100 mL), and a solution of bromine (1.05 equiv., 24.4 g, 7.83 mL, 152 mmol) in dichloromethane (20 mL) was added dropwise over 30 min at room temperature. The reaction mixture was stirred for an additional 1 h, after which TLC analysis indicated complete formation of the dibrominated intermediate. Pyridine (1.05 equiv., 12.1 g, 12.3 mL, 152 mmol) was then added, and the reaction mixture was stirred overnight at room temperature. The reaction mixture was poured into water, and the layers were separated. The organic layer was washed with an aqueous solution of Na_2_S_2_O_3_, then with brine, dried over anhydrous MgSO_4_, filtered, and concentrated under reduced pressure to afford a brown solid. The crude product was purified by crystallization from methanol to afford compound **8** as red needles (33.98 g, 135.3 mmol, 93% yield). ^1^H NMR (400 MHz, CDCl_3_) δ 7.51 (dd, *J* = 7.5, 2.7 Hz, 2H), 6.80 (dq, *J* = 7.5, 2.7 Hz, 2H), 2.39 (s, 3H). ^13^C {^1^H} (101 MHz, CDCl_3_) δ 182.1, 177.6, 148.6, 139.2, 134.2, 134.0, 131.7, 131.3, 127.6, 127.2, 77.5, 77.2, 76.8, 18.0.

*2-Bromo-1,4-bis(methoxymethoxy)-3-methylnaphthalene* (**2b**). Water (750 mL) and dichloromethane (750 mL) were combined with Aliquat 336 (0.31 equiv., 4.99 g, 5.67 mL, 12.3 mmol), and the biphasic mixture was degassed under an argon atmosphere for 1 h. 2-Bromo-3-methyl-1,4-dihydronaphthalene-1,4-dione (**8**) (1.0 equiv., 10.0 g, 39.8 mmol) and sodium dithionite (4.0 equiv., 27.7 g, 159 mmol) were added, and the reaction mixture was stirred for 1 h at room temperature. Degassed aqueous NaOH (3 M, 5.3 equiv., 70.4 mL, 211 mmol) was added dropwise at 0 °C; after 10 min, chloromethyl methyl ether (MOMCl, 3.0 equiv., 9.62 g, 9.08 mL, 119 mmol) was added and the reaction mixture was stirred for 1 h. A second portion of degassed aqueous NaOH (3 M, 70 mL) was added, followed after 10 min by an additional portion of MOMCl (9.1 mL), and the reaction mixture was stirred overnight at room temperature. The reaction was quenched by the addition of solid NaOH (6.0 g). The phases were separated, and the aqueous phase was extracted with dichloromethane (2 × 200 mL). The combined organic layers were washed with brine (200 mL), dried over anhydrous Na_2_SO_4_, filtered, and concentrated under reduced pressure. The crude product was purified by crystallization from diethyl ether (70 mL) to afford the desired compound **2b** as a beige powder (7.0 g, 51.5% yield). ^1^H NMR (300 MHz, CDCl_3_) δ 8.14–8.17 (m, 1H), 8.05–8.08 (m, 1H), 7.48–7.57 (m, 2H), 5.24 (s, 3H), 5.12 (s, 3H), 3.75 (s, 3H), 3.69 (s, 3H), 2.58 (s, 3H). ^13^C {^1^H} (75 MHz, CDCl_3_) δ 148.2, 147.7, 128.2, 128.1, 127.7, 126.6, 126.2, 122.6, 122.3, 117.5, 100.3, 100.2, 58.3, 58.0, 17.8. EI MS (70 eV, *m*/*z* (%)): 215.1 (100), 340.0 ([M]^+^, 51), 185.1 (49), 115.0 (46), 261.1 (45), 171.0 (38), 143.0 (29).

*2-Chloro-3,5-dimethoxybenzaldehyde* (**10**). A solution of NCS (1.6 equiv., 4.5 g, 33.7 mmol) dissolved in CHCl_3_ (30 mL) was added gradually to a mixture of 3,5-dimethoxybenzaldehyde (1.0 equiv., 3.5 g, 21.062 mmol) in CHCl_3_ (15 mL). The addition was endothermic. The mixture was stirred at for 24 h at 25 °C then 24 h at reflux. After the mixture was cooled, dichloromethane (50 mL) was added. The solution was washed by saturated sodium thiosulfate solution (2 × 35 mL), and then with water (2 × 35 mL). The organic phase was dried over MgSO_4_, filtered then concentrated. The crude residue was purified by flash chromatography on silica gel (Cyclohexane/Diethyl ether/Dichloromethane 3/1/0.4) to afford 2-chloro-3,5-dimethoxybenzaldehyde (2.42 g, 12.063 mmol, 57.27%) as a white solid. ^1^H NMR (300 MHz, CDCl_3_) δ 10.49 (s, 1H), 7.00 (d, *J* = 2.9 Hz, 1H), 6.72 (d, *J* = 2.9 Hz, 1H), 3.91 (s, 3H), 3.84 (s, 3H). ^13^C NMR {^1^H} (75 MHz, CDCl3) δ 190.0, 159.2, 156.5, 133.7, 119.8, 106.2, 102.6, 56.7, 55.9.

*[1,4-Bis(methoxymethoxy)-3-methylnaphthalen-2-yl](2-chloro-3,5-dimethoxyphenyl)methanol* (**11**). 2-Bromo-1,4-bis(methoxymethoxy)-3-methylnaphthalene (**8**) (1.2 equiv., 975 mg, 2.86 mmol) was dissolved in dry THF (4.9 mL) under an argon atmosphere, and the solution was cooled to −78 °C. *n*-Butyllithium (1.6 equiv., 1.6 M in hexanes, 2.38 mL, 3.81 mmol) was added dropwise, and the reaction mixture was stirred for 45 min at −78 °C. The resulting organolithium solution was then added dropwise to a solution of 2-chloro-3,5-dimethoxybenzaldehyde (**10**) (1.0 equiv., 498 mg, 2.38 mmol) in THF at −78 °C, and the reaction mixture was stirred for 1.5 h at this temperature. The reaction was quenched by pouring the mixture into saturated aqueous NH_4_Cl. The phases were separated, and the aqueous phase was extracted with diethyl ether. The combined organic layers were washed with saturated aqueous NaHCO_3_, then with saturated aqueous NaCl, dried over anhydrous MgSO_4_, filtered, and concentrated under reduced pressure. The crude product was purified by silica gel column chromatography (cyclohexane/ethyl acetate, 4:1) to afford the desired compound **11** as a yellow oil (1.13 g, 85% yield). m.p. 46–47 °C. ^1^H NMR (300 MHz, CDCl_3_) δ 8.11 (dd, *J* = 8.0, 1.8 Hz, 1H), 7.97 (dd, *J* = 8.0, 1.8 Hz, 1H), 7.46–7.54 (m, 2H), 7.03 (dd, *J* = 2.8, 0.8 Hz, 1H), 6.57 (d, *J* = 5.5 Hz, 1H), 6.47 (d, *J* = 2.8 Hz, 1H), 5.06–5.08 (m, 2H), 3.84 (s, 3H), 3.83 (s, 3H), 3.63 (s, 3H), 3.59 (s, 3H), 2.34 (s, 3H). ^13^C NMR {^1^H} (75 MHz, CDCl_3_) δ 158.5, 155.7, 148.5, 148.4, 142.9, 131.6, 129.3, 127.0, 126.8, 126.5, 125.7, 122.6, 122.5, 112.4, 105.2, 100.2, 100.1, 98.5, 68.5, 57.9, 57.6, 56.2, 55.5, 13.9. EI-MS (70 eV) *m*/*z* (%) 365.1 (100), 340.0 (26), 462.1 ([M]^+^, 4).

*1,4-Bis(methoxymethoxy)-3-methylnaphthalen-2-yl)(2-chloro-3,5-dimethoxyphenyl)methanone* (**12**). 1,4-Bis(methoxymethoxy)-3-methylnaphthalen-2-yl (**11**) (1.0 equiv., 1.00 g, 2.16 mmol) was dissolved in dichloromethane (23 mL) under an argon atmosphere at room temperature. IBX (3.0 equiv., 1.81 g, 6.48 mmol) was dissolved in DMSO (25.6 mL) and added dropwise to the reaction mixture, which was then stirred for 4 h until complete conversion. Dichloromethane was added, and the reaction mixture was washed with an aqueous solution of Na_2_S_2_O_3_, and then with water. The phases were separated, and the organic layer was washed with brine, dried over anhydrous Na_2_SO_4_, filtered, and concentrated under reduced pressure. The crude product was purified by silica gel column chromatography (cyclohexane/ethyl acetate, 3:1) to afford the desired compound **12** as a yellow oil (990 mg, 99.4% yield). ^1^H NMR (300 MHz, CDCl_3_) *δ* 8.14 (ddd, *J* = 8.1 Hz, *J* = 4.2 Hz, *J* = 0.9 Hz, 2H), 7.49–7.62 (m, 2H), 6.67 (d, *J* = 2.8 Hz, 1H), 6.58 (d, *J* = 2.8 Hz, 1H), 5.14 (s, 2H), 4.98 (s, 2H), 3.90 (s, 3H), 3.74 (s, 3H), 3.68 (s, 3H), 3.46 (s, 3H), 2.36 (s, 3H). ^13^C NMR {^1^H} (75 MHz, CDCl_3_) *δ* 195.8, 158.6, 156.5, 148.3, 147.7, 139.2, 132.1, 127.6, 127.5, 126.1, 125.7, 124.6, 123.3, 122.9, 114.0, 107.1, 103.1, 101.4, 100.2, 57.9, 57.8, 56.5, 55.7, 13.9. EI MS (70 eV, *m*/*z* (%)): 383.1 (100), 393.1 (47), 83.9 (46), 335.1 (29), 460.1 ([M]^+^, 6).

*2-[(2-Chloro-3,5-dimethoxyphenyl)carbonyl]-4-(methoxymethoxy)-3-methylnaphthalen-1-ol* (**13**). 1,4-Bis(methoxymethoxy)-3-methylnaphthalen-2-yl (**12**) (1.0 equiv., 900 mg, 1.95 mmol) was dissolved in benzene (15 mL) under an argon atmosphere. Dibromomagnesium·ethoxyethane (1.3 equiv., 655 mg, 2.54 mmol) was added, and the reaction mixture was stirred for 1 h at 25 °C. The reaction was quenched by pouring the mixture into saturated aqueous NH_4_Cl. The phases were separated, and the aqueous phase was extracted with diethyl ether. The combined organic layers were washed with brine, dried over anhydrous Na_2_SO_4_, filtered, and concentrated under reduced pressure. The crude product was purified to afford the desired compound **13** as a bright yellow solid (750 mg, 92.1% yield). ^1^H NMR (300 MHz, CDCl_3_) δ 13.63 (s, 1H), 8.51 (d, *J* = 8.4 Hz, 1H), 8.04 (d, *J* = 8.4 Hz, 1H), 7.67–7.73 (m, 1H), 7.51–7.57 (m, 1H), 6.62 (d, *J* = 2.7 Hz, 1H), 6.44 (d, *J* = 2.7 Hz, 1H), 5.03 (s, 2H), 3.93 (s, 3H), 3.78 (s, 3H), 3.62 (s, 3H), 2.02 (s, 3H). ^13^C NMR {^1^H} (75 MHz, CDCl_3_) δ 199.8, 161.3, 159.6, 144.3, 142.8, 132.9, 131.0, 126.0, 125.1, 124.4, 122.2, 115.3, 111.5, 103.9, 101.6, 100.3, 96.1, 58.1, 56.6, 56.0, 15.8. EI-MS (70 eV) *m*/*z* (%) 356.1 (100), 341.1 (41), 358.1 (36), 371.1 (29), 171.0 (27), 115.0 (26), 416.1 ([M]^+^, 21).

*2,4-Dimethoxy-10-(methoxymethoxy)-11-methyl-12H-5-oxatetraphen-12-one* (**14**). 12-[(2-Chloro-3,5-dimethoxyphenyl)carbonyl]-4-(methoxymethoxy)-3-methylnaphthalen-1-ol (**13**) (1.0 equiv., 650 mg, 1.56 mmol) was dissolved in DMF (71.4 mL) under an argon atmosphere, and K_2_CO_3_ (1.5 equiv., 323 mg, 2.34 mmol) was added. The reaction mixture was heated at 100 °C for 1 h. After cooling to room temperature, the mixture was poured into saturated aqueous NH_4_Cl (40 mL), and water was added to induce precipitation. The resulting solid was collected by filtration, dissolved in ethyl acetate, dried over anhydrous Na_2_SO_4_, filtered, and concentrated under reduced pressure. The crude product was purified by silica gel column chromatography using toluene/diethyl ether (4:1) as eluent to afford the desired compound 14 as a white solid (527 mg, 89% yield). ^1^H NMR (300 MHz, CDCl_3_) δ 8.72 (d, *J* = 8.5 Hz, 1H), 8.19 (d, *J* = 8.5 Hz, 1H), 7.72–7.77 (m, 1H), 7.63–7.68 (m, 1H), 7.27 (d, *J* = 2.8 Hz, 1H), 6.76 (d, *J* = 2.8 Hz, 1H), 5.16 (s, 2H), 4.05 (s, 3H), 3.92 (s, 3H), 3.72 (s, 3H), 2.99 (s, 3H). ^13^C NMR {^1^H} (75 MHz, CDCl_3_) δ 178.4, 156.4, 152.0, 149.9, 147.4, 140.9, 131.5, 130.0, 126.5, 126.1, 124.2, 123.6, 122.5, 116.7, 105.7, 100.3, 96.1, 58.2, 56.6, 55.9, 15.5. EI-MS (70 eV) *m*/*z* (%) 335.1 (100), 380.1 ([M]^+^, 30).

*2-Fluoro-5-methoxybenzoic acid* (**16**). A solution of NaOH (4.4 equiv., 2.28 g, 57.091 mmol) in H_2_O (4 mL) was added to a suspension of 2-fluoro-5-methoxybenzaldehyde (1.0 equiv., 2 g, 1.62 mL, 12.98 mmol) in MeOH (22 mL). The mixture was stirred at 65 °C. Aqueous H_2_O_2_ (30% *w*/*v*, 10 equiv., 13.2 mL, 129.75 mmol) was added dropwise via the addition funnel over the course of 90 min. During this time, the solid starting material dissolved and an orange solution formed. Vigorous bubbling was observed throughout the addition. After 30 min, the mixture was allowed to cool to 23 °C. The solution was acidified to pH 2 with aqueous sulfuric acid solution (6 N). The resulting suspension was extracted with dichloromethane (3 × 1 L) and the combined organic layers were dried over anhydrous sodium sulfate. The dried solution was filtered and the filtrate was concentrated to provide 2-fluoro-5-methoxybenzoic acid (2.01 g, 11.81 mmol, 91.05% yield) as a white solid. ^1^H NMR (300 MHz, CDCl_3_) δ 7.44–7.54 (m, 1H), 7.02–7.18 (m, 2H), 3.84 (s, 3H). ^13^C NMR {^1^H} (75 MHz, CDCl_3_) δ 55.9, 115.5 (2C), 117.9, 118.2, 122.1, 122.2.

*2-Fluoro-5-methoxybenzoyl chloride* (**17**). SOCl_2_ (25 equiv., 17.48 g, 10.66 mL, 146.94 mmol) was added to a mixture of 2-fluoro-5-methoxybenzoic acid (**16**) (1.0 equiv., 1 g, 5.88 mmol) and toluene (10 mL). The mixture was stirred for 3 h at 25 °C. Then, the mixture was concentrated to afford 2-fluoro-5-methoxybenzoyl chloride (**17**) (1.108 g, 5.88 mmol, 100%) as a yellow oil. This product was used without another purification. ^1^H NMR (200 MHz, CDCl_3_) δ 7.54–7.44 (m, 1H), 7.18–7.01 (m, 2H), 3.84 (3H). ^13^C NMR {^1^H} (75 MHz, CDCl_3_) δ 169.6, 169.5, 158.9, 155.6, 155.5, 155.5, 122.3, 122.2, 118.3, 118.0, 117.8, 117.6, 115.7, 77.6, 77.2, 76.7, 56.1.

*(1,4-Dimethoxy)-3-methylnaphthalen-2-yl)(2-fluoro-5-methoxyphenyl)methanone* (**18**). 2-bromo-1,4-bis(methoxymethoxy)-3-methylnaphthalene (**8**) (1.0 equiv., 672 mg, 1.97 mmol) was dissolved in dry THF (12 mL) under an argon atmosphere, and the solution was cooled to −78 °C. *n*-BuLi (1.1 equiv., 1.35 mL, 2.17 mmol) was added dropwise, and the reaction mixture was stirred for 45 min at −78 °C. 2-fluoro-5-methoxybenzoyl chloride (**16**) (1.14 equiv., 423.41 mg, 2.25 mmol) was then added dropwise at −78 °C, and the reaction mixture was stirred for 1.5 h at this temperature. The reaction was quenched by pouring the mixture into saturated aqueous NH_4_Cl. The phases were separated, and the aqueous phase was extracted with diethyl ether. The combined organic layers were washed with saturated aqueous NaHCO_3_, then with saturated aqueous NaCl, dried over anhydrous MgSO_4_, filtered, and concentrated under reduced pressure. The crude product was purified by silica gel column chromatography (cyclohexane/ethyl acetate, 5:1) to afford the desired compound **18** as a yellow solid (693 mg, 81% yield). ^1^H NMR (400 MHz, CDCl_3_) δ 8.14 (d, *J* = 8.2 Hz, 1H), 8.11 (d, *J* = 8.2 Hz, 1H), 7.56–7.60 (m, 1H), 7.40–7.50 (m, 1H), 7.36 (dd, *J* = 5.8, 3.2 Hz, 1H), 7.07–7.11 (m, 1H), 6.99–7.04 (m, 1H), 5.15 (s, 2H), 5.02 (s, 2H), 3.81 (s, 3H), 3.68 (s, 3H), 3.40 (s, 3H), 2.32 (s, 3H). ^13^C NMR {^1^H} (100 MHz, CDCl_3_) δ 193.4 (d, *J* = 1.6 Hz), 158.0, 156.6 (d, *J* = 2.3 Hz), 155.4, 148.3, 146.7 (d, *J* = 1.6 Hz), 133.1, 128.7 (d, *J* = 224.8 Hz), 127.3, 126.1, 123.6 (d, *J* = 1.8 Hz), 123.0, 122.5, 121.5 (d, *J* = 8.8 Hz), 117.9 (d, *J* = 24.7 Hz), 114.2, 101.0, 100.2, 57.9, 57.6, 55.9, 13.8. ^19^F NMR (376 MHz, CDCl_3_) δ −121.3. EI-MS (70 eV) *m*/*z* (%) 153.0 (100), 337.1 (83), 198.1 (81), 414.1 ([M]^+^, 47).

*2-[(2-Fluoro-5-methoxyphenyl)carbonyl]-4-(methoxymethoxy)naphthalen-1-ol* (**19**). ((1,4-bis(methoxymethoxy)-3-methylnaphthalen-2-yl)(2-fluoro-5-methoxyphenyl)methanone (**18**) 1.0 equiv., 447 mg, 1.079 mmol) was dissolved in benzene (11 mL) under an argon atmosphere at room temperature, and MgBr_2_·OEt_2_ (1.1 equiv., 306 mg, 1.19 mmol) was added. After stirring for 24 h, residual starting material was still observed, and an additional portion of MgBr_2_·OEt_2_ (1.5 equiv., 417.8, 1.62 mmol) was added. As the conversion started to increase, a further portion of MgBr_2_·OEt_2_ (1.5 equiv., 1.62 mmol 417.8) was added, and the reaction mixture was stirred overnight. The reaction was quenched by pouring the mixture into saturated aqueous NH_4_Cl. The layers were separated, and the aqueous phase was extracted with diethyl ether. The combined organic layers were washed with brine, dried over anhydrous Na_2_SO_4_, filtered, and concentrated under reduced pressure to afford compound **29** (383 mg, 1.035 mmol, 96% yield) as a yellow solid. ^1^H NMR (400 MHz, CDCl_3_) δ 12.65 (s, 1H), 8.46 (d, *J* = 8.3 Hz, 1H), 8.03 (d, *J* = 8.3 Hz, 1H), 7.68 (ddd, *J* = 8.3, 6.9, 1.3 Hz, 1H), 7.53 (ddd, *J* = 8.3, 6.9, 1.3 Hz, 1H), 7.01–7.08 (m, 2H), 6.93–6.98 (m, 1H), 5.06 (s, 2H), 3.80 (s, 3H), 3.63 (s, 3H), 2.07 (s, 3H). ^13^C NMR {^1^H} (100 MHz, CDCl_3_) δ 197.3, 159.0, 156.1 (d, *J* = 2.2 Hz), 153.8 (d, *J* = 246.1 Hz), 144.6, 132.5, 130.7, 130.3 (d, *J* = 15.4 Hz), 125.9, 124.9, 124.0 (d, *J* = 1.8 Hz), 122.2, 119.4 (d, *J* = 8.1 Hz), 117.5, 117.2, 116.6, 113.5 (d, *J* = 2.6 Hz), 100.3, 58.1, 56.1, 16.6. ^19^F NMR (376 MHz, CD_2_Cl_2_) δ -125.7.

*2-Methoxy-10-(methoxymethoxy)-12H-5-oxatetraphen-12-one* (**20**). 12-[(2-2-[(2-Fluoro-5-methoxyphenyl)carbonyl]-4-(methoxymethoxy)naphthalen-1-ol (1.0 equiv., 280 mg, 0.79 mmol) was dissolved in acetone (33.33 mL), and K_2_CO_3_ (1.1 equiv., 119 mg, 0.86 mmol) was added. The reaction mixture was heated at 50 °C for 4 h. After cooling to room temperature, water was added, and the resulting mixture was partitioned between water and diethyl ether. The aqueous phase was extracted with diethyl ether, and the combined organic layers were washed with brine, dried over anhydrous MgSO_4_, filtered, and concentrated under reduced pressure. The crude product was purified by silica gel column chromatography using toluene/ethyl acetate (5:0.3) to afford the product **20** as a white–orange solid (190 mg, 71.9% yield). ^1^H NMR (400 MHz, CDCl_3_) δ 8.63 (d, *J* = 8.3 Hz, 1H), 8.19 (d, *J* = 8.0 Hz, 1H), 7.68–7.78 (m, 2H), 7.64 (ddd, *J* = 8.2, 6.9, 1.2 Hz, 1H), 7.55 (d, *J* = 9.1 Hz, 1H), 7.32 (dd, *J* = 9.1, 3.2 Hz, 1H), 5.15 (s, 2H), 3.92 (s, 3H), 3.71 (s, 3H), 2.99 (s, 3H). ^13^C NMR {^1^H} (100 MHz, CDCl_3_) δ 193.4 (d, *J* = 1.6 Hz), 158.0, 156.6 (d, *J* = 2.3 Hz), 155.4, 148.3, 146.7 (d, *J* = 1.6 Hz), 133.1, 128.7 (d, *J* = 224.8 Hz), 127.3, 126.1, 123.6 (d, *J* = 1.8 Hz), 123.0, 122.5, 121.5 (d, *J* = 8.8 Hz), 117.9 (d, *J* = 24.7 Hz), 114.2, 101.0, 100.2, 57.9, 57.6, 55.9, 13.8. ^19^F NMR (376 MHz, CDCl_3_) δ −121.3. EI-MS (70 eV) *m*/*z* (%) 153.0 (100), 337.1 (83), 198.1 (81), 414.1 ([M]^+^, 47).

*3,6-Dimethoxy-2-nitrobenzaldehyde* (**21**). At 0 °C, nitric acid (7.39 equiv., 28 g, 18.6 mL, 445 mmol), Ac_2_O (19.7 mL) and 2,5-dimethoxybenzaldehyde (1.0 equiv., 10 g, 60.2 mmol) were added in this order. After 2h stirring at 25 °C, the mixture was poured onto ice water (50 mL). The resulting yellow solid was filtered, washed with cold water, then cold ethanol and recrystallized from 95% ethanol to yield **21** (9.09 g, 43.05 mmol, 72%) as yellow needles. The product recrystallized with 15 mL solvent per gram. ^1^H NMR (400 MHz, CDCl_3_) δ 10.36 (s, 1H), 7.27 (d, *J* = 9.3 Hz, 1H), 7.10 (d, *J* = 9.3 Hz, 1H), 3.94 (s, 3H), 3.87 (s, 3H). ^13^C NMR {^1^H} (75 MHz, CDCl_3_) δ 186.2, 155.4, 144.7, 120.2, 114.3, 77.6, 77.2, 76.7, 57.4, 56.9, 31.0.

*[1,4-Bis(methoxymethoxy)-3-methylnaphthalen-2-yl](3,6-dimethoxy-2-nitrophenyl)methanol* (**25**). 2-Bromo-1,4-bis(methoxymethoxy)-3-methylnaphthalene (**2b**) (1.2 equiv., 300 mg, 0.879 mmol) was dissolved in dry THF (1.5 mL) under an argon atmosphere, and the solution was cooled to −78 °C. *n*-Butyllithium (1.6 M in hexanes, 1.6 equiv., 0.733 mL, 1.17 mmol) was added dropwise, and the reaction mixture was stirred for 45 min at −78 °C. The resulting organolithium solution was then added dropwise to a solution of 3,6-dimethoxy-2-nitrobenzaldehyde (**21**) (1.0 equiv., 154 mg, 0.733 mmol) in THF at −78 °C, and the reaction mixture was stirred for 1.5 h at this temperature. The reaction was quenched by pouring the mixture into saturated aqueous NH_4_Cl (15 mL). The phases were separated, and the aqueous phase was extracted with diethyl ether (2 × 15 mL). The combined organic layers were washed with saturated aqueous NaHCO_3_, then with brine, dried over anhydrous MgSO_4_, filtered, and concentrated under reduced pressure. The crude product was purified by silica gel column chromatography (cyclohexane/ethyl acetate, 4:1) to afford compound **25** as a white solid (340 mg, 0.72 mmol, 98% yield). ^1^H NMR (400 MHz, CDCl_3_) δ 8.09 (dd, *J* = 6.0, 0.6 Hz, 1H), 7.99 (dd, *J* = 6.3, 0.6 Hz, 1H), 7.49–7.50 (m, 4H), 6.62 (d, *J* = 5.1 Hz, 1H), 5.07 (m, 4H), 4.13 (dd, *J* = 5.4, 0.9 Hz, 1H), 3.87 (s, 3H), 3.67 (s, 3H), 3.48 (s, 3H), 3.22 (s, 3H), 2.40 (s, 3H). ^13^C NMR {^1^H} (101 MHz, CDCl_3_) δ 150.1, 148.2, 147.4, 145.4, 141.0, 133.7, 129.2, 126.9, 126.6, 126.5, 126.3, 125.8, 122.7, 122.6, 112.8, 111.0, 100.3, 99.6, 66.8, 58.0, 57.6, 57.0, 56.5, 13.4.

*[1,4-Bis(methoxymethoxy)-3-methylnaphthalen-2-yl](3,6-dimethoxy-2-nitrophenyl)methanone* (**28**). [1,4-bis(methoxymethoxy)-3-methylnaphthalen-2-yl](3,6-dimethoxy-2-nitrophenyl)-methanol (**25**) (1.0 equiv., 200 mg, 0.422 mmol) was dissolved in dichloromethane (4.5 mL) under an argon atmosphere at room temperature. A solution of IBX (3.0 equiv., 354 mg, 1.27 mmol) in DMSO (5 mL) was added dropwise, and the reaction mixture was stirred for 4 h until complete conversion. Dichloromethane was added, and the reaction mixture was washed with an aqueous solution of Na_2_S_2_O_3_, and then with water. The layers were separated, and the organic phase was washed with brine, dried over anhydrous Na_2_SO_4_, filtered, and concentrated under reduced pressure. The crude product was purified by silica gel column chromatography (cyclohexane/ethyl acetate, 3:1) to afford compound **28** as a red solid (190 mg, 0.38 mmol, 90% yield). ^1^H NMR (400 MHz, CDCl_3_) δ 8.20 (d, *J* = 6.0 Hz, 1H), 8.11 (d, *J* = 5.7 Hz, 1H), 7.58 (dt, *J* = 5.1, 0.9 Hz, 1H), 7.49 (dt, *J* = 6.3, 0.6 Hz, 1H), 7.09 (m, 2H), 5.12 (s, 2H), 4.98 (s, 2H), 3.90 (s, 3H), 3.68 (s, 3H), 3.46 (s, 3H), 3.40 (s, 3H), 2.38 (s, 3H). ^13^C NMR {^1^H} (101 MHz, CDCl_3_) δ 192.0, 152.8, 148.6, 148.2, 145.3, 132.5, 130.2, 127.8, 127.8, 126.1, 125.2, 123.8, 123.3, 122.4, 117.6, 115.4, 102.2, 100.4, 58.1, 57.9, 57.5, 57.2, 41.2, 14.1.

*2-[(3,6-Dimethoxy-2-nitrophenyl)carbonyl]-4-(methoxymethoxy)-3-methylnaphthalen-1-ol* (**29**). [1,4-bis(methoxymethoxy)-3-methylnaphthalen-2-yl](3,6-dimethoxy-2-nitrophenyl) methanone **28** (1.0 equiv., 170 mg, 0.361 mmol) was dissolved in benzene (2.66 mL) under an argon atmosphere at room temperature, and MgBr_2_·OEt_2_ (1.1 equiv., 102 mg, 0.397 mmol) was added. After stirring for 24 h, residual starting material was still observed, and an additional portion of MgBr_2_·OEt_2_ (1.5 equiv., 139 mg, 0.54 mmol) was added. As the conversion started to increase, a further portion of MgBr_2_·OEt_2_ (1.5 equiv., 139 mg, 0.54 mmol) was added, and the reaction mixture was stirred overnight. The reaction was quenched by pouring the mixture into saturated aqueous NH_4_Cl. The layers were separated, and the aqueous phase was extracted with diethyl ether. The combined organic layers were washed with brine, dried over anhydrous Na_2_SO_4_, filtered, and concentrated under reduced pressure to afford compound **29** (153 mg, 0.36 mmol, 99% yield) as a yellow solid. ^1^H NMR (400 MHz, CDCl_3_) δ 13.69 (s, 1H), 8.50 (d, *J* = 6.0 Hz, 1H), 8.02 (d, *J* = 6.3 Hz, 1H), 7.70 (dt, *J* = 5.1, 0.9 Hz, 1H), 7.52 (dt, *J* = 6.3, 0.6 Hz, 1H), 7.11 (m, 2H), 5.03 (s, 2H), 3.94 (s, 3H), 3.69 (s, 3H), 3.62 (s, 3H), 2.14 (s, 3H). ^13^C NMR {^1^H} (101 MHz, CDCl_3_) δ 195.8, 161.5, 149.7, 146.1, 144.0, 138.7, 132.9, 131.1, 127.3, 125.9, 125.2, 125.1, 124.2, 122.1, 115.8, 115.6, 115.2, 100.3, 58.1, 57.4, 56.9, 14.9.

*1,4-Dimethoxy-10-(methoxymethoxy)-11-methyl-12H-5-oxatetraphen-12-one* (**30**). 2-[(3,6-Dimethoxy-2-nitrophenyl)carbonyl]-4-(methoxymethoxy)naphthalen-1-ol **29** (1.0 equiv., 140 mg, 0.328 mmol) was dissolved in DMF (10 mL) under an argon atmosphere, and K_2_CO_3_ (1.50 equiv., 68.9 mg, 0.491 mmol) was added. After completion of the reaction, the mixture was poured into saturated aqueous NH_4_Cl (40 mL), and ethyl acetate was added to separate the layers. The aqueous phase was extracted with ethyl acetate (3 × 30 mL). The combined organic layers were washed with brine, dried over anhydrous Na_2_SO_4_, filtered, and concentrated under reduced pressure. The crude product was purified by silica gel column chromatography (toluene/diethyl ether, 4:1) to afford compound **30** as a white solid (65 mg, 0.17 mmol, 52% yield) and compound **31** (47 mg, 0.12 mmol, 36%). ^1^H NMR (400 MHz, CDCl_3_) δ 8.70 (d, *J* = 6.0 Hz, 1H), 8.20 (d, *J* = 6.0 Hz, 1H), 7.74 (dt, *J* = 5.1, 0.9 Hz, 1H), 7.66 (dt, *J* = 6.3, 0.6 Hz, 1H), 6.97 (m, 2H), 5.16 (s, 2H), 4.05 (s, 3H), 4.00 (s, 3H), 3.71 (s, 3H), 2.96 (s, 3H). ^13^C NMR {^1^H} (101 MHz, CDCl_3_) δ 178.6, 156.5, 152.2, 149.7, 147.4, 131.6, 130.0, 126.5, 126.3, 124.2, 123.9, 123.7, 123.3, 122.6, 119.1, 116.7, 105.8, 100.3, 58.2, 56.0, 15.5.

*9-Methoxy-5-(methoxymethoxy)-6-methyl-8-nitro-7H-benzo[c]xanthen-7-one* (**31**). ^1^H NMR (400 MHz, CDCl_3_) δ 8.55 (d, *J* = 8.2 Hz, 1H), 8.17 (d, *J* = 8.3 Hz, 1H), 7.75 (d, *J* = 9.1 Hz, 2H), 7.64 (t, *J* = 7.4 Hz, 1H), 7.50 (d, *J* = 9.4 Hz, 1H), 5.11 (s, 2H), 3.98 (s, 3H), 3.68 (s, 3H), 2.87 (s, 3H). ^13^C NMR {1H} (101 MHz, CDCl_3_) δ 175.2, 151.7, 148.3, 148.1, 147.4, 136.6, 132.0, 130.5, 126.8, 126.1, 123.3, 123.1, 122.8, 120.4, 119.3, 116.9, 115.5, 100.4, 58.3, 57.4, 15.4.

*10-Hydroxy-1,4-dimethoxy-11-methyl-12H-5-oxatetraphen-12-one* (**7**). 1,4-Dimethoxy-10-(methoxymethoxy)-11-methyl-12H-5-oxatetraphen-12-one **30** (1.0 equiv., 100 mg, 0.263 mmol) was dissolved in isopropanol (16 mL), and aqueous HCl (1.2 equiv., 1.25 M, 0.252 mL, 0.315 mmol) was added dropwise. Dichloromethane (8 mL) was then added to ensure complete dissolution of the substrate. As no conversion was initially observed, an additional portion of aqueous HCl (1.2 equiv., 1.25 M, 0.25 mL, 0.32 mmol) was added. The reaction mixture was heated at 45 °C and stirred for 5 h. After cooling to room temperature, the solvents were removed under reduced pressure, and the residue was dried under high vacuum to afford the desired product as a white solid (70 mg, 79% yield). ^1^H NMR (400 MHz, CDCl_3_) δ 8.61 (d, *J* = 6.0 Hz, 1H), 8.19 (d, *J* = 6.0 Hz, 1H), 7.79 (dt, *J* = 5.1, 0.9 Hz, 1H), 7.63 (dt, *J* = 6.3, 0.6 Hz, 1H), 6.89 (m, 2H), 3.98 (s, 3H), 3.93 (s, 3H), 2.96 (s, 3H).

### 4.3. Primary Screening for Antiparasitic, Antibacterial, Antifungal Evaluation (Prof. Louis Maes’ Laboratory, Antwerp)

The primary in vitro evaluation of test compounds at LMPH is performed against a broad panel of pathogens to allow proper evaluation of selectivity. Compound stock solutions are prepared in 100% DMSO at 20 mM. The compounds are serially pre-diluted (two-fold or four-fold) in DMSO followed by a further (intermediate) dilution in demineralized water to assure a final in-test DMSO concentration of <1%.

#### 4.3.1. Brief Description of the Models (Prof. L. Maes, Antwerp, Belgium) [22]

Chagas’ disease: *Trypanosoma cruzi*, Tulahuen CL2, β galactosidase strain (nifurtimox-sensitive) was used. Malaria: two strains of *P. falciparum* were used: (1) the GHA strain (Pf-GHA), derived from a Ghanese patient and chloroquine sensitive, (2) the multidrug resistant *P. falciparum* K1 strain, both with the Malstat assay [22]. Sleeping sickness: the *Trypanosoma brucei brucei* Squib 427 strain (suramin-sensitive) was used. Leishmaniasis: the Leishmania infantum MHOM/MA(BE)/67 was used. Finally, the cytotoxicity was assessed by using human fibroblasts MRC-5^SV2^ and mouse macrophages (PMM). The reference drugs were included in all drug screening plates for each disease model and displayed mean IC_50_ values (μM).

#### 4.3.2. Antiplasmodial Drug Assays

Antimalarial activities of compounds **1**, **34**, **35**, **36** and CQ, presented as reported IC_50_ values (Table 2) [3,4,21] on the chloroquine sensitive 3D7 strain, were determined in Prof. K. Becker’s laboratory by the [^3^H] hypoxanthine incorporation assay, as described [45].

#### 4.3.3. In Vitro Activity on Schistosoma Worms

Adult ex vivo Schistosoma assay: Mice (Swiss–Webster) were infected with *S. mansoni* by percutaneous exposure for 1 h to cercariae (NMRI strain) obtained from infected *Biomphalaria glabrata* snails. This study was approved by the Institutional Animal Care and Use Committee of Rush University Medical Center (17-053; Department of Health and Human Services animal welfare assurance number A-3120-01). Adult worms were isolated from infected mice as described [46,47] and cultured in RPMI medium +10% fetal calf serum (FCS), 10 mM glutamine, 100 IU/mL penicillin, and 100 µg/mL streptomycin for 24 h before compound addition. Compounds were dissolved in DMSO and added at the indicated concentrations. The culture media was replaced every day and fresh compounds added. Control worms were treated with DMSO alone. Worms were cultured for 5 days.

Motility and viability was scored daily using published methods [4,48] in which a viability score of 3 = motile, no changes to morphology, transparency and intact tegument, active ventral and oral sucker, paired, attached to surface; 2 = reduced motility and/or some damage to tegument, reduced transparency and granularity, some unpairing and loss of surface adherence; 1 = severe reduction of motility and/or damage to tegument observed, with high opacity and high granularity, few or no paired worms, more loss of surface adherence; 0 = dead, the worms appear darkened and motility of the ventral and oral sucker is absent, and they displayed no movement over several min. Assays were done in triplicate with about 10 worms in each well. This study was approved by the Institutional Animal Care and Use Committee of Rush University Medical Center (17-053; Department of Health and Human Services animal welfare assurance number A-3120-01).

## 5. Conclusions

In this work, we observed the absence of toxicity of schistodione metabolites, i.e., the methoxylated benzoxanthones, when given externally. This is due to the fact that the toxicity of schistodiones requires intracellular metabolic activation and local formation of reactive metabolites, which cannot exert the same effect when added exogenously. Therefore, no intrinsic toxicity was observed. When the benzoxanthones metabolites are supplied externally, they may exhibit limited cellular uptake, poor cell membrane permeability or extremely low solubility in aqueous media, undergo rapid detoxification (e.g., via glutathione conjugation), or decompose before reaching intracellular targets. Within the One Health framework, benzylMD-derivatives may be therefore considered environmentally non-harmful due to the absence of toxicity of their metabolites when present externally from living cells.

## 6. Patents

Two patents, given under references [1,2], were filled for the synthetic routes of preparing compounds **1**, **32**, and **39** described in this manuscript.

## Figures and Tables

**Table 1:** Reaction conditions for the S_N_Ar reaction.

Entry	Conditions	Ratio 30/31
1	rt, 24 h	2:1
2	50 °C, 3 h	3:1
3	100 °C, 1 h	4:1

**Table 2:** Antiplasmodial activities expressed as IC_50_ values of (benzo)xanthones against *P. falciparum* 3D7 strain.

Compound	Structure	IC_50_ ± SE (nM)
**1** (numbered as Cpnd 4c in ref. [3] or Cpnd 15 in ref. [4])	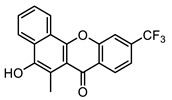	431 ± 120 [3]
**34** (numbered as Cpnd 3 in ref. [21])	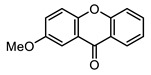	>3000 *
**35** (numbered as Cpnd 7c in ref. [3])	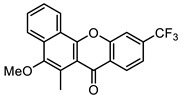	>5000 * [3]
**36** (numbered as Cpnd 1 in ref. [21])	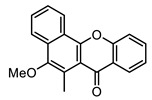	>5000 *
**plasmodione**	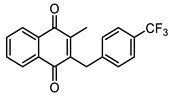	49.2 ± 6.1 [3]
**CQ**	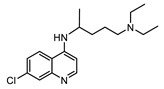	7.9 ± 1.5 [3]

*: precipitated at 5 µM in the culture medium.

**Table 3:** Antiparasitic, antibacterial, antifungal, and cytotoxic activities expressed as IC_50_ values of newly synthesized (±OMOM-protected) 3-benzoylMDs and benzoxanthones.

Compound		Structure	Solubility	*hMRC-5*	*T. cruzi*	*L. infantum*	*T. brucei*	*P. falciparum K1* ^a^	PMM Cytotoxicity	*S. aureus*	*E. coli*	*C. albicans*	*T. rubrum*	*A. fumigatus*
**3-BenzoylMDs**	**37** (numbered as Cpnd 3c-MOM in ref. [3])	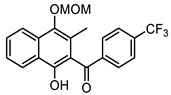	m	5.44	7.46	20.32	32.00	7.21	32.00	>64	>64	>64	2.35	>64
	**4b** (numbered as Cpnd 23 in ref. [4])	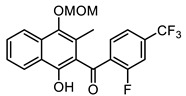	h	4.96	2.20	8.11	8.00	7.66	8.00	30.85	>64	>64	1.40	>64
	**29**	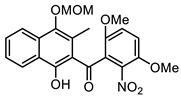	m	>64	>64	>64	>64	>64	>64	>64	>64	>64	>64	>64
**Benzoxanthones**	**38** (numbered as Cpnd 24 in ref. [4])	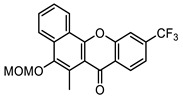	p	>64	18.15	27.27	>64	64.00	32.00	>64	>64	>64	>64	>64
	**1** (numbered as Cpnd 4c in ref. [3] or Cpnd 15 in ref. [4])	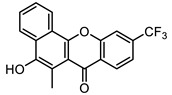	m	10.92	8.26	2.03	32.00	28.08	2.00	>64	>64	>64	7.53	>64
	**30**	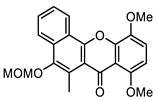	m	6.86	9.33	>64	>64	>64	>64	>64	>64	>64	>64	>64
	**31**	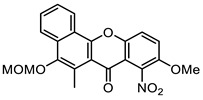	m	>64	12.85	8.11	>64	>64	8.00	8.00	>64	>64	>64	>64
**Plasmodione**		positive controls	m	>32 [22]	19.0 [22]	>32 [22]	>32 [22]	0.76 ^c^	32.00	>64	nd	nd	nd	nd
**schistodione 32 A_2′,5′_**				>64	>64	>64 ^b^	>64	>64 ^c^		>64				
**schistodione 39 A_3′,5′_**				>64	>64	>64 ^b^	>64	2.00 ^c^		>64				
**Tamoxifen**				10.88										
**Benznidazole**					2.63									
**Miltefosine**						3.1								
**Suramine**							0.02							
**Chloroquine**								0.14						
**Erythromycin**										11.30				
**Chloramphenicol**											4.90			
**Miconazole**												4.95		
**Terbinafine**													0.08	
**Miconazole**														1.61

h: No solubility issues; m: moderately soluble in 13% DMSO/Water; p: poorly soluble in 100% DMSO. ^a^: antiplasmodial assays are performed through the Malstat^TM^ reagent—PES (phenazine ethosulfaat, 0.1 mg/mL)—NBT (NitroBlue Tetrazolium Grade III, 2 mg/mL) assay principle. Change in color is measured spectrophotometrically at 655 nm.; ^b^: *L. donovani*; ^c^: *P. falciparum* GHANA strain.

## Data Availability

The original contributions presented in the study are included in the article and Supplementary Materials; further inquiries can be directed to the corresponding authors.

## Supplementary Materials

The following supporting information can be downloaded at: https://www.mdpi.com/article/10.3390/molecules31111839/s1. They include Pages S1–S22: Figure S1. Docking pose of schistodione benzoxanthone-1 **7**; and from S3 to S22: ^1^H, ^19^F and ^13^C NMR spectra of key/new compounds.
