# Supplementary material for: Synthesis of Methoxylated Benzoxanthones as Drug Metabolites of Antischistosomal Schistodiones—A Limited Environmental Risk
Source: Molecules. 2026 May 27;31(11):1839. doi: 10.3390/molecules31111839 (PMC13258496; doi:10.3390/molecules31111839)

Supplementary Materials

Article

Synthesis of Methoxylated Benzoxanthones as Drug Metabolites of Antischistosomal Schistodiones—A Limited Environmental Risk

Elena Cesar-Rodo,[1] Jeremy Boilevin,[1] Jimmy Richard,[1] Peter D. Ziniel,[2] Didier Belorgey[1], Louis Maes,[3] Francesco Angelucci,[4] David Lee Williams,[2] Elisabeth Davioud-Charvet,[1,*] Don Antoine Lanfranchi[1,*]

1 Laboratoire d’Innovation Moléculaire et Applications (LIMA), UMR7042 CNRS-Université Strasbourg-Université Haute-Alsace, Team Bio(IN)organic & Medicinal Chemistry, European School of Chemistry, Polymers and Materials (ECPM), 25, Rue Becquerel, F-67087 Strasbourg, France; elena.cesar.rodo@etu.unistra.fr (E.C.-R.); elisabeth.davioud@unistra.fr (E.D.-C.); lanfranchi@unistra.fr (D.A.L.)

2 Department of Microbial Pathogens and Immunity, Rush University Medical Center, 1735 West Harrison Street, Chicago, IL 60612, USA; david_williams@rush.edu (D.L.W.)

3 Laboratory of Microbiology, Parasitology and Hygiene (LMPH), Faculty of Pharmaceutical, Biomedical and Veterinary Sciences, University of Antwerp, Universiteitsplein 1, B-2610 Antwerp, Belgium; louis.maes@uantwerpen.be

4 Department of Life, Health and Environmental Sciences, University of L'Aquila, L'Aquila, Italy; francesco.angelucci@univaq.it

* Correspondence: elisabeth.davioud@unistra.fr (E.D.-C.); lanfranchi@unistra.fr (D.A.L.)

Content: Page S2: Figure S1. Docking pose of schistodione benzoxanthone-1 **7**; S3-S22: ^{1}H, ^{19}F and ^{13}C NMR spectra of key/new compounds.

Figure S1. Docking pose of schistodione benzoxanthone **1** (SD-BZX $A_{2',5'}$ 1, in grey sticks) obtained with the HADDOCK server (https://rascar.science.uu.nl/haddock2.4/).

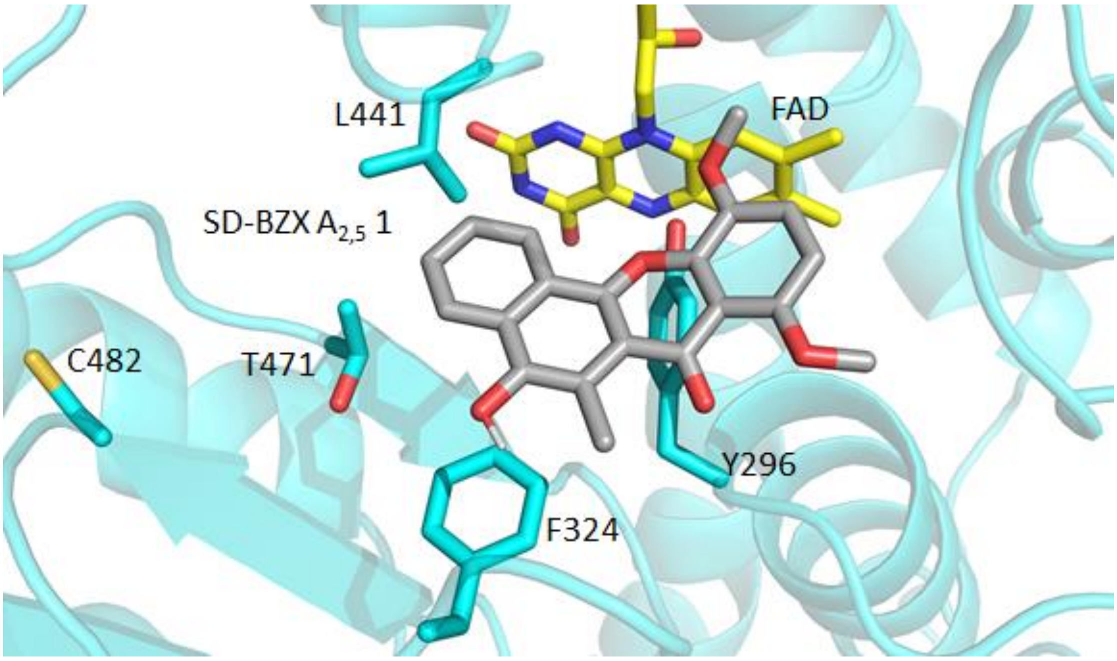

2-Bromo-3-methyl-1,4-dihydronaphthalene-1,4-dione (**8**)

^{1}H NMR ($CDCl_3$, 400 MHz)

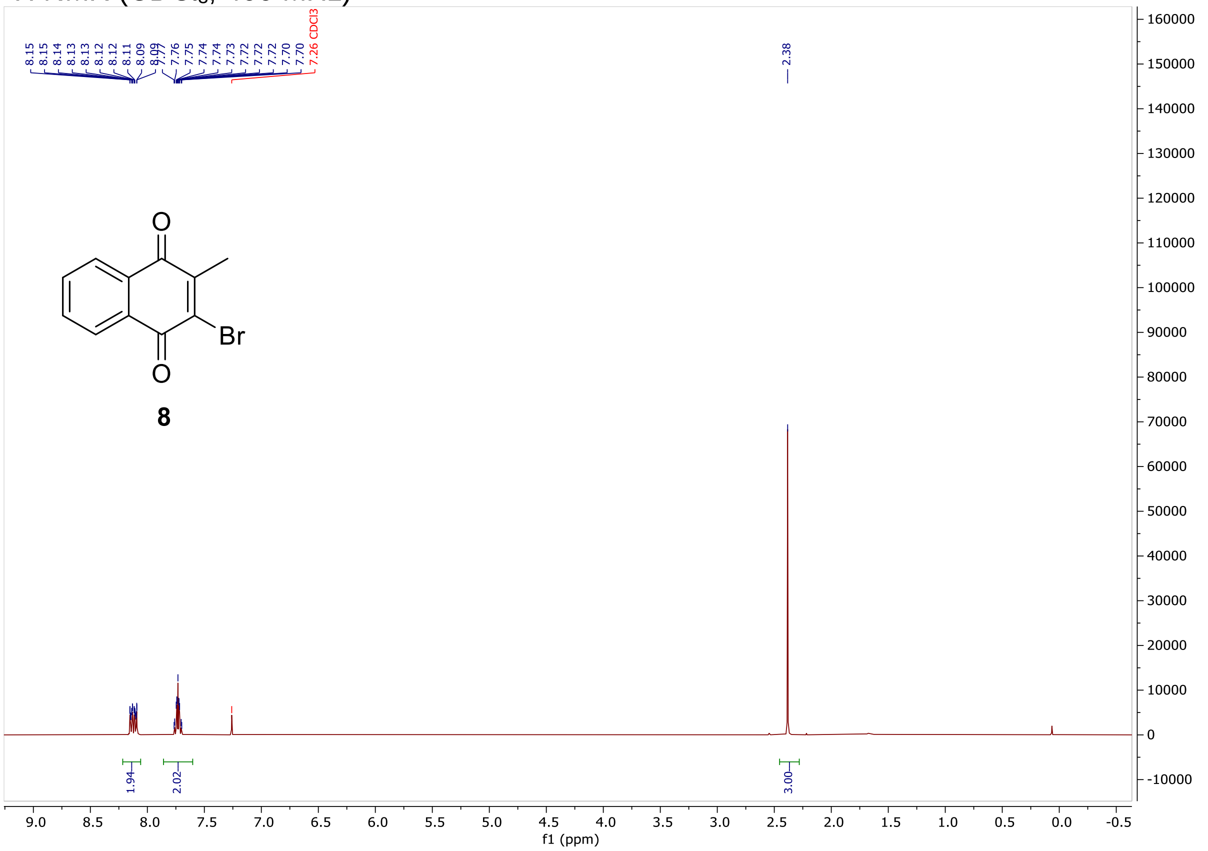

^{13}C {^{1}H} NMR ($CDCl_3$, 101 MHz)

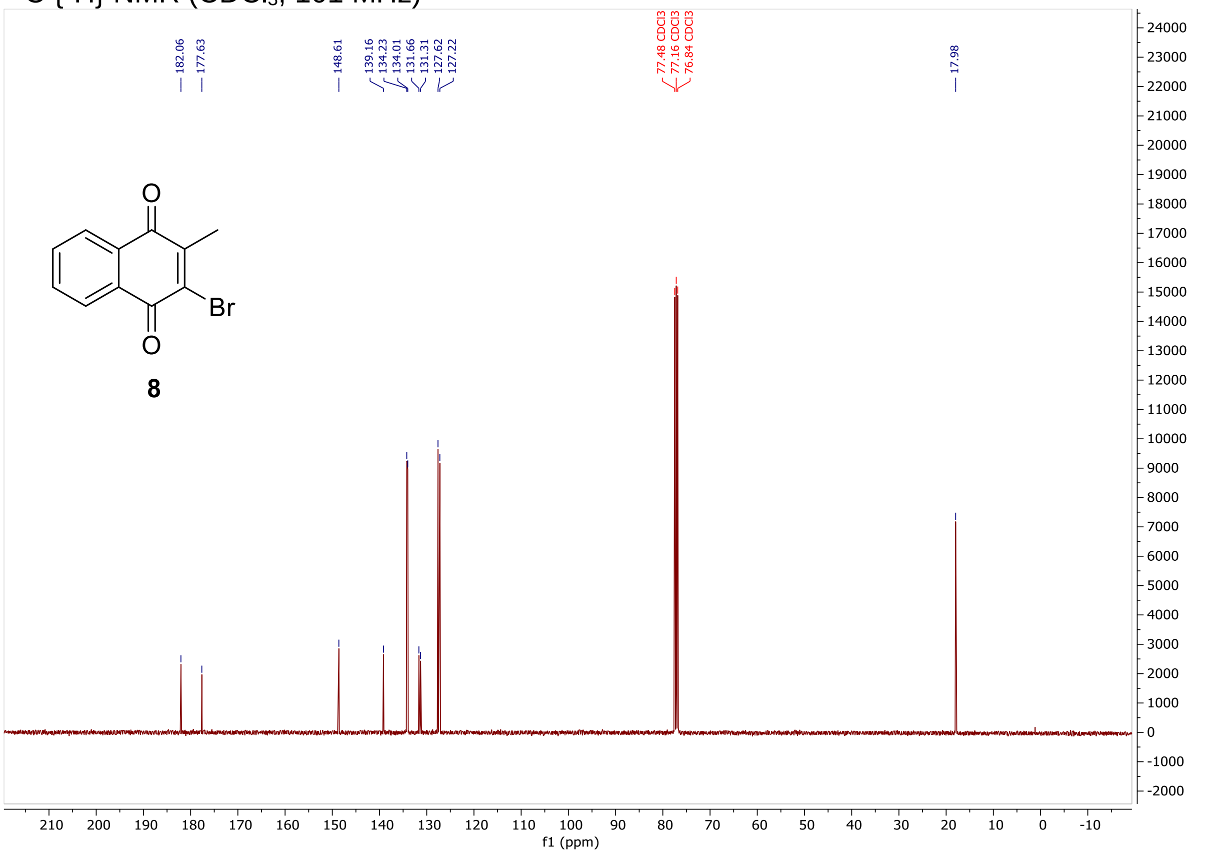

2-bromo-1,4-bis(methoxymethoxy)-3-methylnaphthalene (**2b**)

^{1}H NMR ($CDCl_3$, 400 MHz)

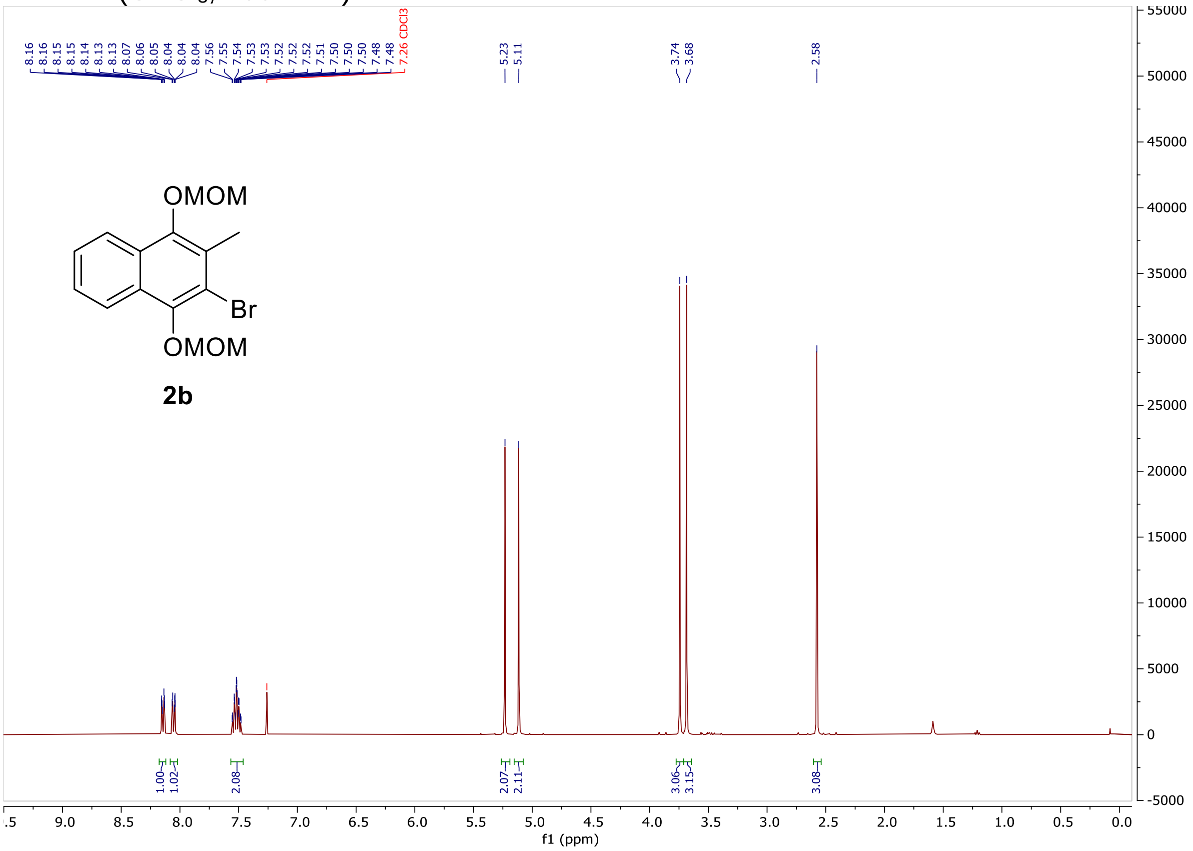

^{13}C {^{1}H} NMR ($CDCl_3$, 101 MHz)

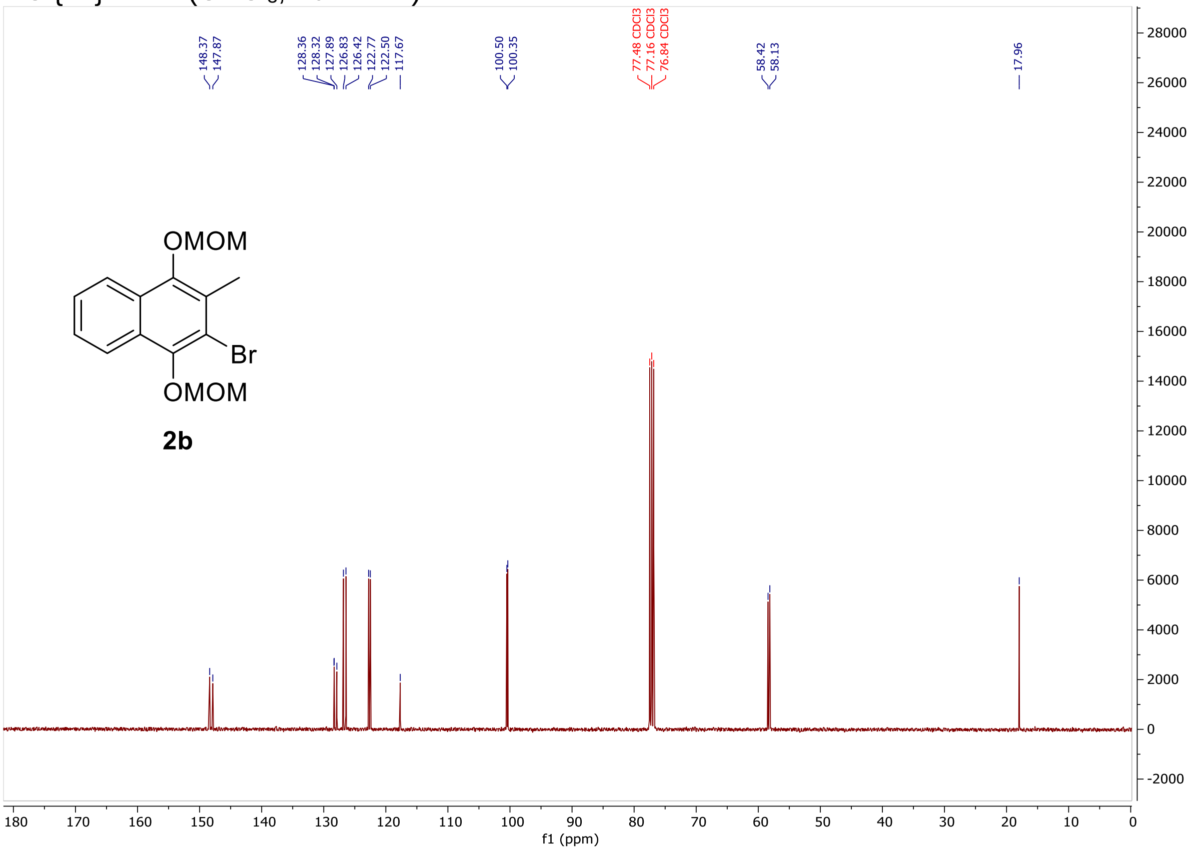

2-chloro-3,5-dimethoxybenzaldehyde (**10**)

^{1}H NMR ($CDCl_3$, 400 MHz)

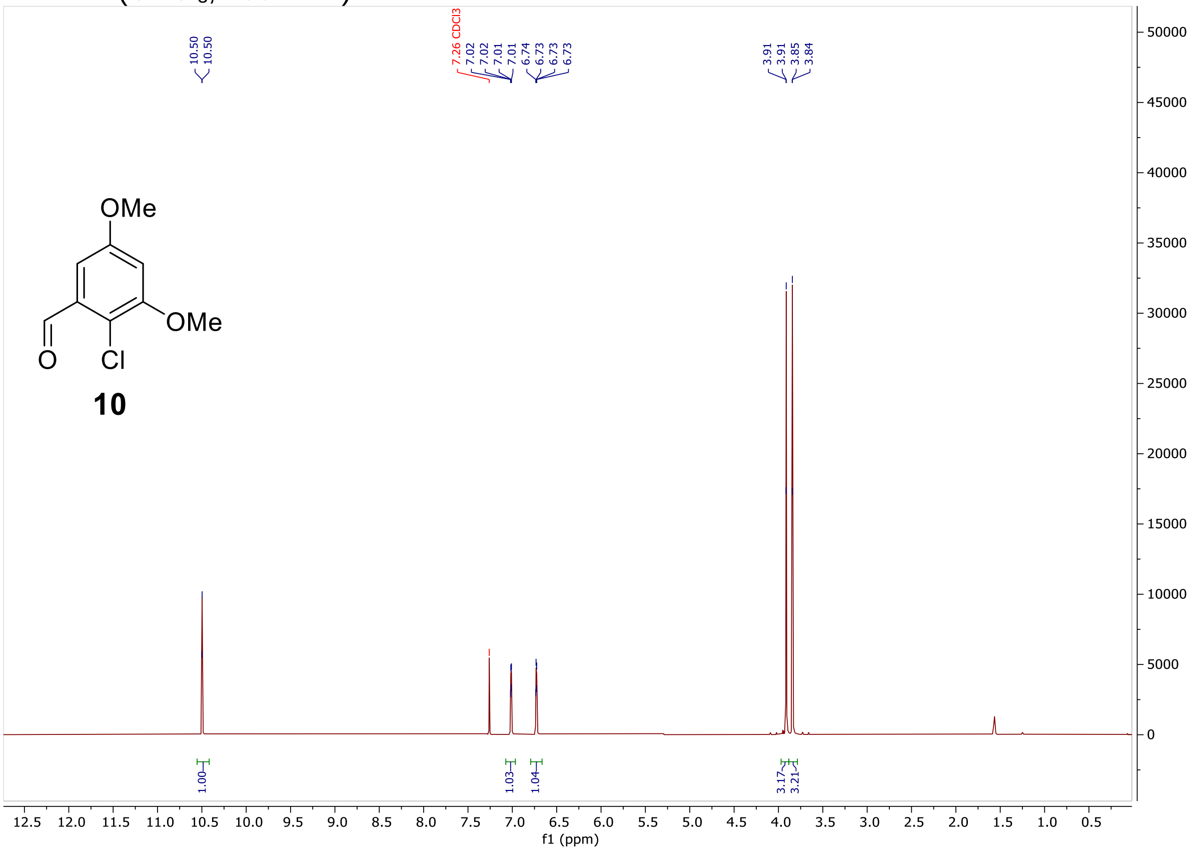

^{13}C {^{1}H} NMR ($CDCl_3$, 101 MHz)

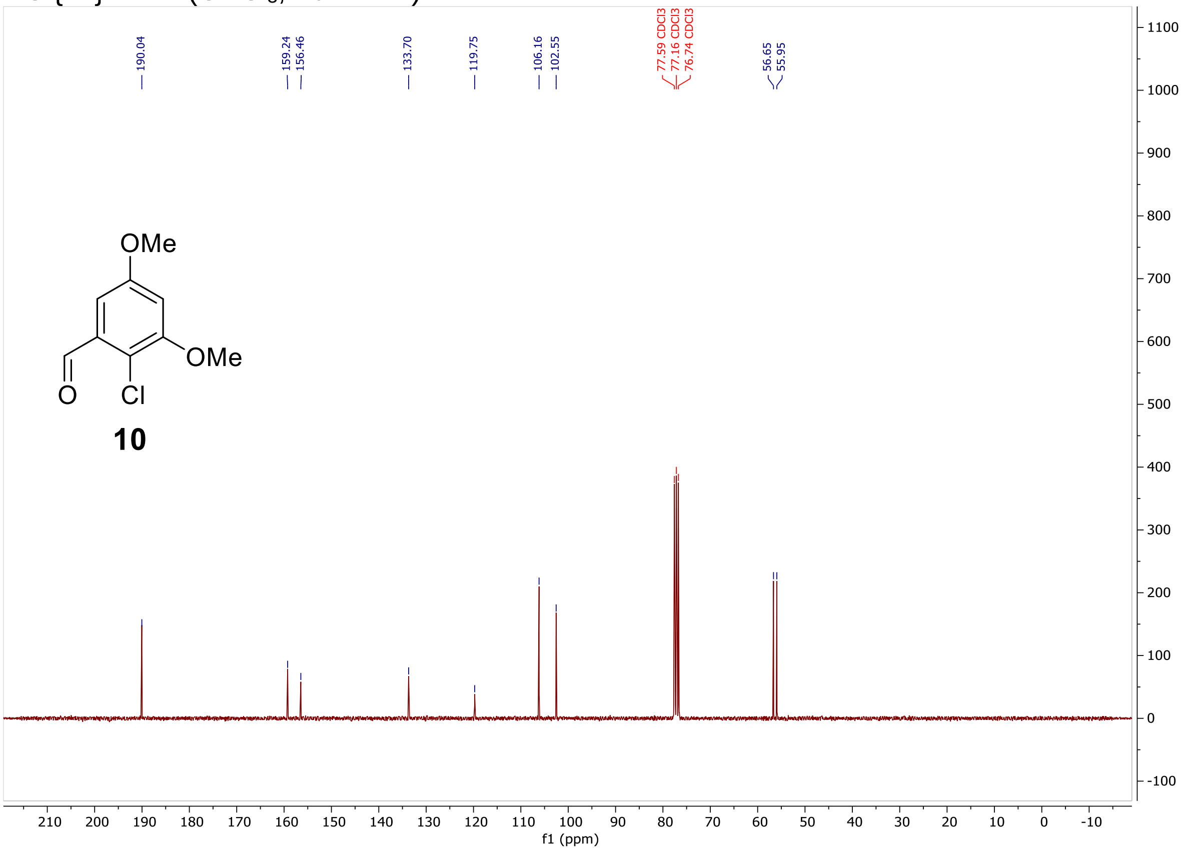

[1,4-bis(methoxymethoxy)-3-methylnaphthalen-2-yl](2-chloro-3,5-dimethoxyphenyl)methanol (**11**)

^{1}H NMR ($CDCl_3$, 400 MHz)

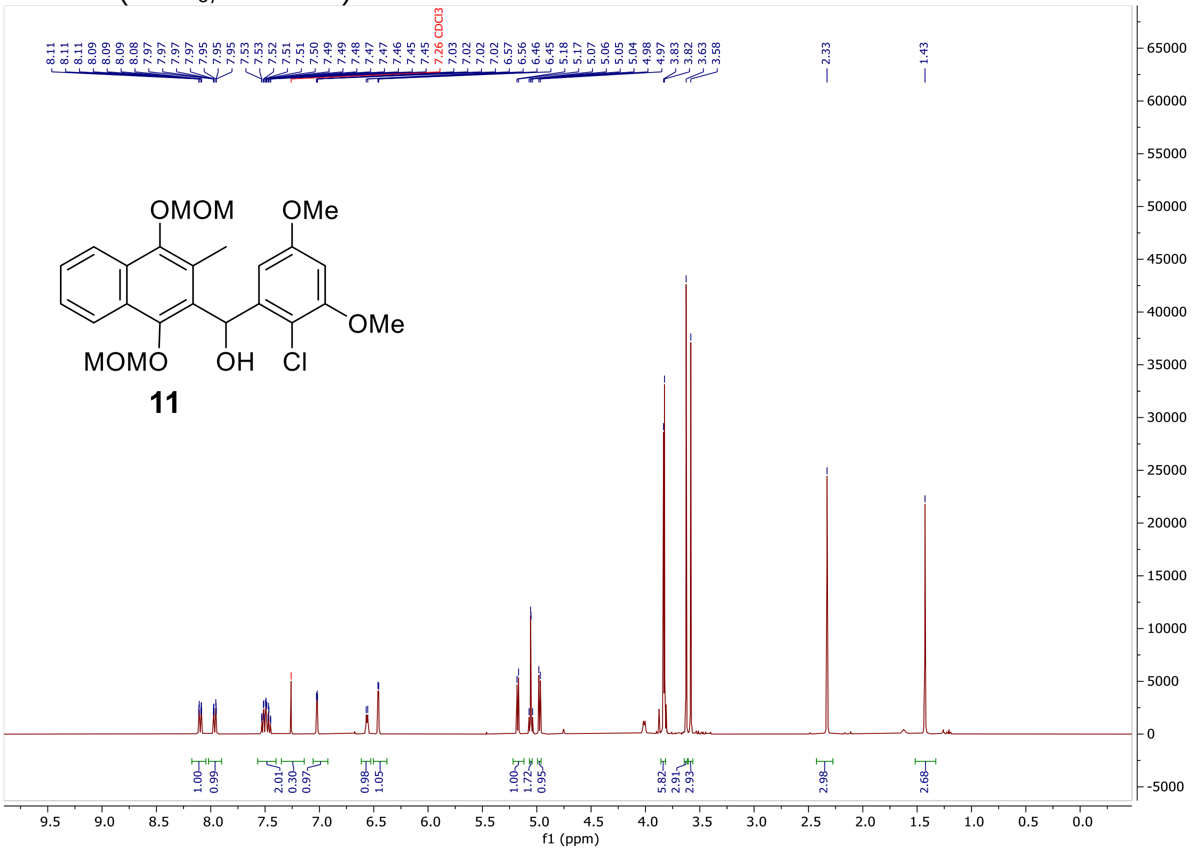

^{13}C {^{1}H} NMR ($CDCl_3$, 101 MHz)

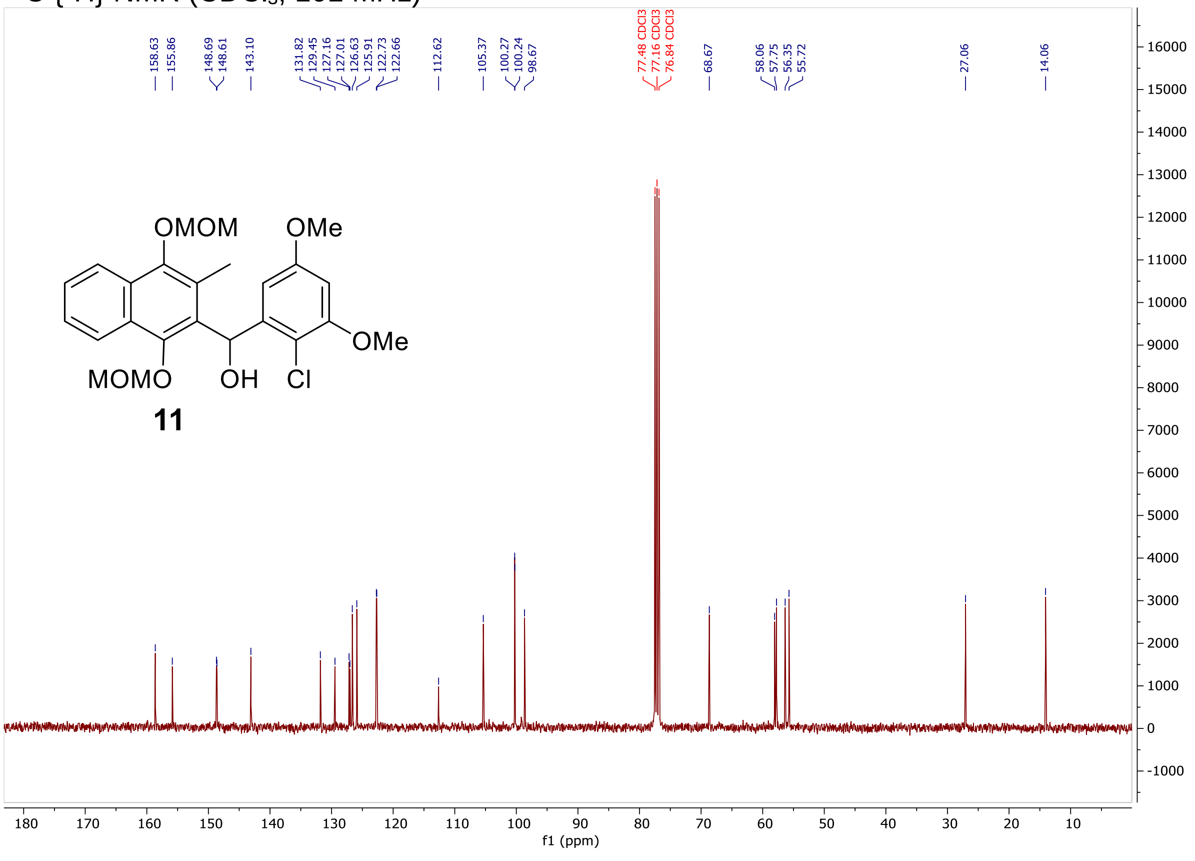

1,4-bis(methoxymethoxy)-3-methylnaphthalen-2-yl)(2-chloro-3,5-dimethoxyphenyl)methanone (**12**)

^{1}H NMR ($CDCl_3$, 400 MHz)

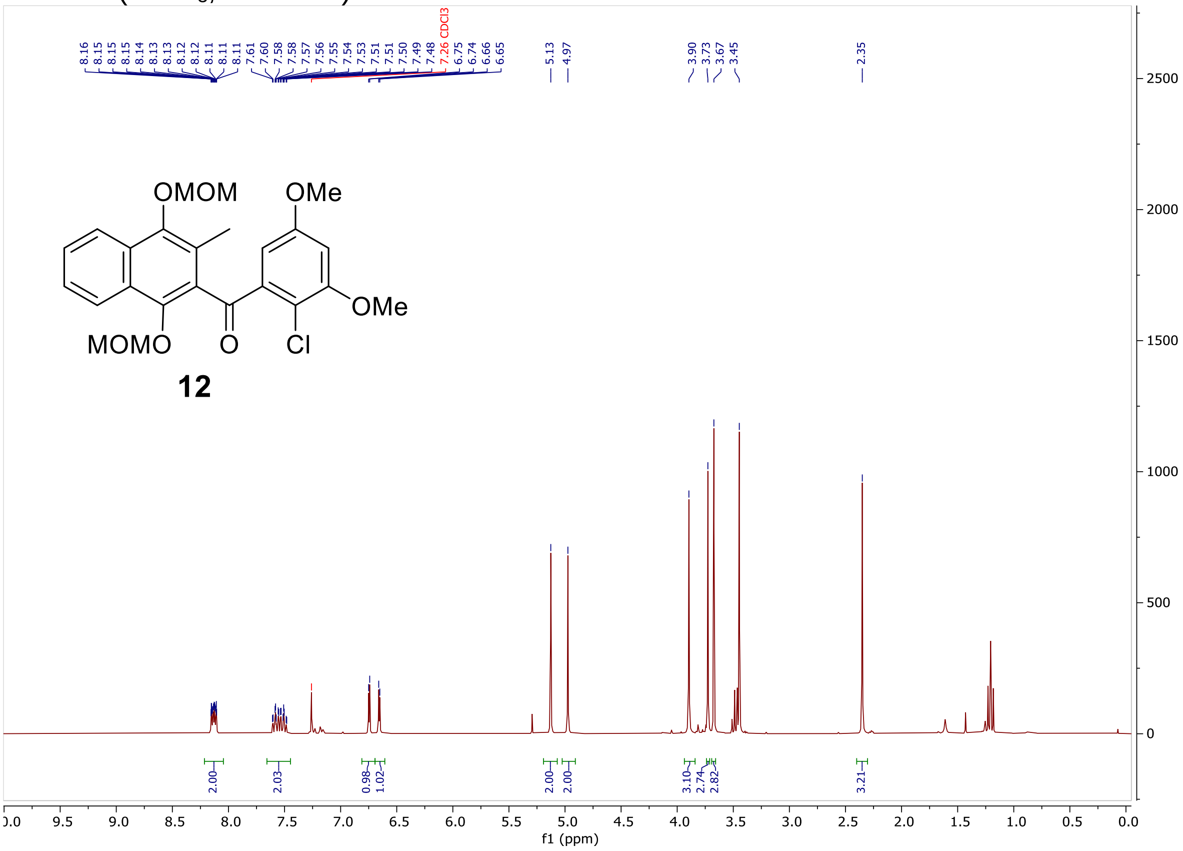

^{13}C {^{1}H} NMR ($CDCl_3$, 101 MHz)

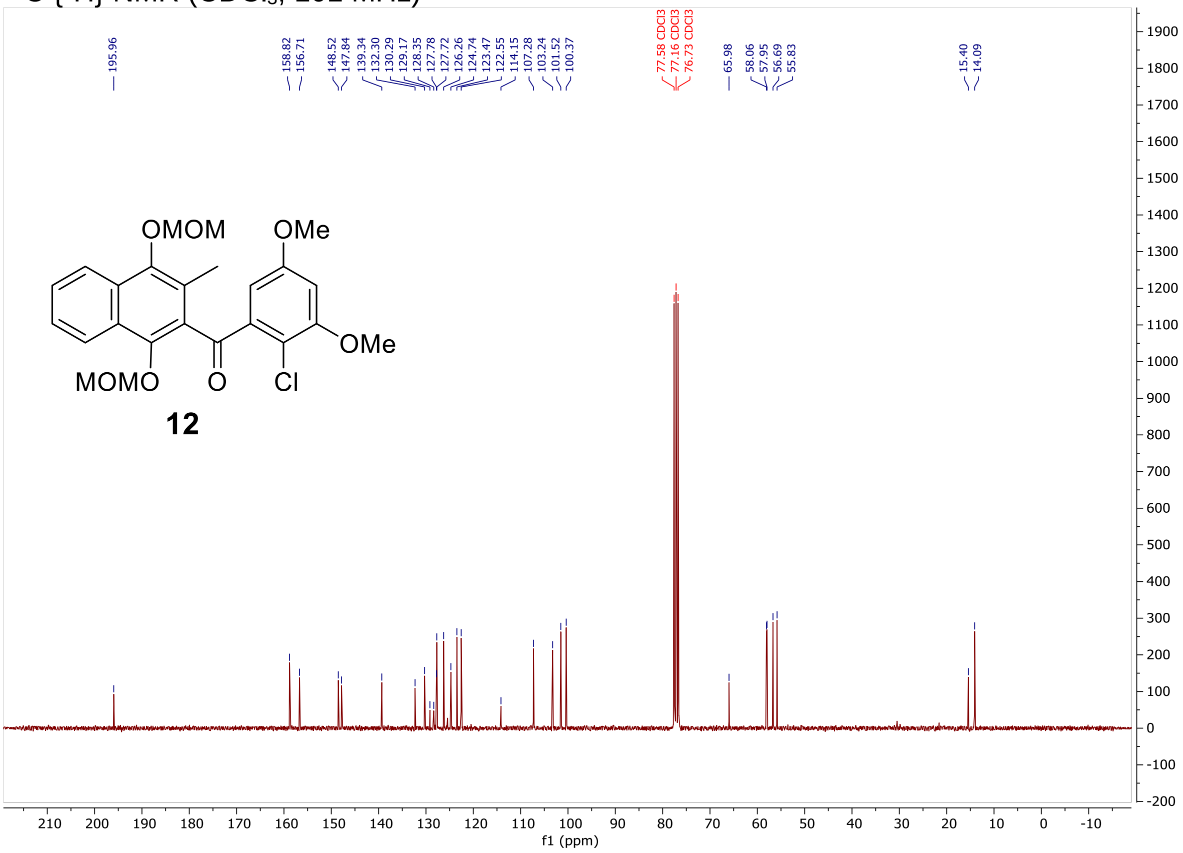

2-[(2-chloro-3,5-dimethoxyphenyl)carbonyl]-4-(methoxymethoxy)-3-methylnaphthalen-1-ol (**13**)

^{1}H NMR ($CDCl_3$, 400 MHz)

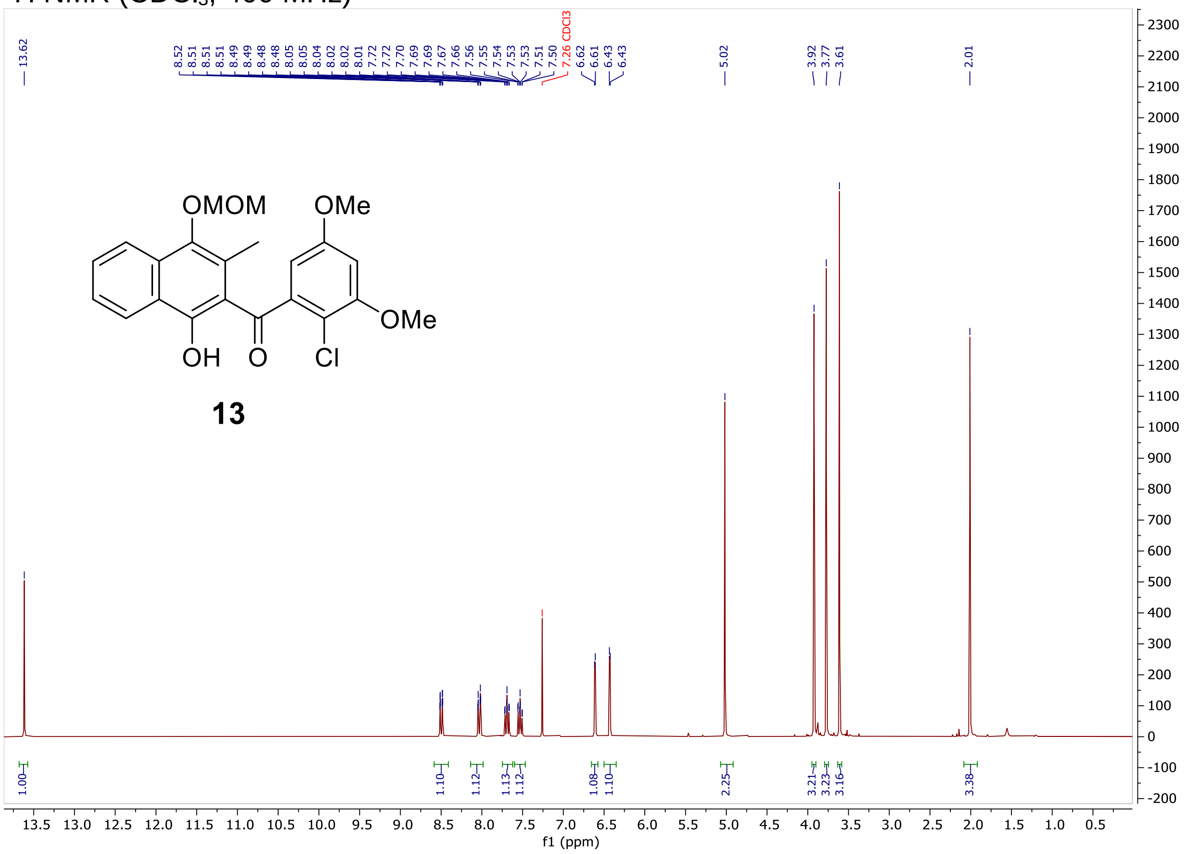

^{13}C {^{1}H} NMR ($CDCl_3$, 101 MHz)

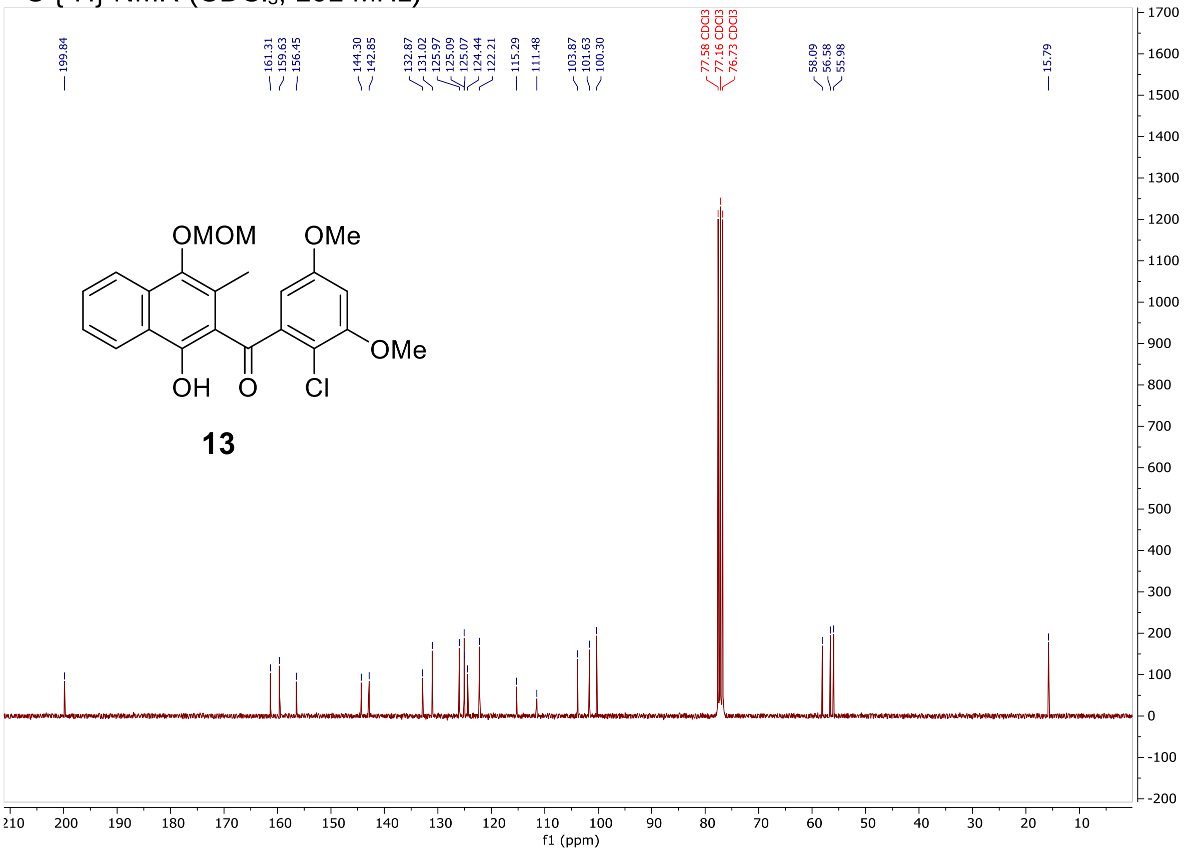

2,4-dimethoxy-10-(methoxymethoxy)-11-methyl-12H-5-oxatetraphen-12-one (**14**)

^{1}H NMR ($CDCl_3$, 400 MHz)

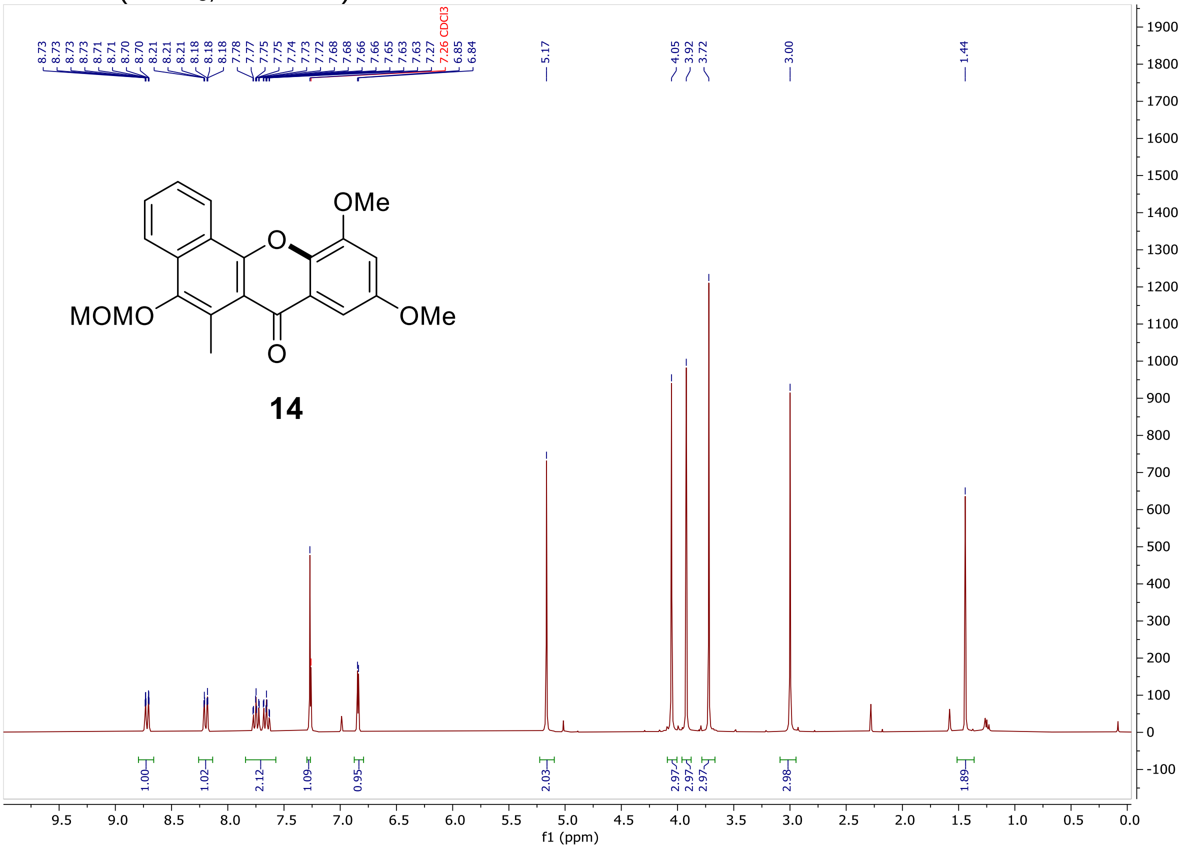

^{13}C {^{1}H} NMR ($CDCl_3$, 101 MHz)

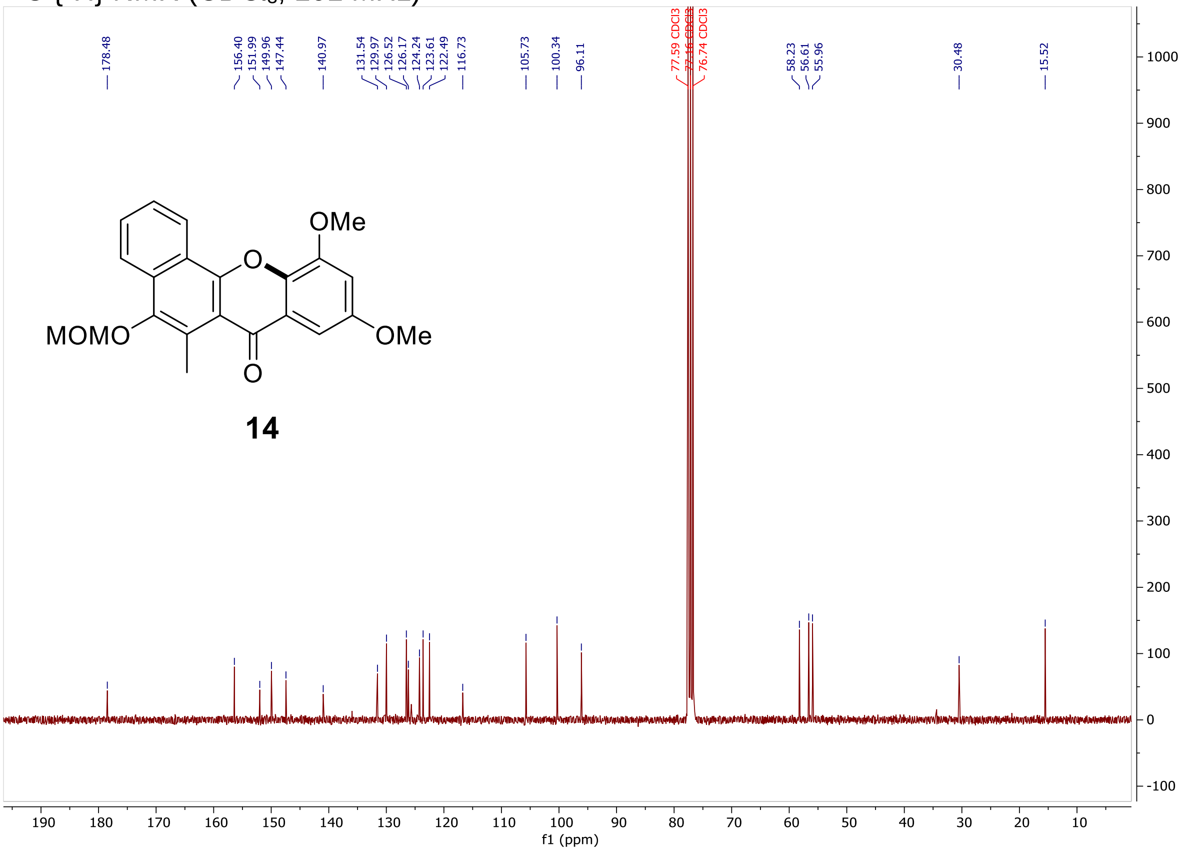

2-fluoro-5-methoxybenzoic acid (**17**)

^{1}H NMR ($CDCl_3$, 400 MHz)

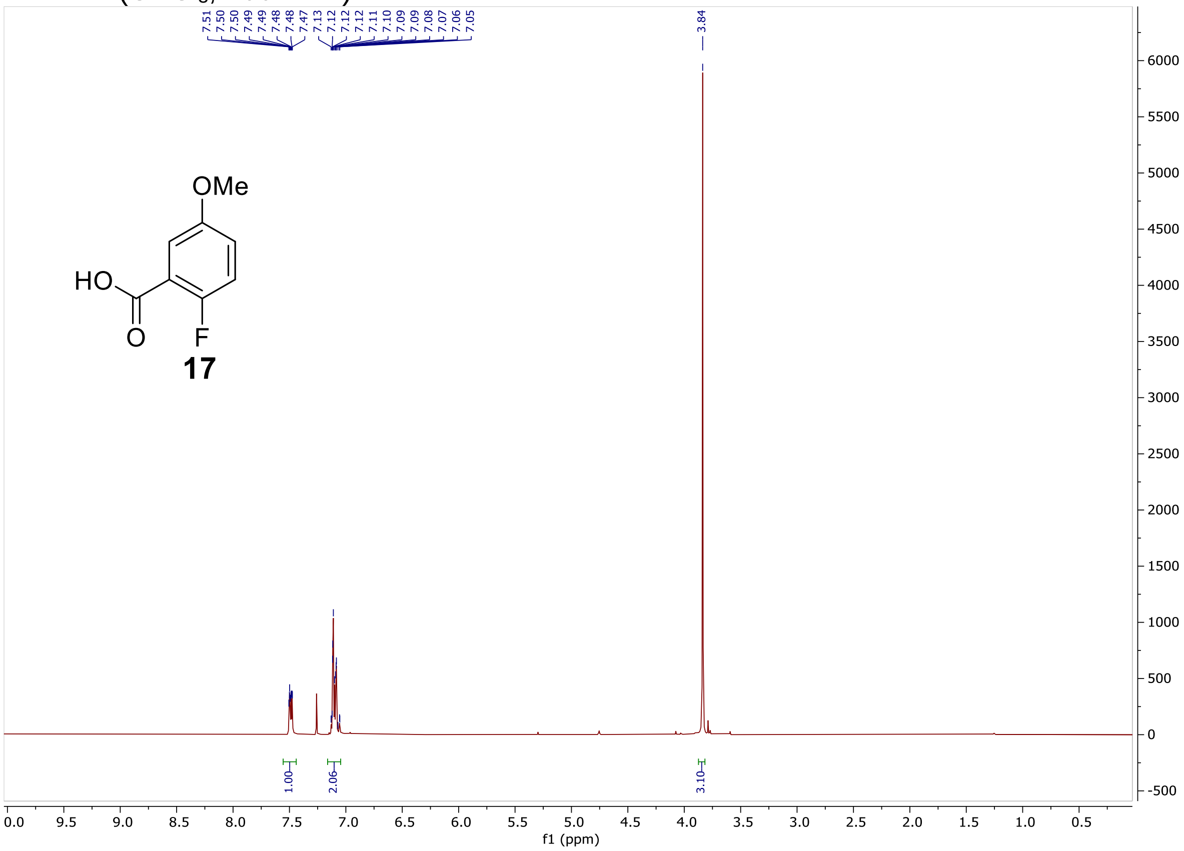

^{13}C {^{1}H} NMR ($CDCl_3$, 101 MHz)

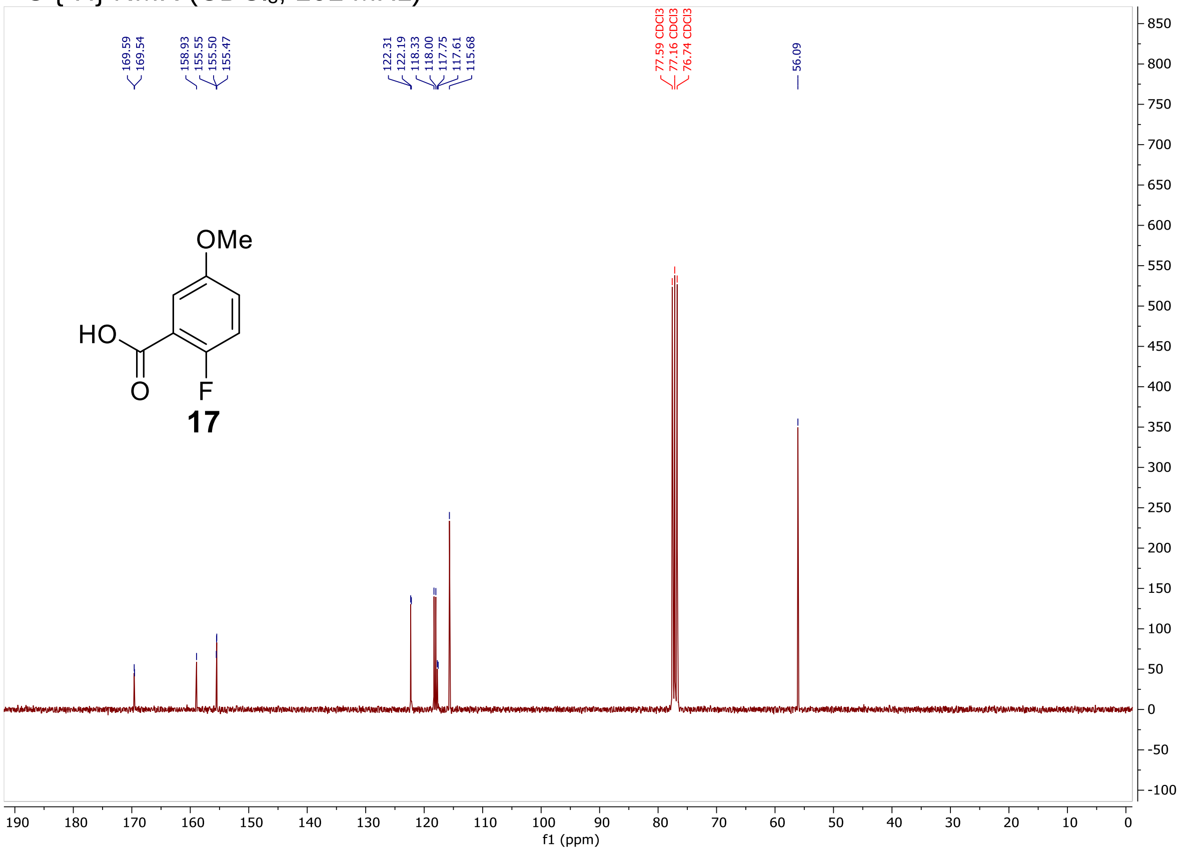

2-fluoro-5-methoxybenzoyl chloride (**16**)

^{1}H NMR ($CDCl_3$, 400 MHz)

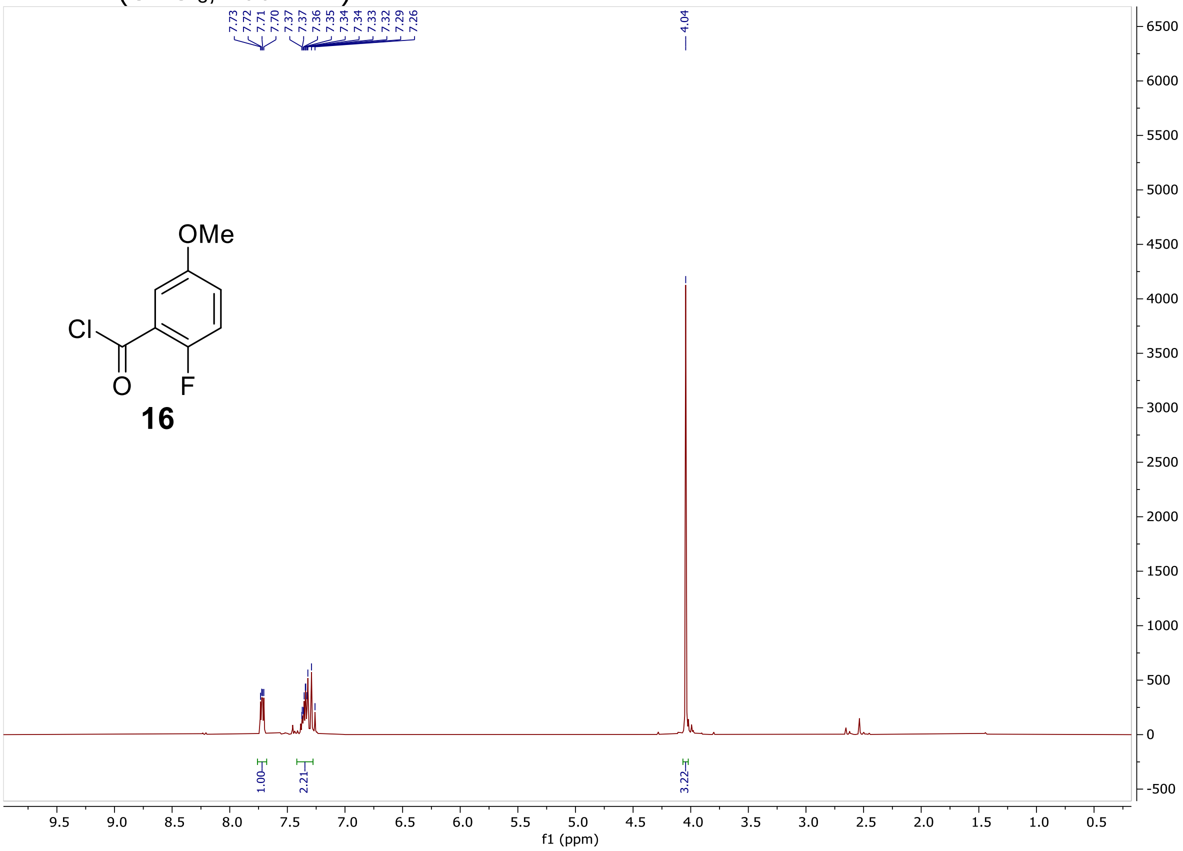

^{13}C {^{1}H} NMR ($CDCl_3$, 101 MHz)

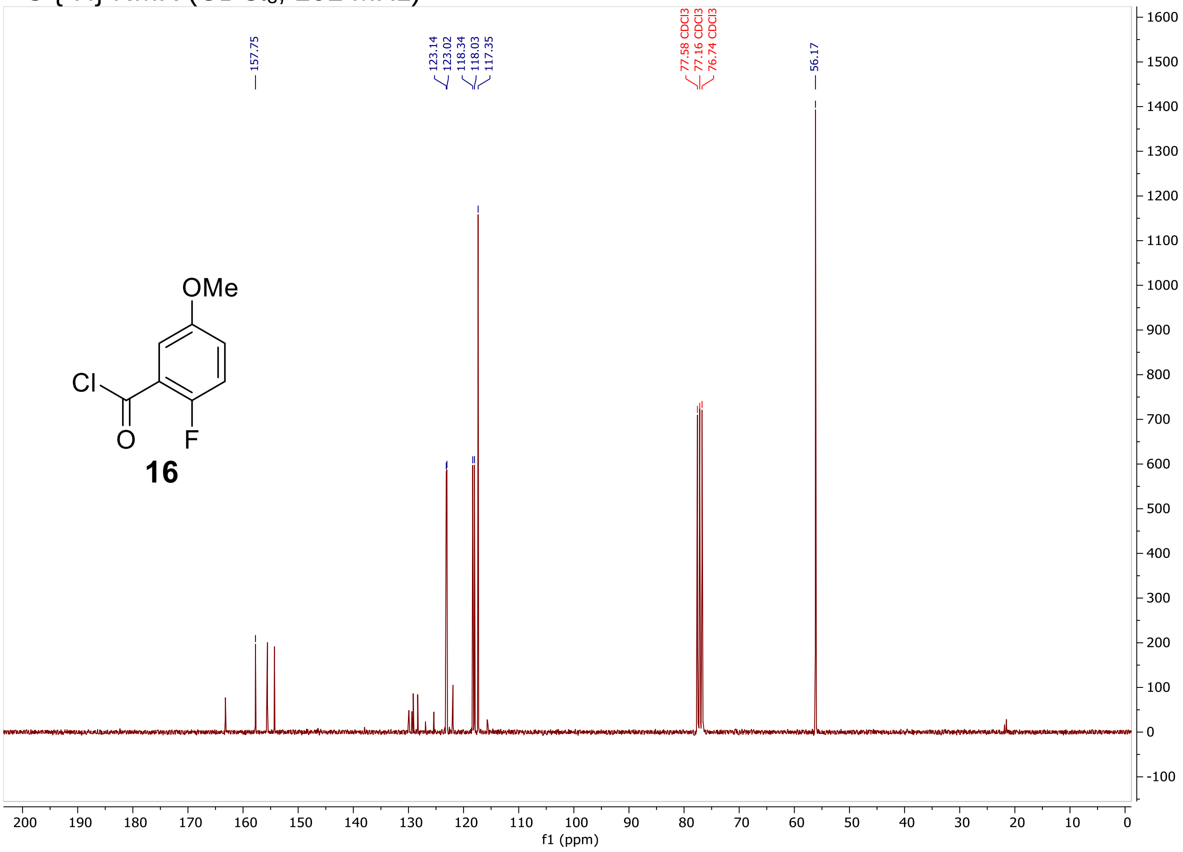

(1,4-dimethoxy)-3-methylnaphthalen-2-yl)(2-fluoro-5-methoxyphenyl)methanone (**18**)

^{1}H NMR ($CDCl_3$, 400 MHz)

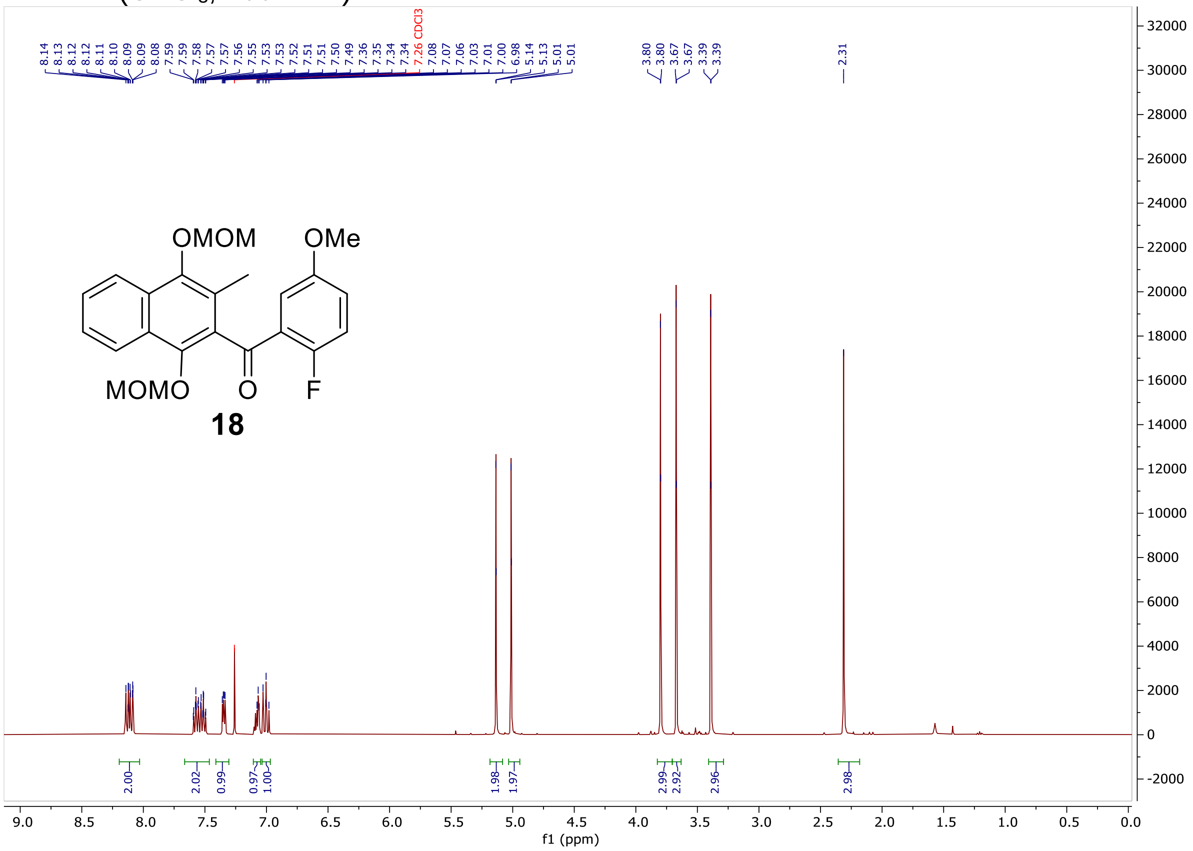

^{13}C {^{1}H} NMR ($CDCl_3$, 101 MHz)

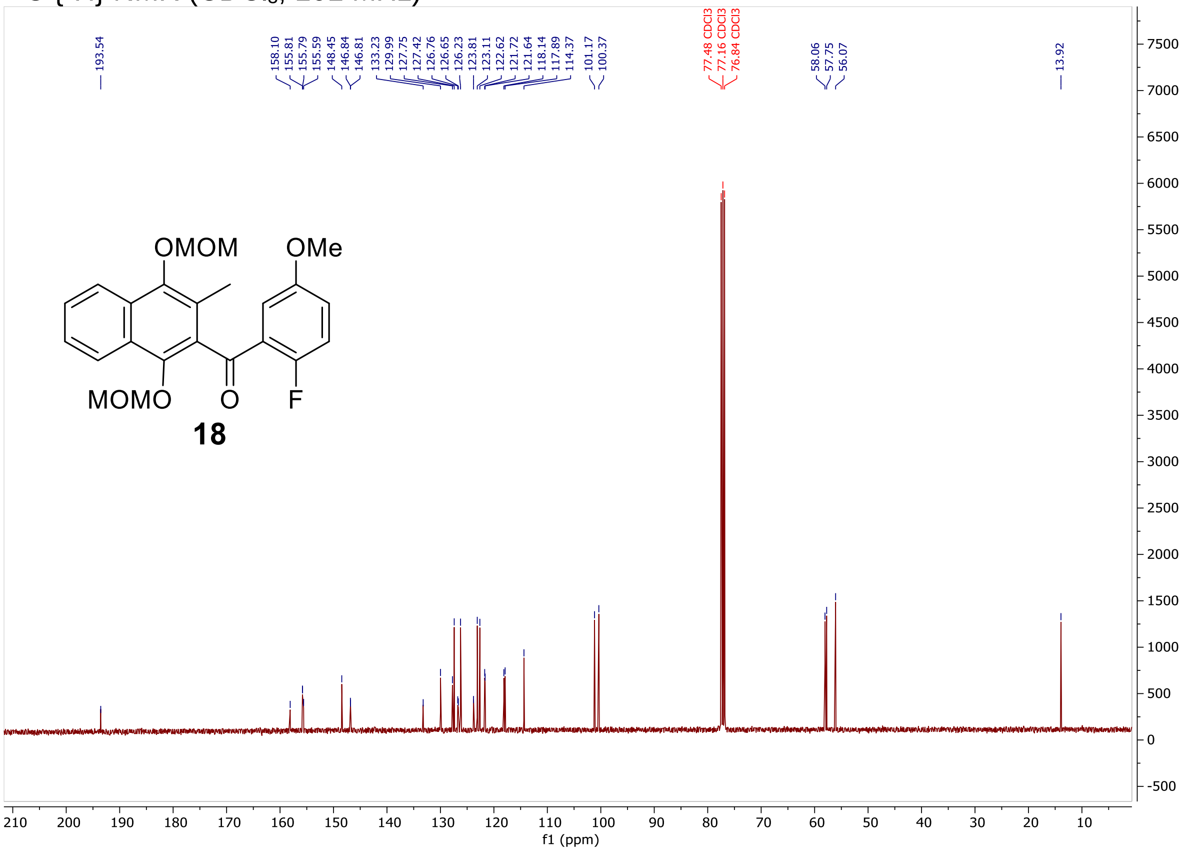

2-[(2-fluoro-5-methoxyphenyl)carbonyl]-4-(methoxymethoxy)naphthalen-1-ol (**19**)

^{1}H NMR ($CDCl_3$, 400 MHz)

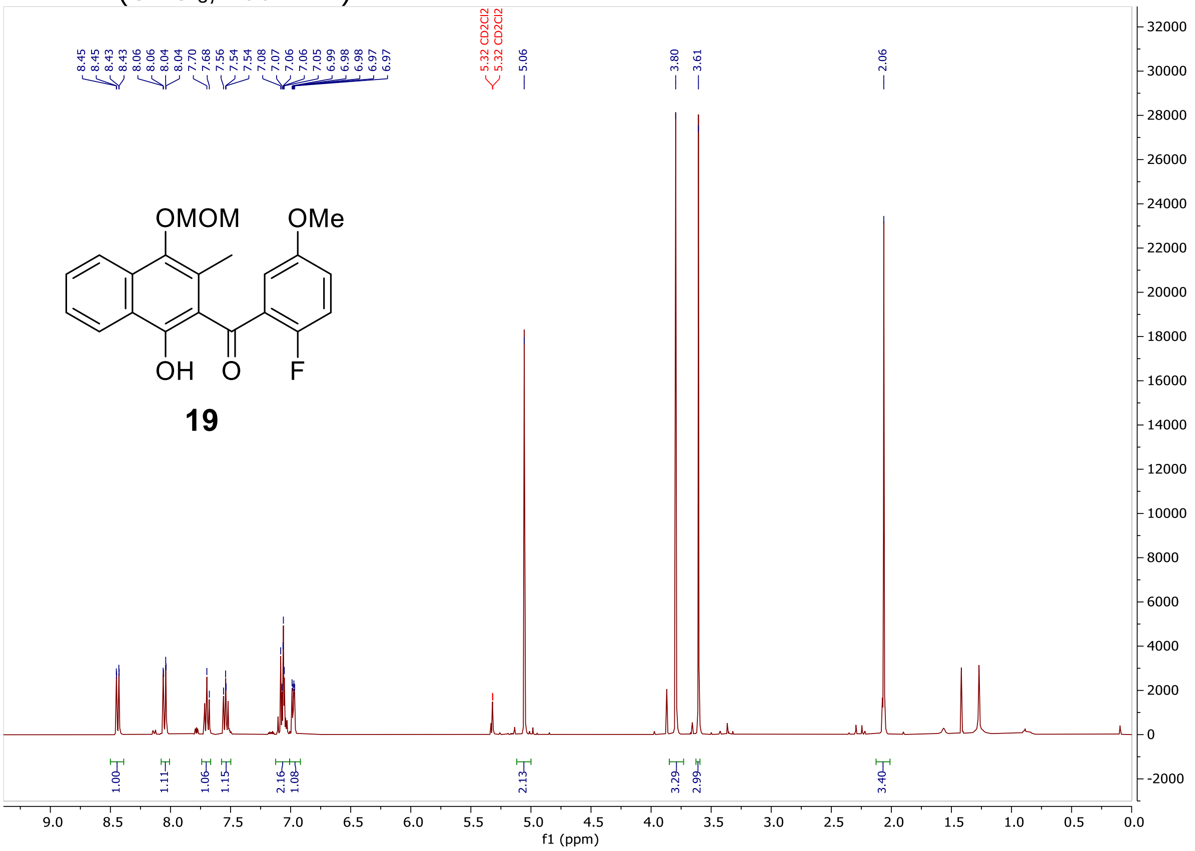

^{13}C {^{1}H} NMR ($CDCl_3$, 101 MHz)

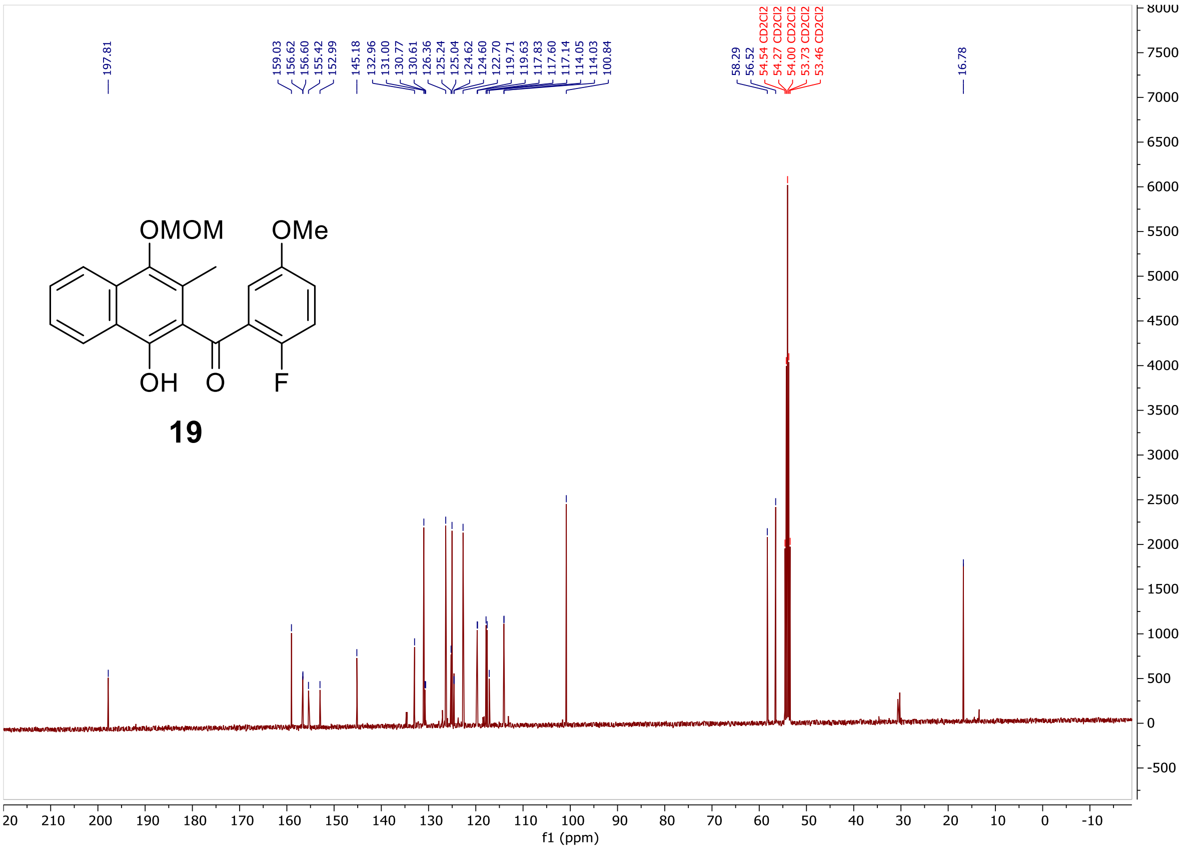

^{19}F NMR ($CDCl_3$, 377 MHz)

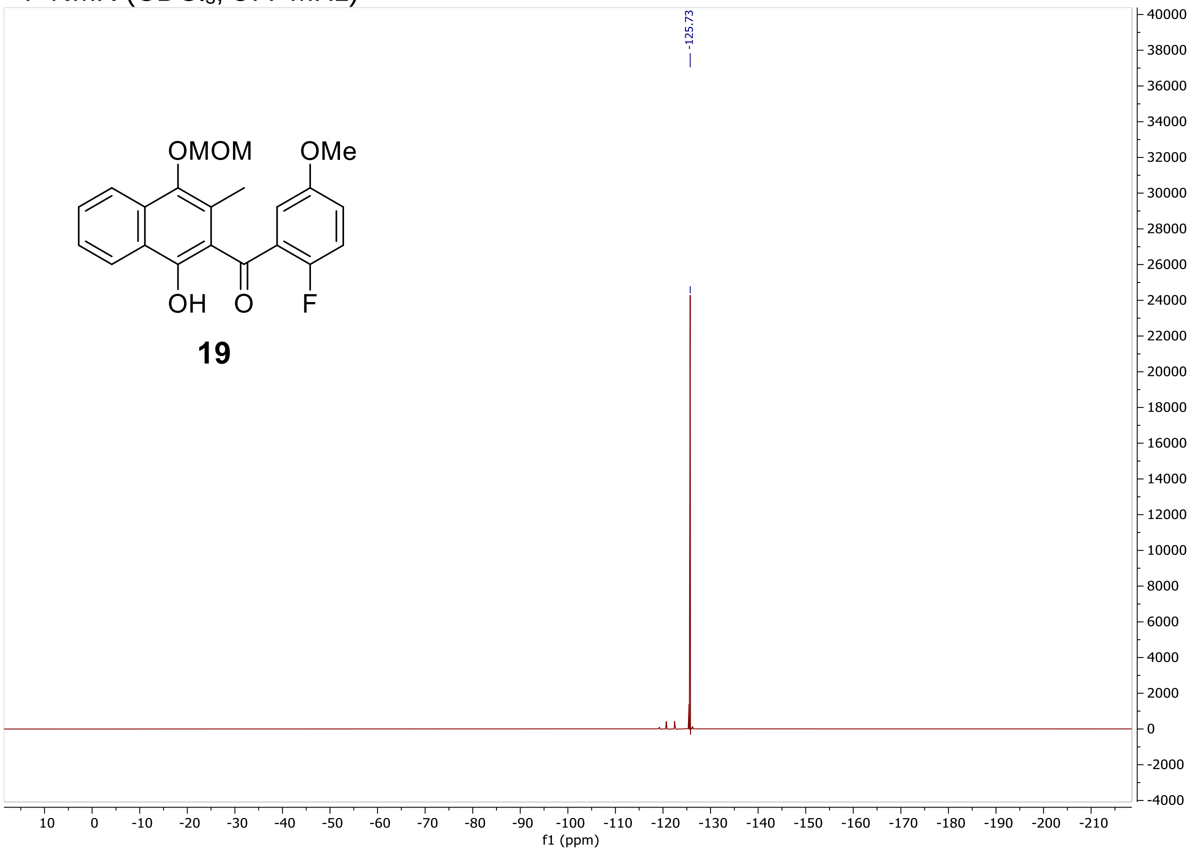

2-methoxy-10-(methoxymethoxy)-12H-5-oxatetraphen-12-one (**20**)

^{1}H NMR ($CDCl_3$, 400 MHz)

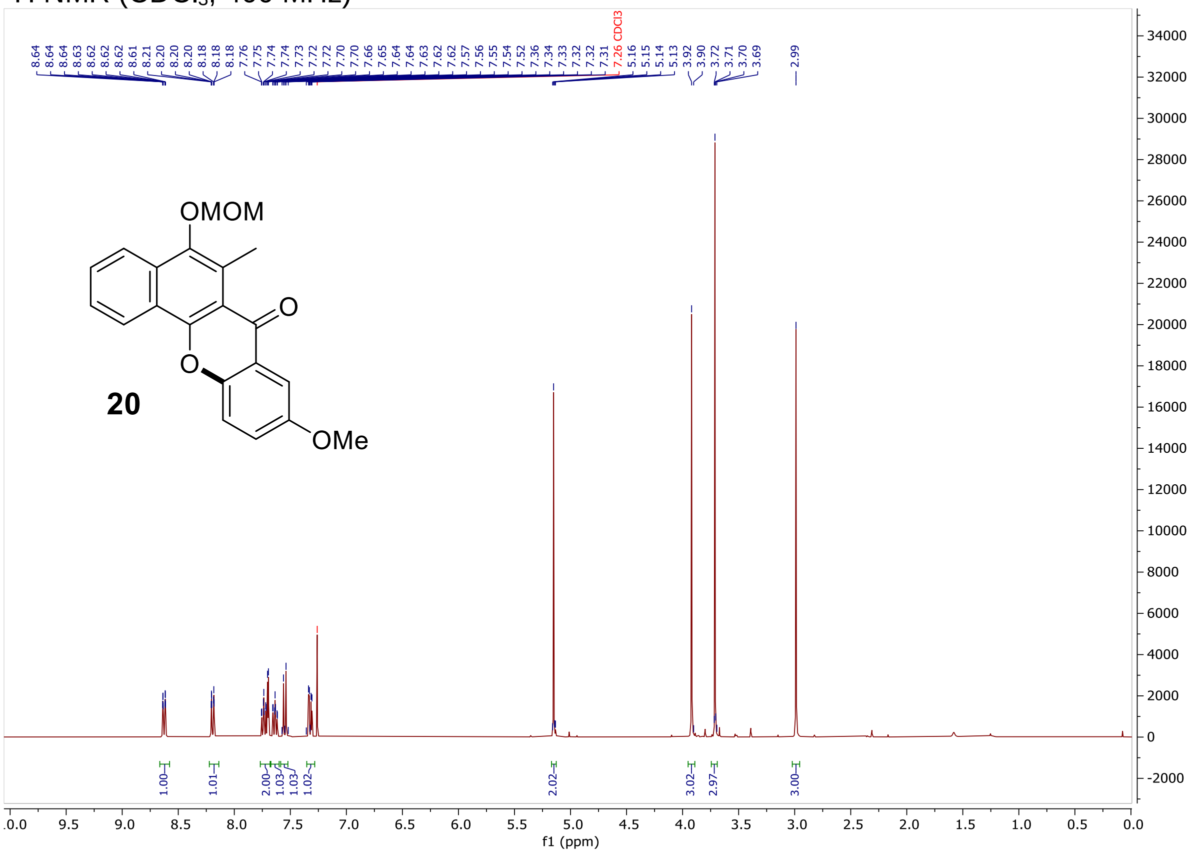

^{13}C {^{1}H} NMR ($CDCl_3$, 101 MHz)

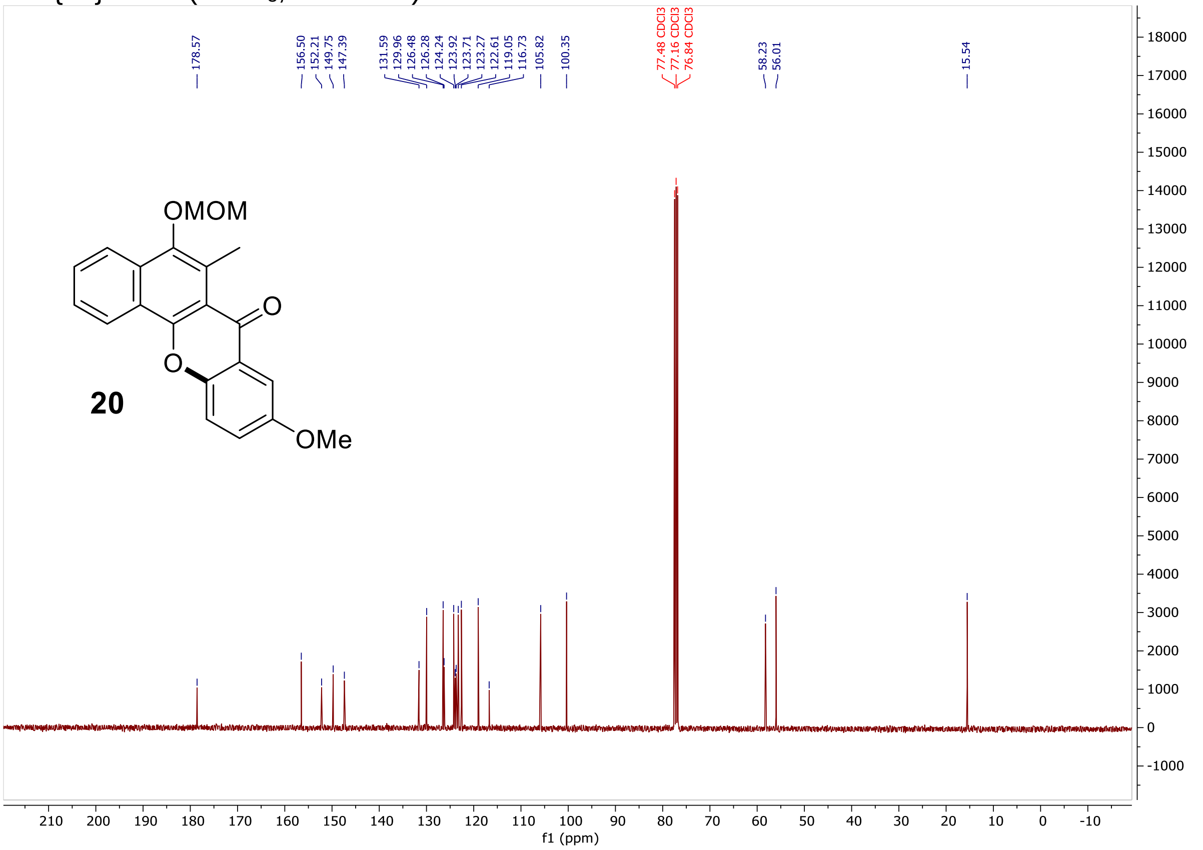

3,6-dimethoxy-2-nitrobenzaldehyde (**21**)

^{1}H NMR ($CDCl_3$, 400 MHz)

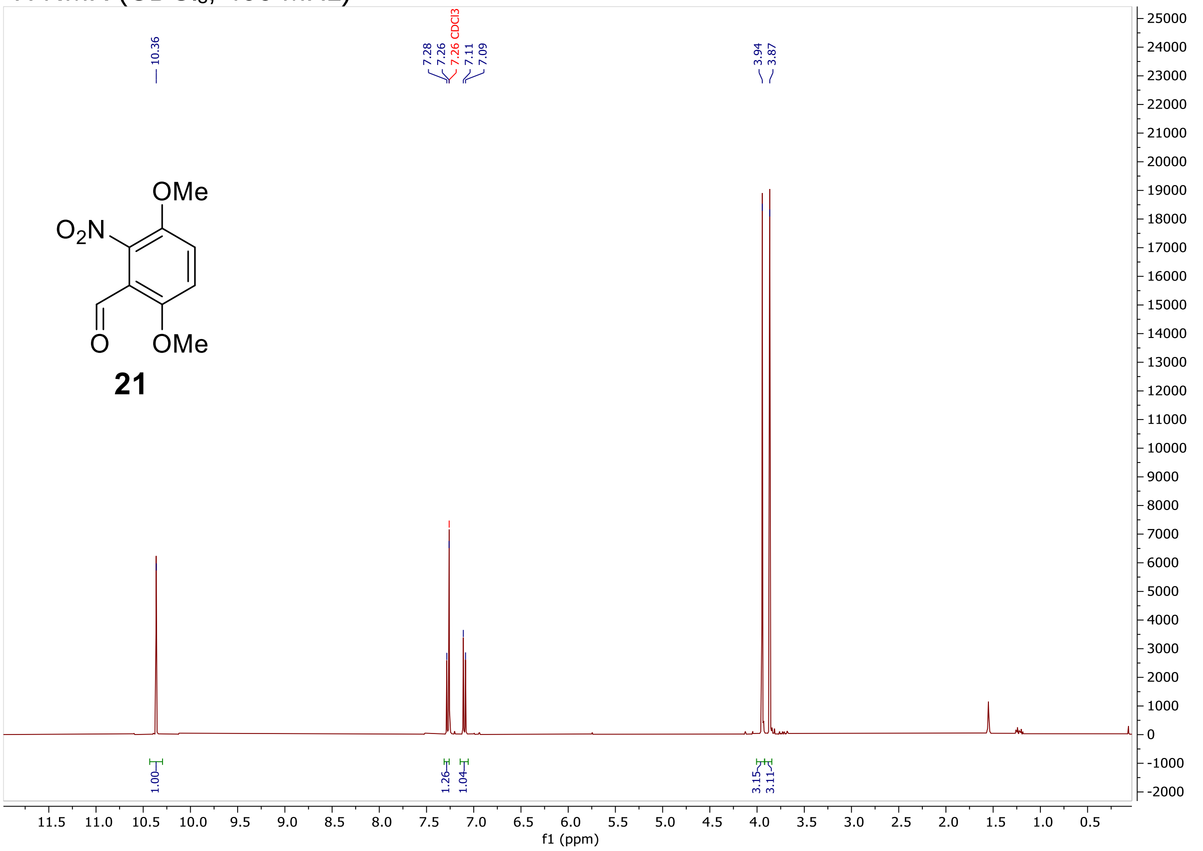

^{13}C {^{1}H} NMR ($CDCl_3$, 101 MHz)

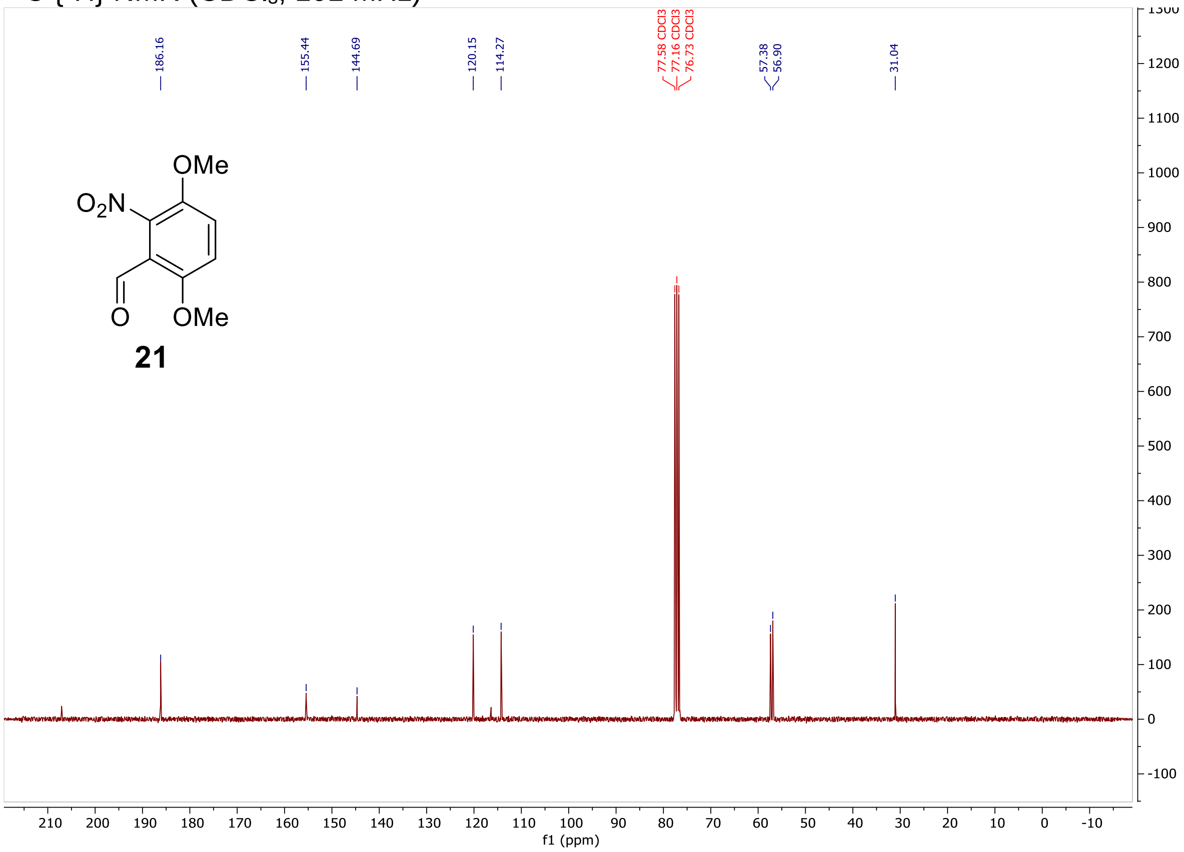

[1,4-bis(methoxymethoxy)-3-methylnaphthalen-2-yl](3,6-dimethoxy-2-nitrophenyl)methanol (**25**)

^{1}H NMR ($CDCl_3$, 400 MHz)

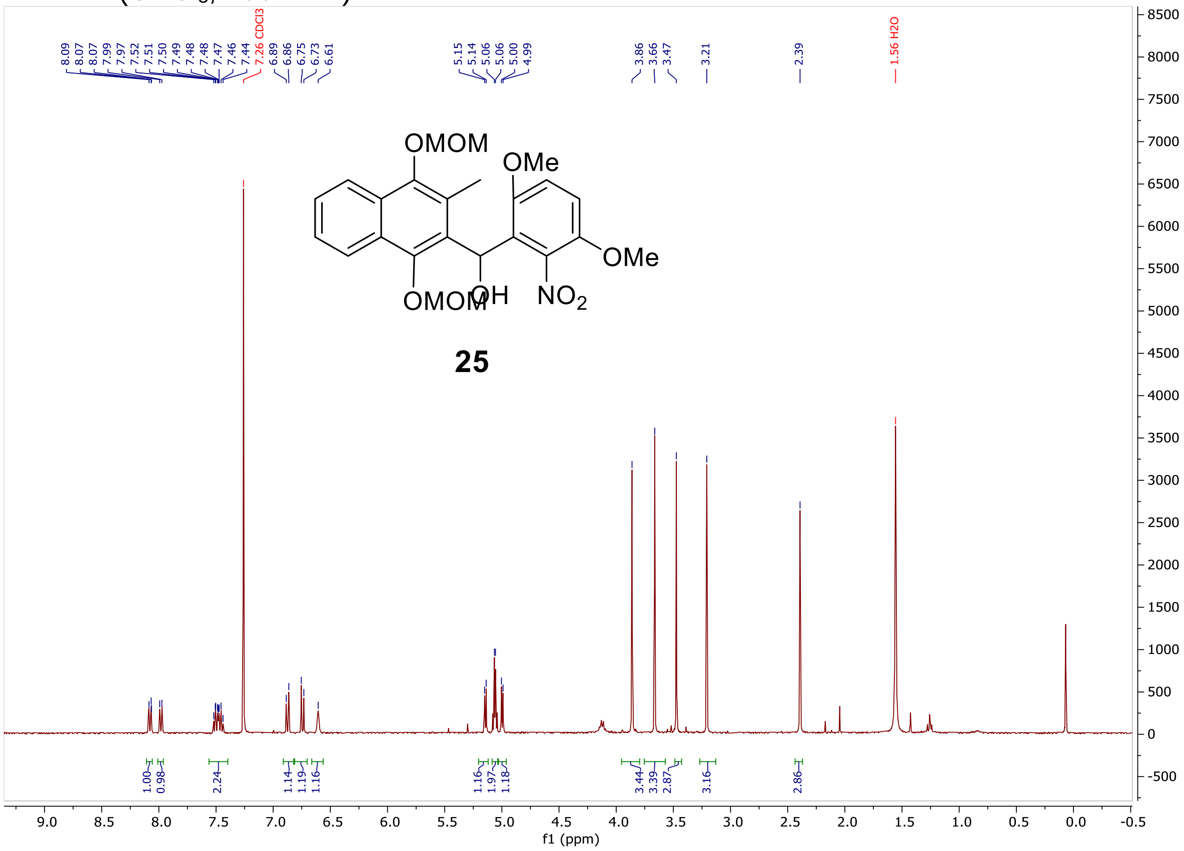

^{13}C {^{1}H} NMR ($CDCl_3$, 101 MHz)

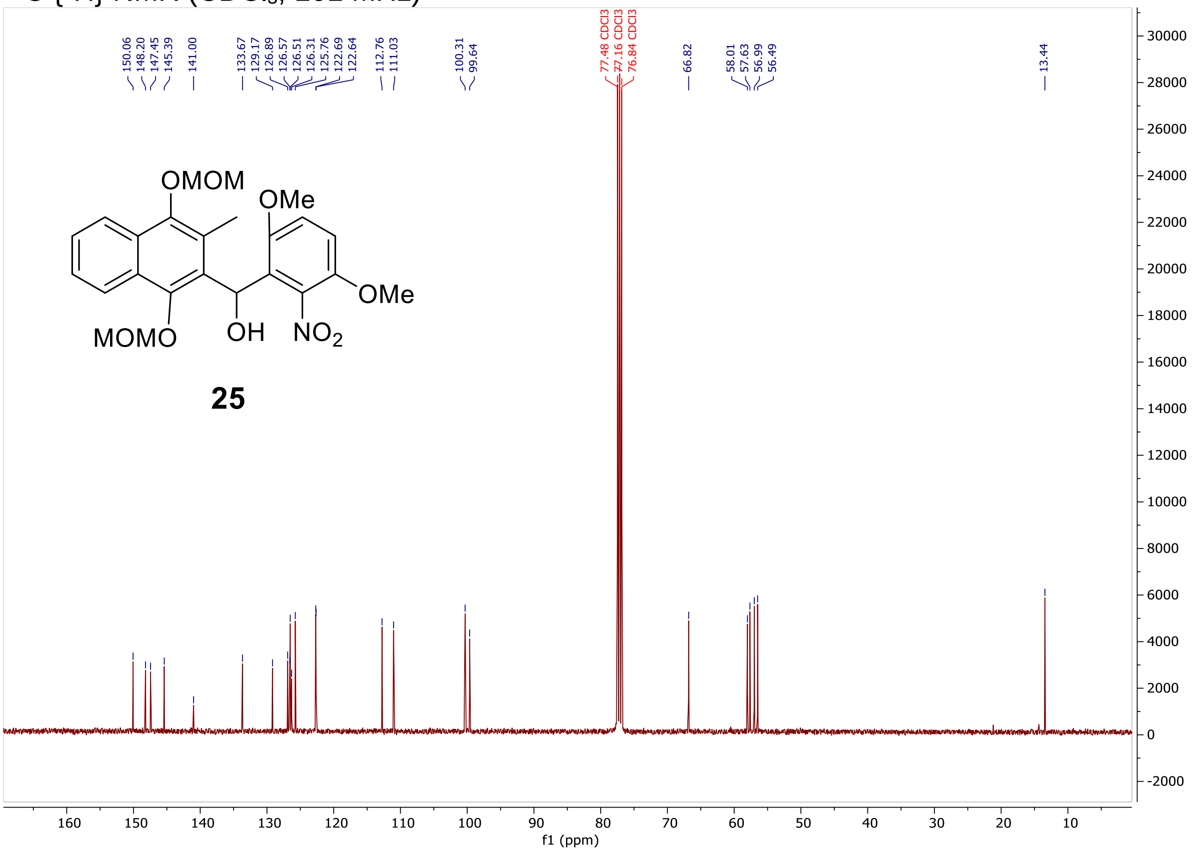

[1,4-bis(methoxymethoxy)-3-methylnaphthalen-2-yl](3,6-dimethoxy-2-nitrophenyl)methanone (**28**)

^{1}H NMR ($CDCl_3$, 400 MHz)

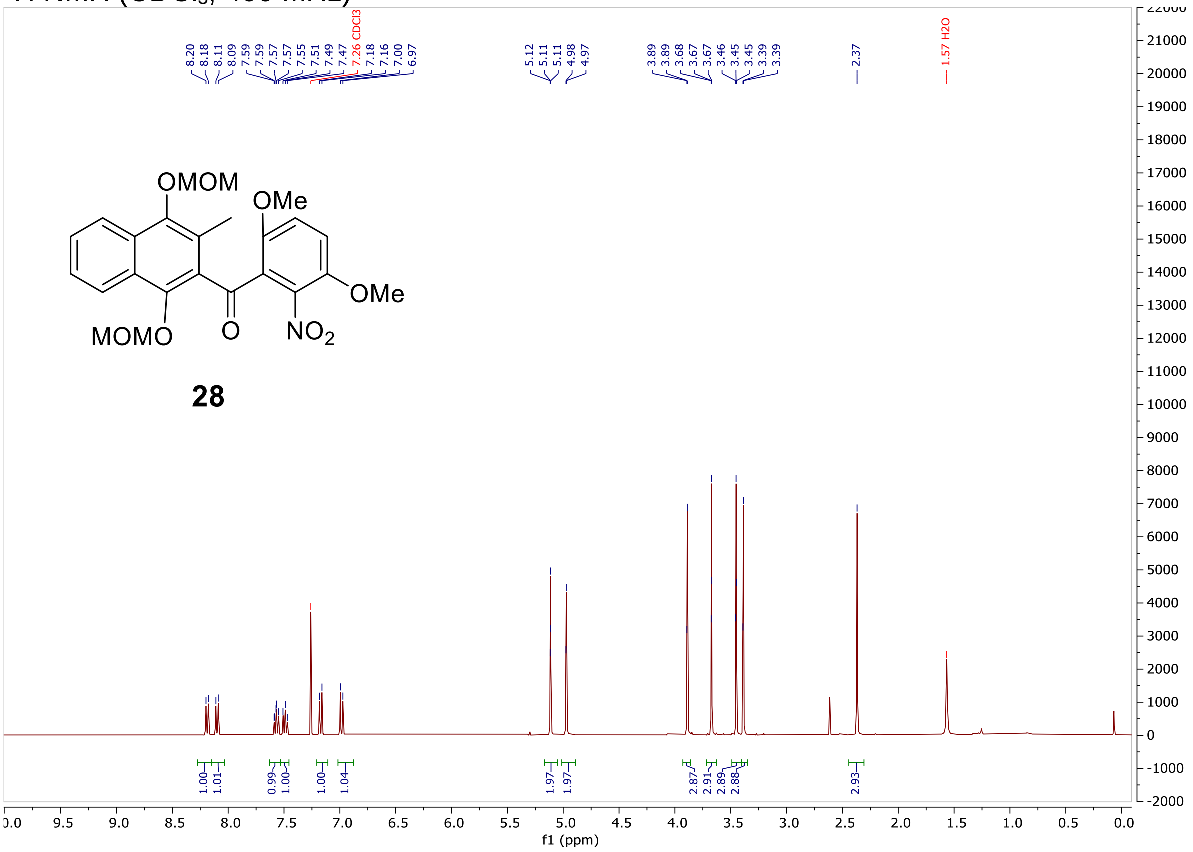

^{13}C {^{1}H} NMR ($CDCl_3$, 101 MHz)

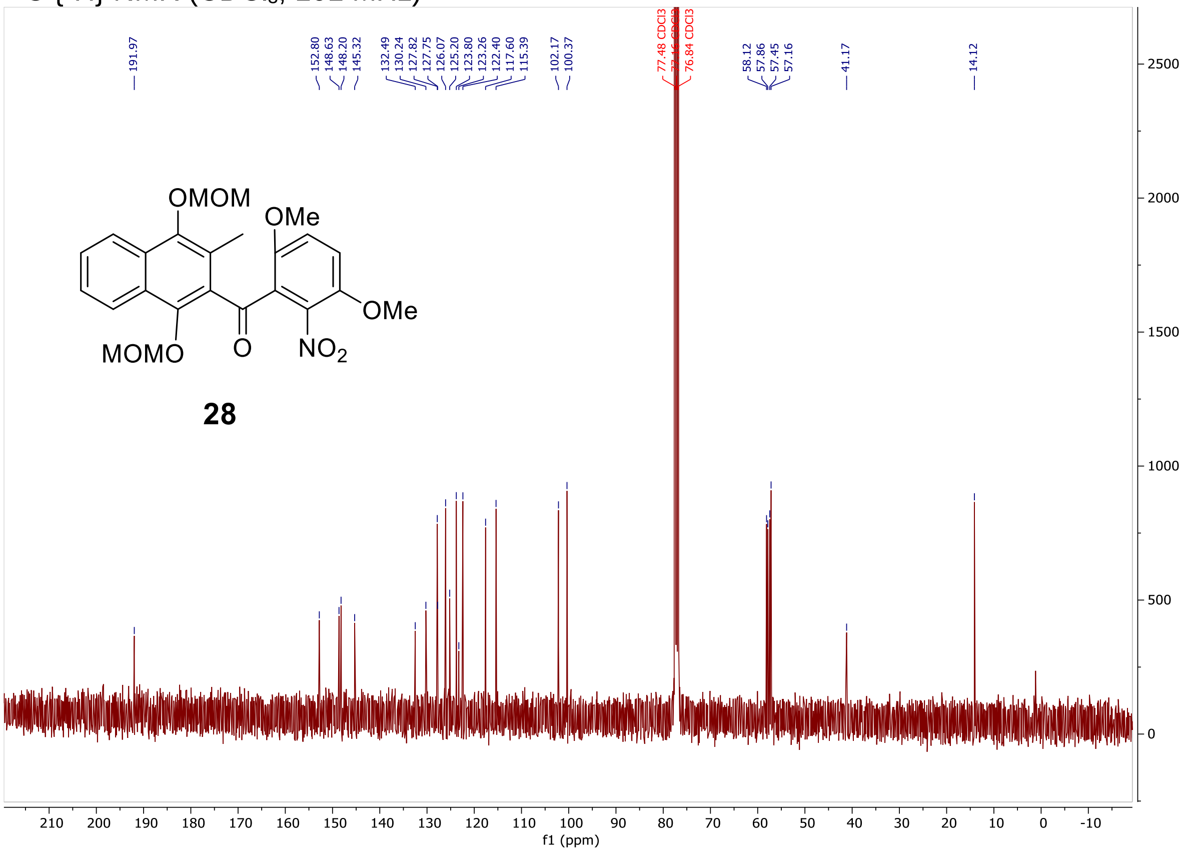

2-[(3,6-dimethoxy-2-nitrophenyl)carbonyl]-4-(methoxymethoxy)-3-methylnaphthalen-1-ol (**29**)

^{1}H NMR ($CDCl_3$, 400 MHz)

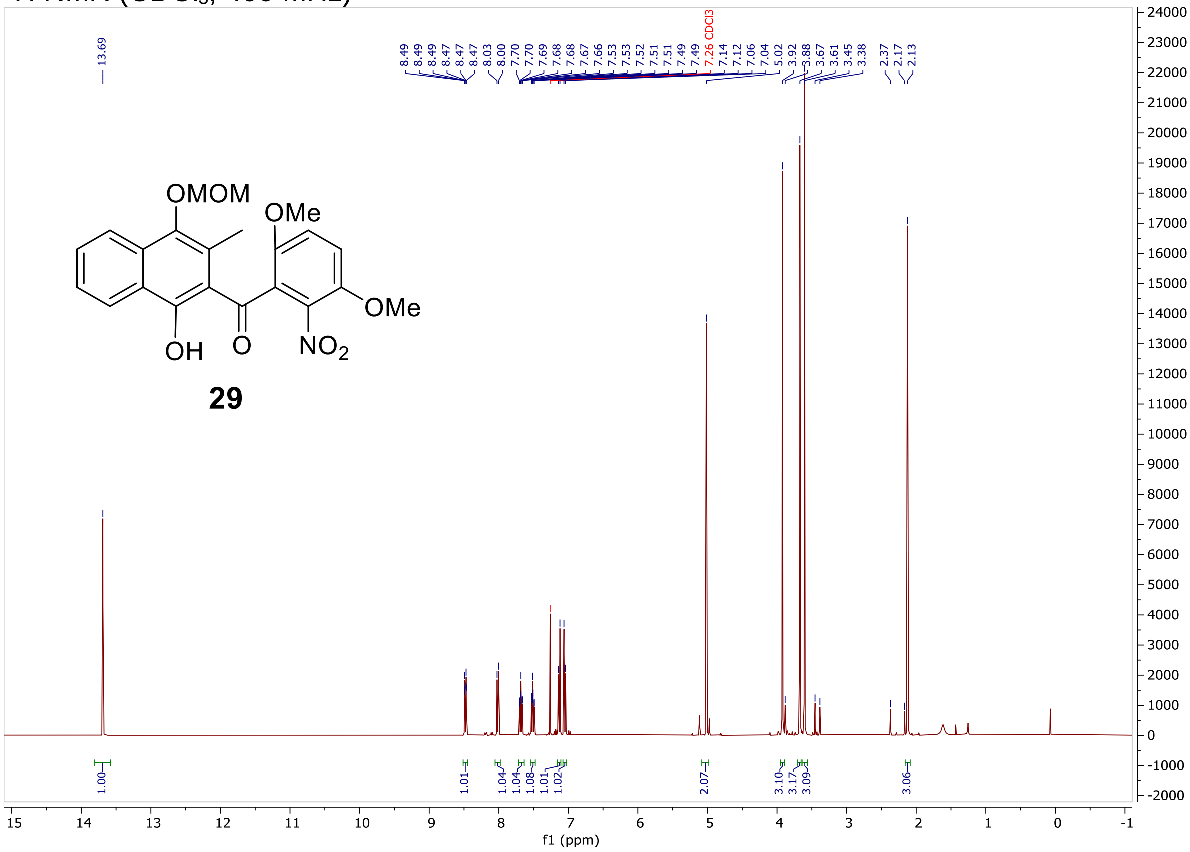

^{13}C {^{1}H} NMR ($CDCl_3$, 101 MHz)

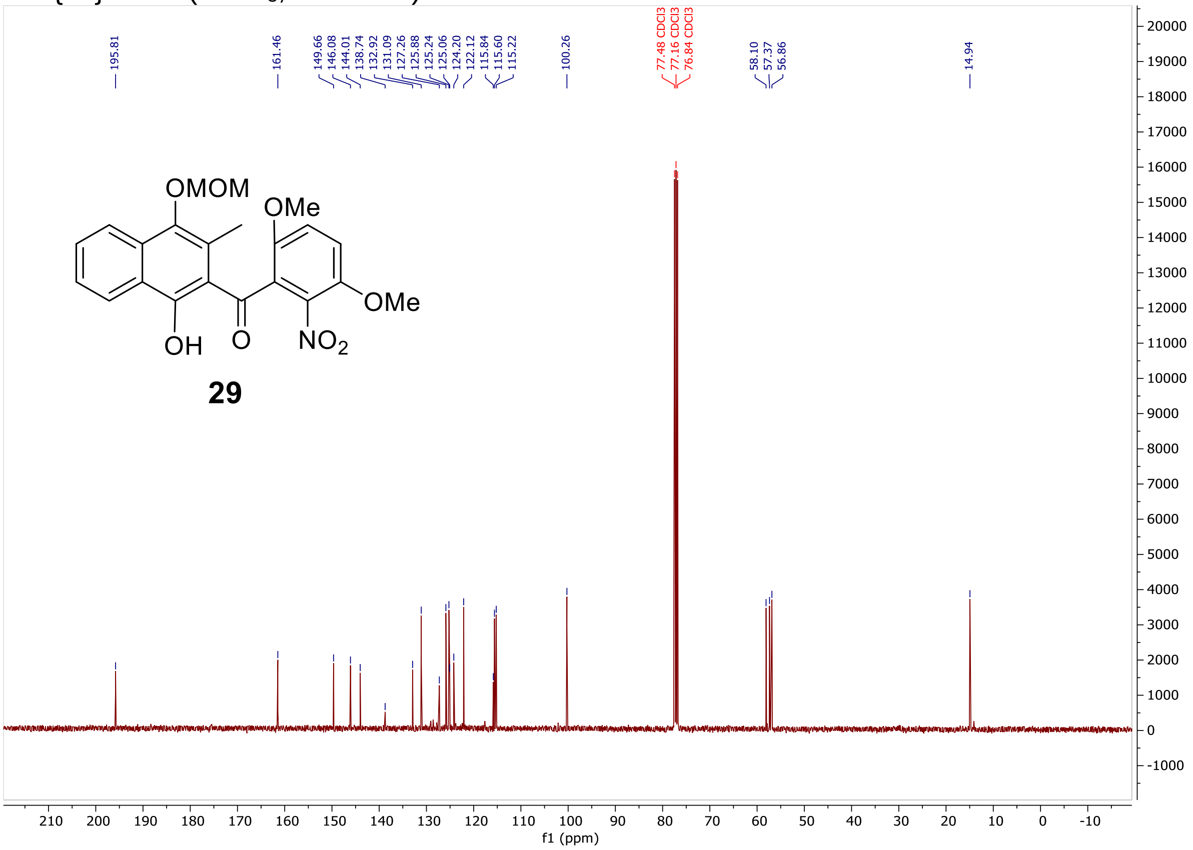

1,4-dimethoxy-10-(methoxymethoxy)-11-methyl-12H-5-oxatetraphen-12-one (**30**)

^{1}H NMR ($CDCl_3$, 400 MHz)

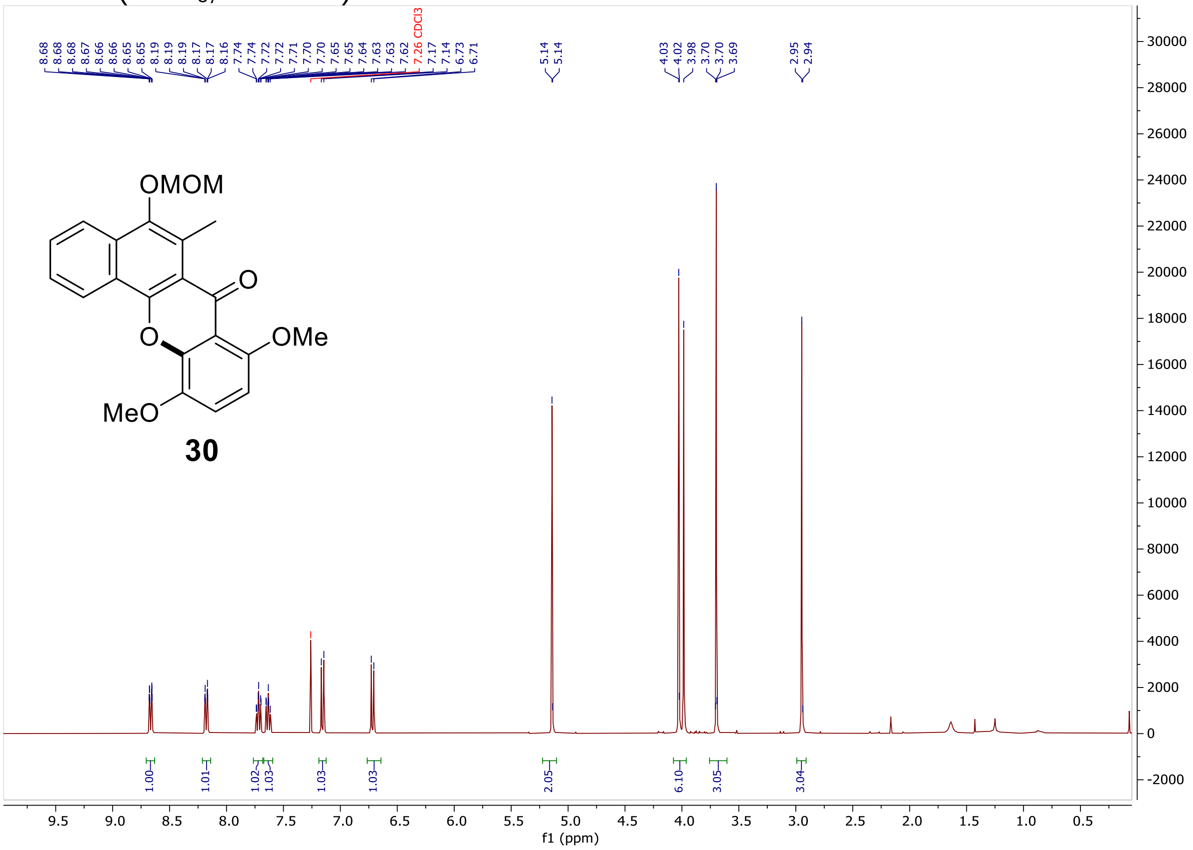

^{13}C {^{1}H} NMR ($CDCl_3$, 101 MHz)

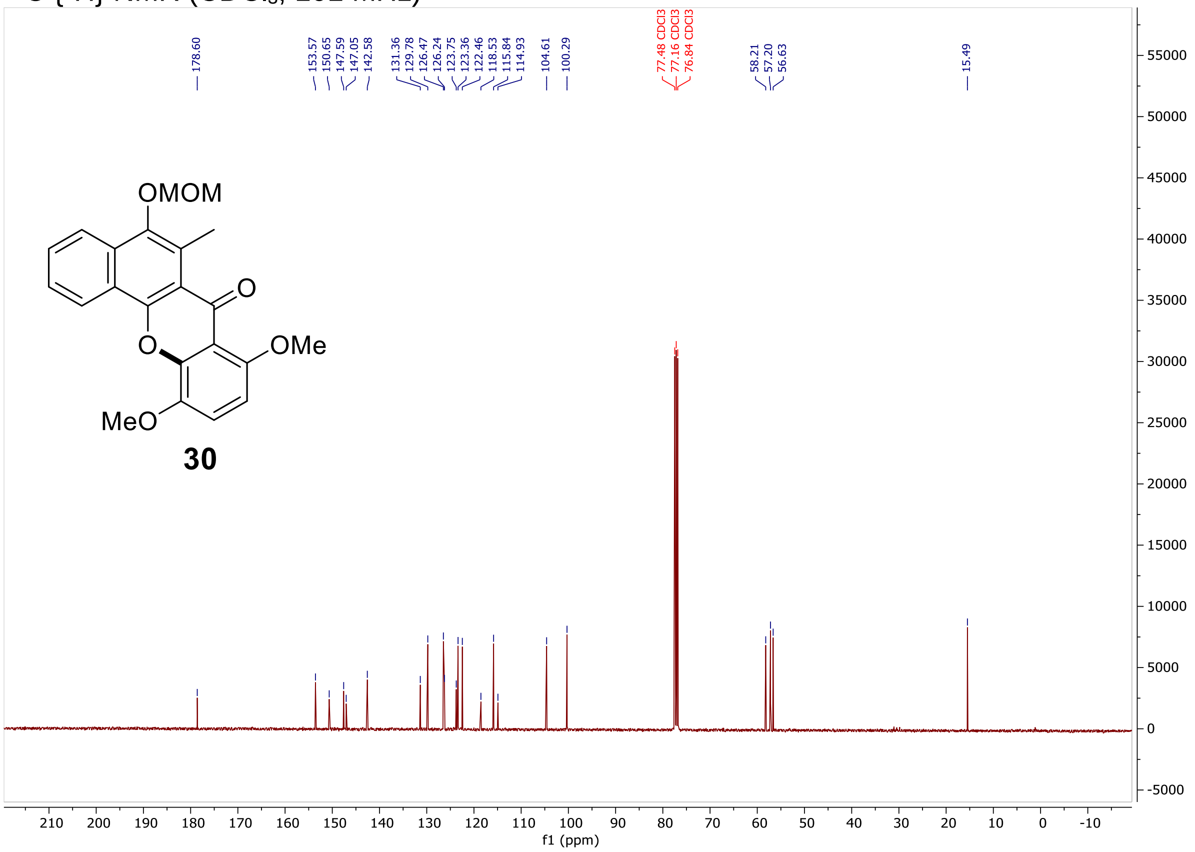

9-methoxy-5-(methoxymethoxy)-6-methyl-8-nitro-7H-benzo[c]xanthen-7-one (**31**)

^{1}H NMR ($CDCl_3$, 400 MHz)

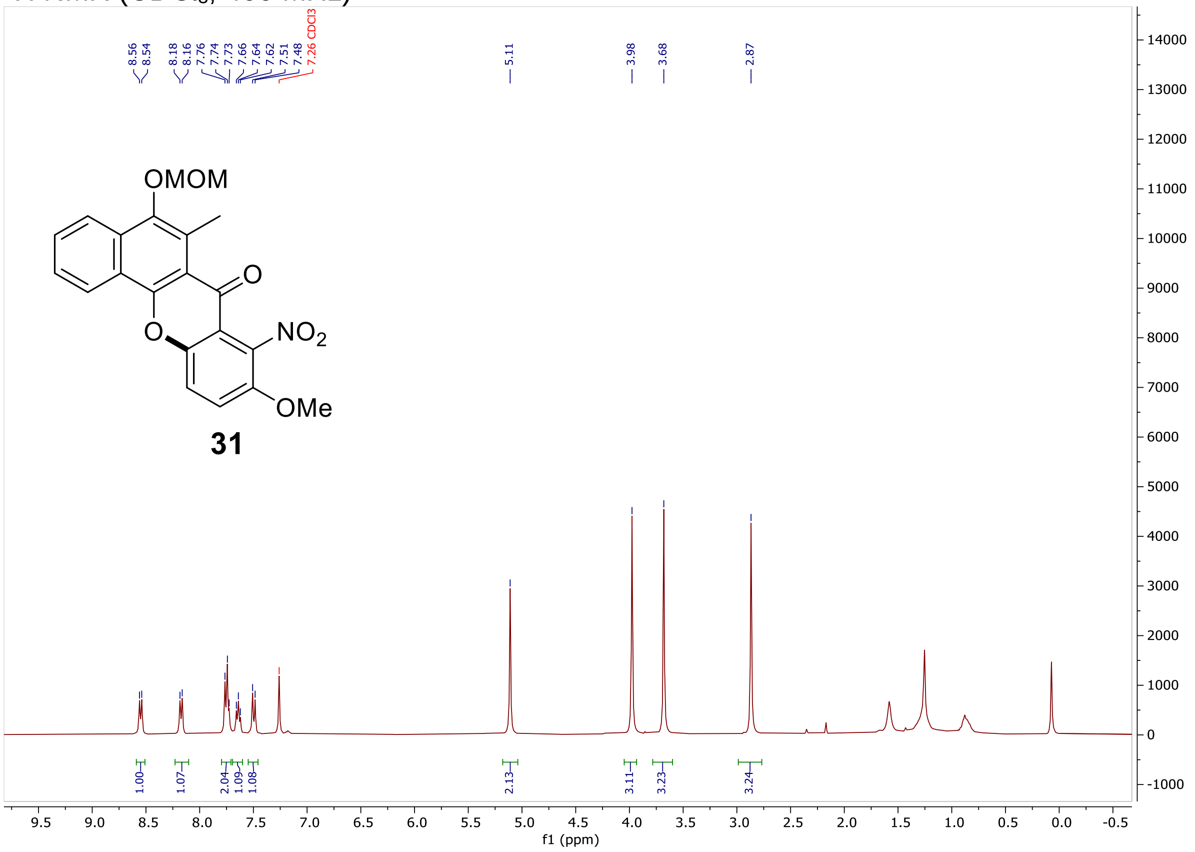

^{13}C {^{1}H} NMR ($CDCl_3$, 101 MHz)

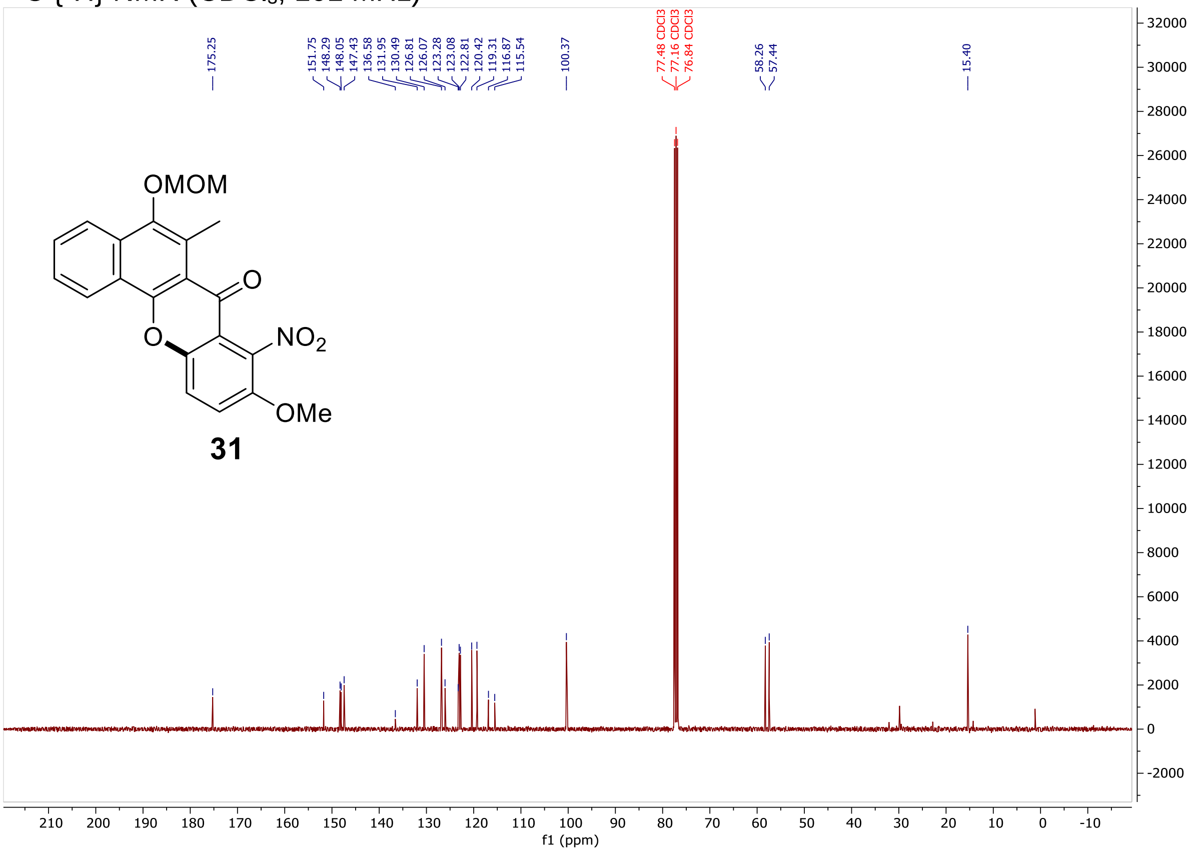

10-hydroxy-1,4-dimethoxy-11-methyl-12H-5-oxatetraphen-12-one (**7**)

^{1}H NMR ($CDCl_3$, 400 MHz)

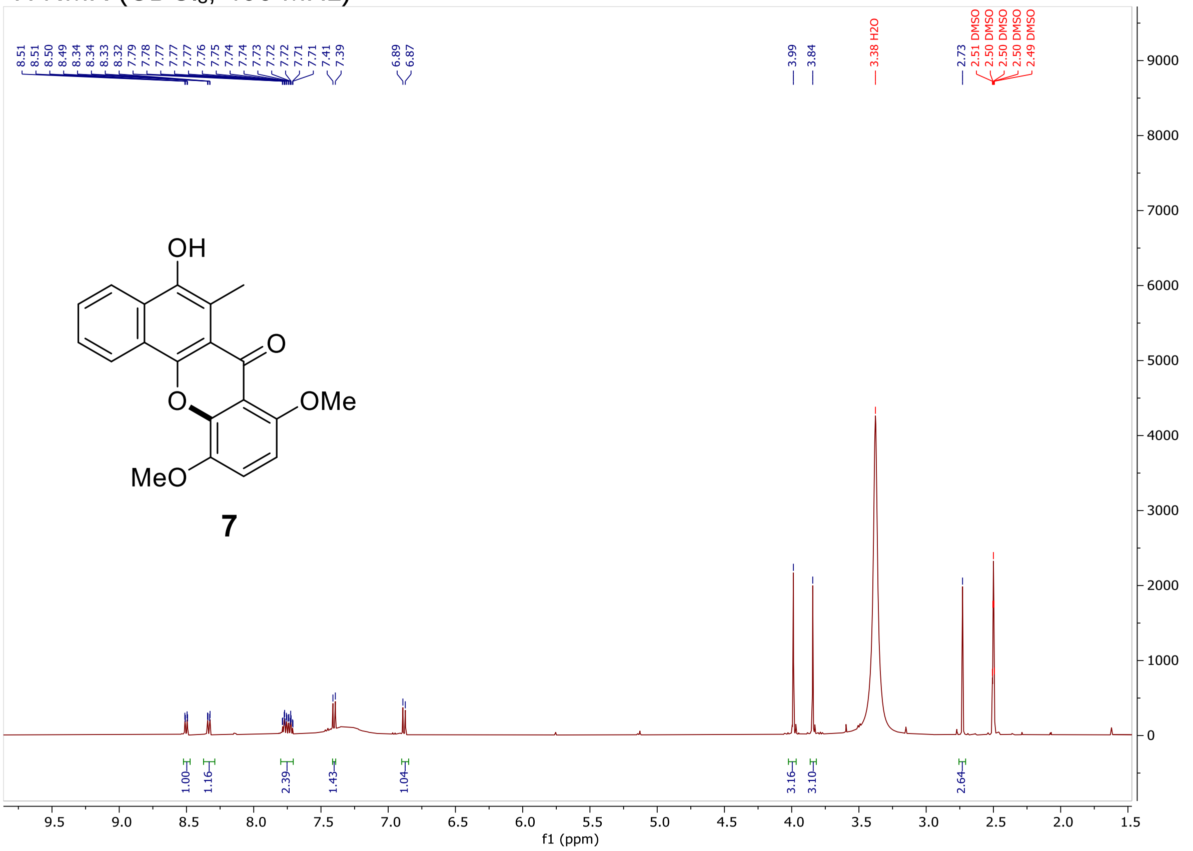

$^{13}C\ \{^{1}H\}$ NMR ($CDCl_3$, 101 MHz)

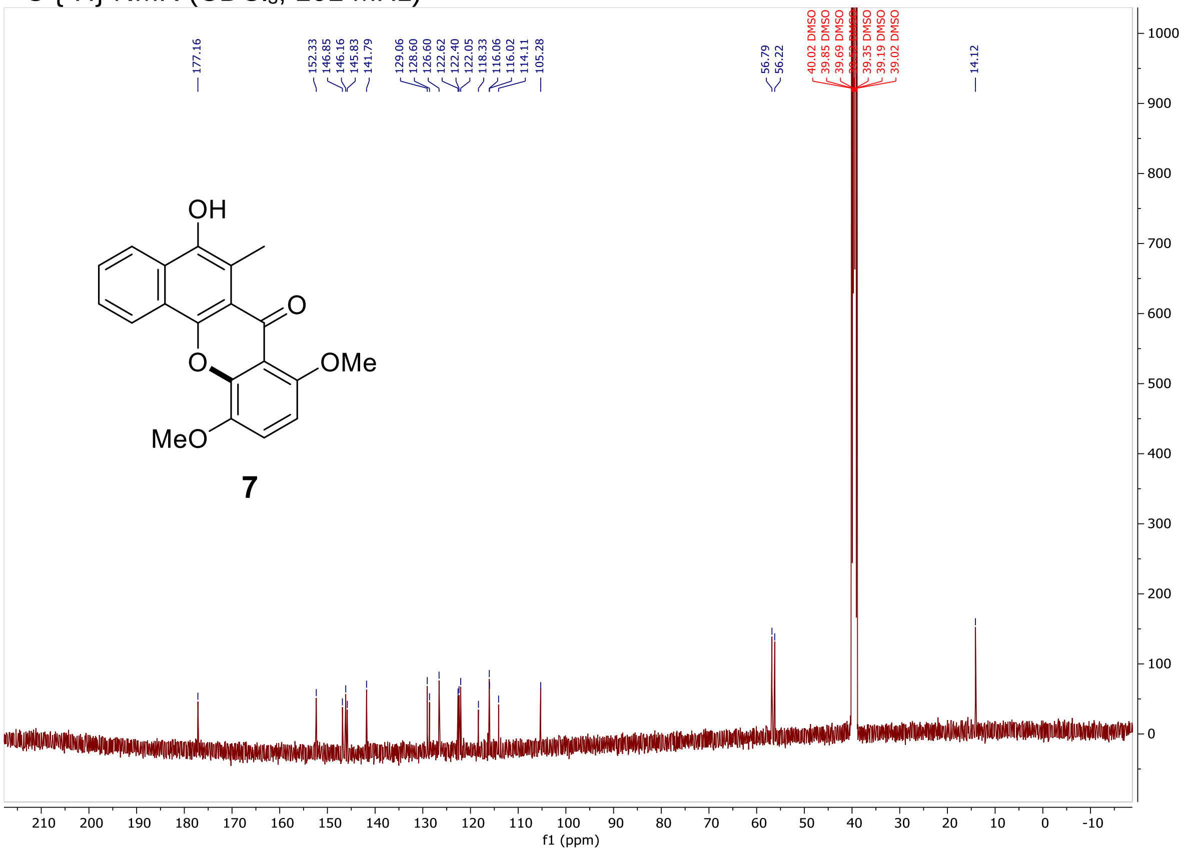

Supplement: Supplementary file 1 [file molecules-31-01839-s001.zip › molecules-4291600-supplementary.pdf]
